# Inflammation and Brain Structure in Schizophrenia and Other Neuropsychiatric Disorders: A Mendelian Randomization Study

**DOI:** 10.1001/jamapsychiatry.2022.0407

**Published:** 2022-03-30

**Authors:** John A. Williams, Stephen Burgess, John Suckling, Paris Alexandros Lalousis, Fatima Batool, Sian Lowri Griffiths, Edward Palmer, Andreas Karwath, Andrey Barsky, Georgios V. Gkoutos, Stephen Wood, Nicholas M. Barnes, Anthony S. David, Gary Donohoe, Joanna C. Neill, Bill Deakin, Golam M. Khandaker, Rachel Upthegrove

**Affiliations:** 1 Institute of Cancer and Genomic Sciences, Centre for Computational Biology, University of Birmingham, Birmingham, United Kingdom; 2 Institute for Translational Medicine, University of Birmingham, Birmingham, United Kingdom; 3 Health Data Research UK (HRD), Midlands Site, Birmingham, United Kingdom; 4 Medical Research Council Biostatistics Unit, Cambridge Institute of Public Health, Cambridge, United Kingdom; 5 Cardiovascular Epidemiology Unit, Department of Public Health and Primary Care, University of Cambridge, Cambridge, United Kingdom; 6 Department of Psychiatry, University of Cambridge, Cambridge, United Kingdom; 7 Institute for Mental Health, University of Birmingham, Birmingham, United Kingdom; 8 Centre for Human Brain Health, University of Birmingham, Birmingham, United Kingdom; 9 Orygen, Melbourne, Australia; 10 Centre for Youth Mental Health, University of Melbourne, Melbourne, Australia; 11 Institute for Clinical Sciences, University of Birmingham, Birmingham, United Kingdom; 12 Institute of Mental Health, University College London, London, United Kingdom; 13 School of Psychology, National University of Ireland Galway, Galway, Ireland; 14 Centre for Neuroimaging, Cognition and Genomics, National University of Ireland Galway, Galway, Ireland; 15 Division of Pharmacy and Optometry, School of Health Sciences, Faculty of Biology, Medicine and Health, University of Manchester, Manchester, United Kingdom; 16 Division of Neuroscience and Experimental Psychology, School of Biological Sciences, Faculty of Biology, Medicine and Health, University of Manchester, Manchester, United Kingdom; 17 MRC Integrative Epidemiology Unit, Population Health Sciences, Bristol Medical School, University of Bristol, Bristol, United Kingdom; 18 Centre for Academic Mental Health, Population Health Sciences, Bristol Medical School, University of Bristol, Bristol, United Kingdom; 19 Avon and Wiltshire Mental Health Partnership NHS Trust, Bristol, United Kingdom; 20 NIHR Bristol Biomedical Research Centre, Bristol, United Kingdom; 21 Early Intervention Service, Birmingham Women’s and Children’s NHS Foundation Trust, Birmingham, United Kingdom

## Abstract

**Importance:**

Previous in vitro and postmortem research suggests that inflammation may lead to structural brain changes via activation of microglia and/or astrocytic dysfunction in a range of neuropsychiatric disorders.

**Objective:**

To investigate the relationship between inflammation and changes in brain structures in vivo and to explore a transcriptome-driven functional basis with relevance to mental illness.

**Design, Setting, and Participants:**

This study used multistage linked analyses, including mendelian randomization (MR), gene expression correlation, and connectivity analyses. A total of 20 688 participants in the UK Biobank, which includes clinical, genomic, and neuroimaging data, and 6 postmortem brains from neurotypical individuals in the Allen Human Brain Atlas (AHBA), including RNA microarray data. Data were extracted in February 2021 and analyzed between March and October 2021.

**Exposures:**

Genetic variants regulating levels and activity of circulating interleukin 1 (IL-1), IL-2, IL-6, C-reactive protein (CRP), and brain-derived neurotrophic factor (BDNF) were used as exposures in MR analyses.

**Main Outcomes and Measures:**

Brain imaging measures, including gray matter volume (GMV) and cortical thickness (CT), were used as outcomes. Associations were considered significant at a multiple testing–corrected threshold of *P* < 1.1 × 10^−4^. Differential gene expression in AHBA data was modeled in brain regions mapped to areas significant in MR analyses; genes were tested for biological and disease overrepresentation in annotation databases and for connectivity in protein-protein interaction networks.

**Results:**

Of 20 688 participants in the UK Biobank sample, 10 828 (52.3%) were female, and the mean (SD) age was 55.5 (7.5) years. In the UK Biobank sample, genetically predicted levels of IL-6 were associated with GMV in the middle temporal cortex (*z* score, 5.76; *P* = 8.39 × 10^−9^), inferior temporal (*z *score, 3.38; *P* = 7.20 × 10^−5^), fusiform (*z *score, 4.70; *P* = 2.60 × 10^−7^), and frontal (*z *score, −3.59; *P* = 3.30 × 10^−5^) cortex together with CT in the superior frontal region (*z *score, −5.11; *P* = 3.22 × 10^−7^). No significant associations were found for IL-1, IL-2, CRP, or BDNF after correction for multiple comparison. In the AHBA sample, 5 of 6 participants (83%) were male, and the mean (SD) age was 42.5 (13.4) years. Brain-wide coexpression analysis showed a highly interconnected network of genes preferentially expressed in the middle temporal gyrus (MTG), which further formed a highly connected protein-protein interaction network with IL-6 (enrichment test of expected vs observed network given the prevalence and degree of interactions in the STRING database: 43 nodes/30 edges observed vs 8 edges expected; mean node degree, 1.4; genome-wide significance, *P* = 4.54 × 10^−9^). MTG differentially expressed genes that were functionally enriched for biological processes in schizophrenia, autism spectrum disorder, and epilepsy.

**Conclusions and Relevance:**

In this study, genetically determined IL-6 was associated with brain structure and potentially affects areas implicated in developmental neuropsychiatric disorders, including schizophrenia and autism.

## Key Points

**Question:**

Is there evidence for a potential relationship between inflammation and brain structure, and is this relevant for schizophrenia and other neuropsychiatric disorders?

**Findings:**

In this mendelian randomization study including 20 688 participants in the UK Biobank, genetically predicted levels of interleukin 6 were associated with gray matter volume and cortical thickness primarily in the middle temporal gyrus and superior frontal region. The middle temporal gyrus overexpressed a number of genes relevant to interleukin 6 pathway proteins and neuropsychiatric disorder ontologies, including schizophrenia and autism spectrum disorder.

**Meaning:**

This study found that inflammation may be associated with brain structure and may be an early predeterminant of neuropsychiatric conditions, which has important implications for identification of risk and novel treatments.

## Teaser

This mendelian randomization study investigates the relationship between inflammation and changes in brain structures in vivo and explores a transcriptome-driven functional basis with relevance to mental illness.

## Introduction

Numerous avenues of inquiry suggest a relationship between immune dysfunction and psychiatric disorders, including schizophrenia, autism spectrum disorders, and depression.^1^ There is robust evidence for increased circulating concentrations of proinflammatory cytokines before the onset of illness,^2^ and epidemiological studies have shown that exposure to a variety of infections during prenatal life and childhood are associated with increased risk of schizophrenia and autism spectrum disorder.^3,4,5^ Recent analyses using mendelian randomization (MR) suggest potential causality between inflammatory cytokines and schizophrenia and depression.^6^ This background supports theories of maternal immune activation of early inflammatory processes and a 2-hit model with subsequent environmental factors triggering nonresolving inflammation and the precipitation of psychiatric disorders.^7,8,9,10^

Patients with mental disorders show a range of differences in structural brain measures compared with healthy controls, but the cause of these differences remains ncertain.^2^ Interleukin 6 (IL-6) and its receptor IL-6R could be of particular interest as it is able to cross the blood-brain barrier and increase its permeability, drawing in further local inflammatory actors,^11^ and may be related to treatment resistance and poor functional outcomes.^12,13^ Inflammation is implicated in structural brain changes underlying neuropsychiatric disorders via microglia and astrocytic function with disordered synaptic pruning and subsequent effect on gray matter volume (GMV).^14^ Immune glial dysfunction may differentially influence risk of mental health disorders; for example, radioligands for translocator protein, a marker of microglial activation, is reduced in medication-naive patients with psychosis but increased in patients with depression.^2^ Mental health disorders are highly comorbid, suggesting a potentially common inflammatory mediated mechanistic pathway for a subgroup of patients.^15,16,17,18^

There are relatively few studies exploring the association between IL-6 and related markers and structural brain changes in patient samples in vivo. In patients with depression and inflammation, GMV alterations have been reported in the temporal, orbitofrontal and inferiofrontal, and cingulate regions.^19,20^ In psychosis, GMV loss and cortical thinning have been related to elevation of immune-proteomic markers in temporal, prefrontal, and cingulate areas.^21^ Studies to date are often based on relatively small samples, patient populations with long-term disease, are cross-sectional in nature, or confounded by medication and environmental factors. To our knowledge, MR, which is able to control for environmental confounds, has not previously been used to investigate this association.^22^ Transcriptomic profiling of brain regions neuropsychiatric populations have attempted to link inflammatory and neuropsychiatric gene function, with mixed results also potentially related to confounds.^23,24^

A clearer understanding of the association between immune dysfunction and brain structure, with evidence of potential causal inference and relevance to mental health disorders, would be a significant advance and allow a deeper understanding of early causal pathways, offering the potential for more refined targeting of novel treatments.^25^ We aimed to test for evidence of potential causality in the association between inflammatory cytokines and brain structure using MR, with genetically predicted levels of cytokine activity as proxies for exposure. Subsequently, we interrogated gene expression in immune-related brain regions and tested the relevance of gene expression patterns in neuropsychiatric disorders.

We hypothesized that genetically predicted increased IL-6 and IL-6R activity would be associated with reduced gray matter volume and cortical thickness (CT) in areas highly relevant to neuropsychiatric disorders, including schizophrenia, autism spectrum disorder, and depression. We further expected genes overexpressed in identified regions to participate in biological processes relevant to neuropsychiatric disorders as explored in human biomedical databases and rodent models.

## Methods

**Figure 1.**
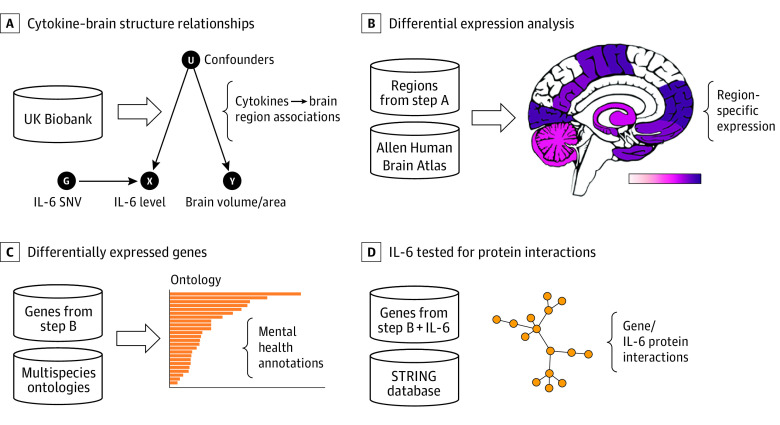
Study Workflow Each panel contains data (cylinders) input into processes, resulting in associations (brackets). A, Cytokine–brain structure associations were investigated with mendelian randomization. B, Differential expression analysis was performed between the whole brain and brain regions with interleukin 6 (IL-6) associations. C, Differentially expressed genes were enriched for functional and disease characteristics. D, IL-6 was tested for protein interactions in the STRING protein-protein interaction database version 11 (STRING Consortium) with differentially expressed genes. SNV indicates single-nucleotide variant.

Methods are summarized in Figure 1 and the eMethods in Supplement 1, which include the Strengthening the Reporting of Observational Studies in Epidemiology Using Mendelian Randomization (STROBE-MR) checklist. Briefly, first, we used MR to test associations of genetic predictors of levels of a range of cytokines and acute phase proteins with variation in GMV and CT. We then investigated which genes were differentially expressed in brain regions significantly indicated in MR analyses. Region-specific gene sets were functionally investigated to assess how transcriptional activity in these brain regions may manifest as neuropsychiatric function, and genes functionally interacting with the IL-6 or IL-6R pathway characterized in relation to neuropsychiatric disorders. All participants provided written informed consent. UK Biobank received ethical approval from the North West Multi-centre Research Ethics Committee. The present analyses were conducted under UK Biobank application number 26999.

Genetic data and neuroimaging-derived phenotypes (outcomes) were taken from the UK Biobank.^26^ We investigated genetic predictors of 5 available exposures associated with risk of neuropsychiatric disorders in MR analyses^6^ (IL-1,^27^ IL-2^28^, IL-6,^29^ C-reactive protein [CRP^30^], and brain-derived neurotrophic factor [BDNF^31^]) to examine specificity of findings for the IL-6 and IL-6R pathway. For each inflammatory biomarker, we selected genetic variants in a relevant coding gene region previously shown to be conditionally associated with the inflammatory biomarker and moderately correlated (*r*^2^ < 0.6) (eMethods in Supplement 1). We considered 1436 outcomes derived from T1-weighted magnetic resonance imaging (MRI) from 20 688 individuals in the imaging subset of the UK Biobank study, described previously.^32,33^

We performed 2-sample MR analyses using the inverse-variance weighted method, including estimated genetic correlations from a reference population, using genetic associations with the inflammatory biomarkers obtained from the literature (eMethods and eTable 2 in Supplement 1), and genetic associations with the outcomes from UK Biobank correcting for physical, genetic, and technical covariates. To present results for diverse traits on a common scale, estimates are divided by their standard errors and reported as *z* scores, where a positive *z* score represents genetically predicted levels of the biomarker that were positively associated with the brain imaging measure. The number of independent hypotheses tested was estimated by principal component analysis, which indicated that 95% of the variation in the outcome data was explained by 442 principal components. Associations were therefore considered significant at a multiple testing–corrected threshold of *P* < 1.1 × 10^−4^ (.05/442) from 2-tailed inverse variance–weighted MR (eMethods in Supplement 1).

From the UK Biobank, the regional imaging measures were CT and brain volume extracted from available parcellation atlases. Brain imaging results from MR analysis were imported into the MarsBaR toolbox^29^ to aid visualization and identify those brain regions of interest (ROIs) in SPM (Wellcome Centre for Human Neuroimaging) that were statistically significantly associated with genetically predicted levels of biomarkers (eMethods in Supplement 1).

We analyzed gene expression from data in the Allen Human Brain Atlas (AHBA),^34^ which annotates 1839 segmented regions in its atlas, and a combination of measurements segmented in the mammalian Allen Brain Atlas (ABA). Where available, we conducted experiments to compare whole-brain expression to gene expression in brain ROIs indicated in MR results, as not all brain regions have corresponding probes in the microarray experiments. In a data-driven approach, differential expression analysis was performed between expression in each indicated brain region and whole-brain expression levels. Overexpressed genes were examined for enrichment in the Gene Ontology’s biological process domain,^35^ disease ontology,^36^ and mammalian phenotype ontology.^37^ Genes differentially expressed in significant regions were tested for protein interactions with IL-6 in STRING version 11 (STRING Consortium).^38^

## Results

Of 20 688 participants in the UK Biobank sample, 10 828 (52.3%) were female, and the mean (SD) age was 55.5 (7.5) years. In the AHBA sample, 5 of 6 participants (83%) were male, and the mean (SD) age was 42.5 (13.4) years. A full sample description can be found in eTable 1 in Supplement 1.

### Association of Biomarkers With Brain Structure

**Figure 2.**
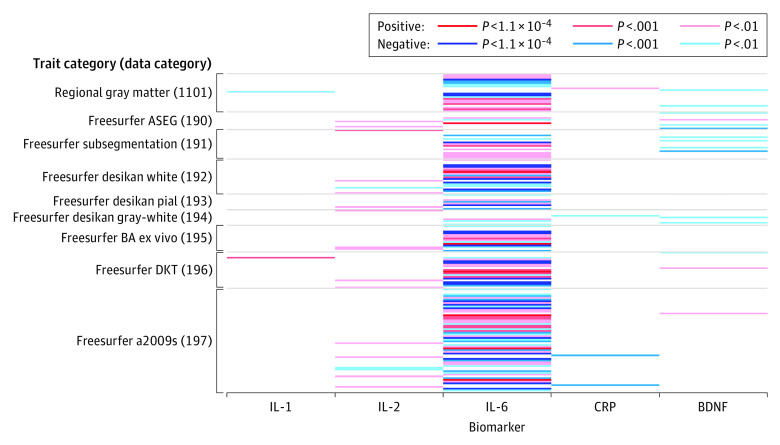
Heat Map of Associations Between Genetically Predicted Inflammatory Biomarkers and Brain Imaging Measures Brain imaging measures from UK Biobank, with data categories from the UK Biobank Brain Imaging Catalogue, and their association with inflammatory biomarkers are shown. Brain imaging measures having no associated inflammatory biomarkers at *P* < .01 are omitted from presentation; only the 166 measures with at least 1 associated biomarker are plotted. BDNF indicates brain-derived neurotrophic factor; CRP, C-reactive protein; IL-1, interleukin 1; IL-2, interleukin 2; IL-6, interleukin 6.

Associations of genetically predicted values of the 5 investigated biomarkers with the brain imaging measures are displayed as a heat map in Figure 2. In total, genetically predicted IL-6 levels were associated with 33 brain imaging measures of GMV or CT after correction for multiple testing (*P* < 1.1 × 10^−4^). No genetically predicted levels of other exposures (IL1, IL2, CRP, or BDNF) were associated with brain imaging measures after correction for multiple testing; at an association of interest (uncorrected *P* < .001), 65, 1, 1, 2, and 2 brain imaging measures were associated with genetically predicted IL-6, IL-1, IL-2, CRP, and BDNF, respectively. A list of traits associated with genetically predicted IL-6 at an uncorrected significance threshold is provided in eTables 3 and 5 in Supplement 1. Additional heat map visualizations of associations between brain imaging measures and inflammatory biomarkers are provided in eTables 4 and 6 and eFigures 1 to 9 in Supplement 1. Results remained unchanged when excluding 216 participants who reported a neuropsychiatric diagnosis and on adjustment for whole-brain volume (eTables 1, 5, and 6 in Supplement 1).

### Mapping of Brain Structure Associated With IL-6

**Figure 3.**
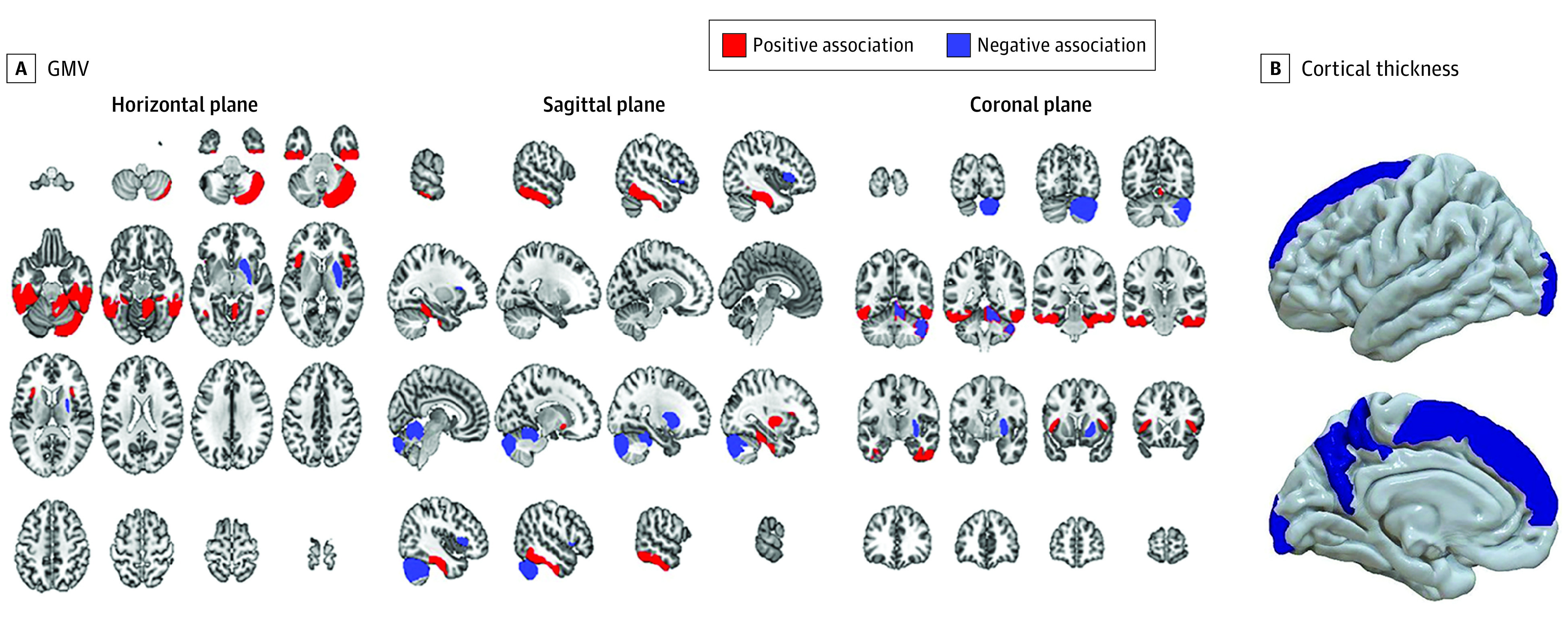
Brain Imaging Measures Associated With Genetically Predicted Levels of Interleukin 6 and Interleukin 6 Receptor Through Mendelian Randomization A, Genetic association of interleukin 6 and its receptor with gray matter volume (GMV) (Harvard-Oxford cortical and subcortical atlas and probabilistic mendelian randomization atlas of the human cerebellum): middle temporal gyrus, temporooccipital part (right: *z* score, 5.76; *P* = 8.40 × 10^−9^), and temporal fusiform cortex, posterior division (right: *z* score, 4.70; *P* = 2.60 × 10^−7^; left: *z* score, 4.20; *P* = 2.67 × 10^−6^), at a multiple testing–corrected threshold of *P* < 1.1 × 10^−4^. Additional measures with *P* < .001 include the inferior temporal gyrus, posterior divisions (right: *z* score, 3.38; *P* = 7.20 × 10^−5^; left: *z* score, 3.73; *P* = 1.90 × 10^−5^), frontal operculum cortex (right: *z* score, −3.59; *P* = 3.30 × 10^−5^), putamen (right: *z* score, −3.78; *P* = 1.60 × 10^−5^), and regions I to IV of the cerebellum vermus (right: *z* score, −3.64; *P* = 2.70 × 10^−5^). B, Cortical thickness (Destrieux cortical atlas): frontal superior (left: *z* score, −5.11; *P* = 3.22 × 10^−7^). Additional measures with *P* < .001 include the G-precuneus (left: z score, −3.59; *P* = 3.30 × 10^−5^), pole-occipital (left: *z* score, −3.61; *P* = 3.10 × 10^−5^), S-parieto-occipital (left: *z* score, −3.34; *P* = 8.40 × 10^−5^), and S-pericallosal (right: *z* score, 3.32; *P* = 9.00 × 10^−5^). Estimates are reported as *z* scores, where a positive *z* score represents that genetically predicted levels of the biomarker were positively associated with the brain imaging measure. Red color denotes a positive association and blue color denotes a negative association. See the eDiscussion in Supplement 1 for further details.

Genetically predicted IL-6 activity was associated with 33 spatially overlapping MRI measures. When mapped into SPM space,^39^ these generated brain ROIs of GMV in the middle temporal gyrus, temporooccipital part (right: *z* score, 5.76; *P* = 8.39 × 10^−9^), and the temporal fusiform cortex, posterior division (right: *z* score, 4.70; *P* = 2.60 × 10^−7^; left: *z* score, 4.20; *P* = 2.67 × 10^−6^), as well as of CT in the frontal superior (left: *z* score, −5.11; *P* = 3.22 × 10^−7^) were significant. The middle temporal gyrus (MTG), fusiform gyrus (FuG), and superior frontal gyrus demonstrated the strongest associations with genetically predicted IL-6 (eMethods and eTable 16 in Supplement 1; Figure 3).

### Differential Gene Expression in Brain Regions Associated With IL-6

**Figure 4.**
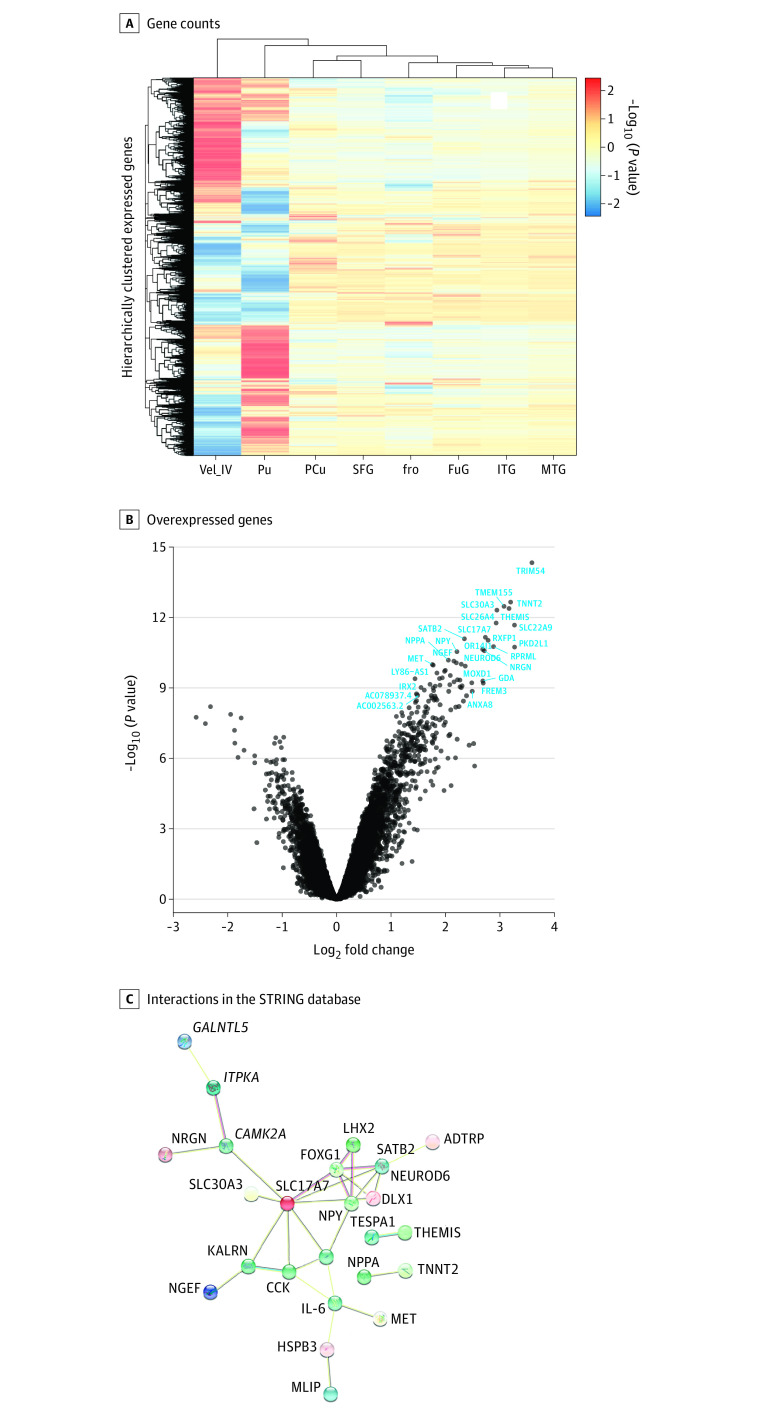
Genes Differentially Overexpressed in the Middle Temporal Gyrus (MTG) and Interleukin 6 A, *z* Score normalized mean gene counts in each region of interest show little within-region variation within genes expressed in the MTG compared with other regions, particularly compared with the cerebellar regions, which show pronounced variation compared with the whole brain. Each row represents one gene’s expression. B, Compared with the whole brain (mean non-MTG values), 47 genes were highly overexpressed (false discovery rate–corrected *P* < .05; log-fold change >2) in the MTG specifically. C, Protein products of these genes plus interleukin 6 were highly enriched for interactions (connectivity *P* = 4.54 × 10^−9^) compared with the frequency of interactions in the STRING database, as measured in the sum of unweighted degrees in the network. Edge colors represent different evidence underlying predicted protein-protein interactions in the STRING database. Images within spheres represent known protein structures; node color is aesthetic. fro Indicates operculum; FuG, fusiform gyrus; ITG, inferior temporal gyrus; MTG, middle temporal gyrus; PCu, precuneus; Pu, putamen; SFG, superior frontal gyrus; Vel_IV, cerebellar vermis.

We conducted experiments to compare whole-brain expression to the brain ROIs: MTG, ITG, fusiform gyrus, and a combination of measurements segmented in the mammalian ABA (cerebellar vermis [Vel_IV] lobules I-II, III, and IV). The *z* score–normalized expression of probes uniquely mapped to each ROI indicated relatively stable expression in the MTG and ITG compared with cerebellar regions (VeI I to V) and the putamen (Figure 4A). Differentially expressed genes in the MTG (false discovery rate–corrected *P* < .05; log-fold change >2) are shown in Figure 4B. While many genes were overexpressed in the MTG compared with the whole brain (mean [SD] log-fold change, 2.47 [0.40]; max log-fold change, 3.59; mean [SD] β, 13.41 [4.37]), none were significantly underexpressed. Differential expression analyses are further described in eTables 7 to 14 and eFigures 10 to 16 in Supplement 1.

### Interaction Between IL-6 and Proteins Differentially Expressed in the MTG

Subsequent enrichment with the STRING protein-protein interaction database (Figure 4C) revealed a highly interconnected network of genes preferentially expressed in the MTG, suggesting these genes act in concert on the protein as well as transcript level (43 nodes/30 edges observed vs 8 edges expected; mean node degree, 1.4; genome-wide significance, *P* = 4.54 × 10^−9^). IL-6 itself was not found to be differentially expressed in the MTG. However, several differentially expressed genes form an interaction network with IL-6. Among these are neuropeptide Y (NPY), met proto-oncogene (MET), cholecystokinin (CCK), muscular LMNA–interacting protein (MLIP), and heat shock protein family B (small) member 3 (HSPB3).

### Associations Between MTG-Enriched Genes and Neuropsychiatric Disorders

**Figure 5.**
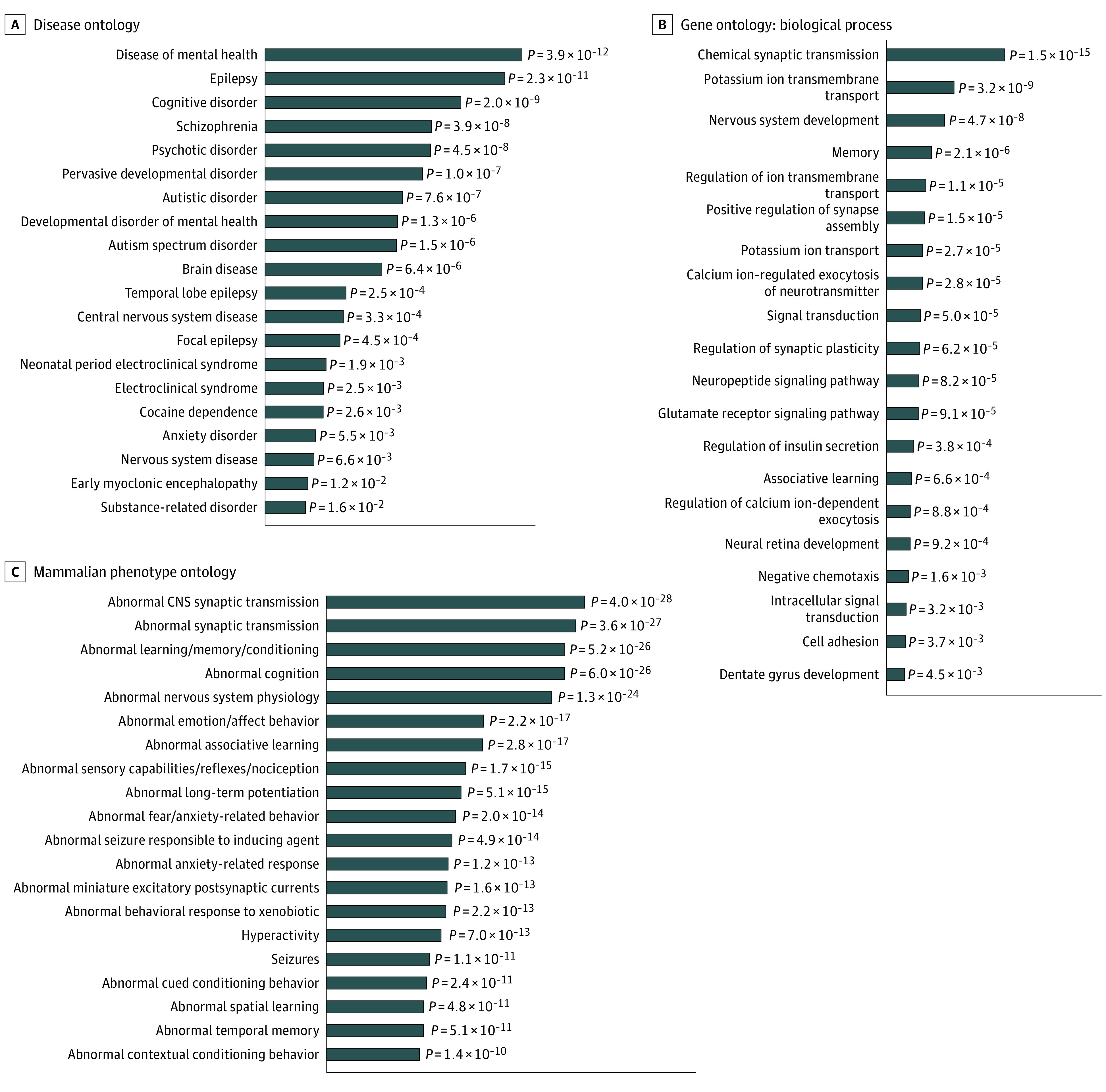
Association of Genes Overexpressed in the Middle Temporal Gyrus (MTG) and Brain Disorders A and B, Genes significantly overexpressed in the MTG were enriched for psychiatric diseases and neurological biological processes in annotations to the disease ontology and biological process domain of the gene ontology, respectively. C, Their mammalian orthologs were enriched for neurological and behavioral phenotypes present in the Mouse Genome Database. For each database, hypergeometric tests were performed comparing the frequency of ontology entity annotations for MTG-expressed genes vs genes available in the Allen Human Brain Atlas datasets. All *P* values are false discovery rate–adjusted. The top 20 most highly enriched results for each ontology are shown.

Genes differentially expressed in each identified ROI were enriched for overrepresentation in the disease ontology (Figure 5A) and the biological process domain of the gene ontology (Figure 5B; eTable 15 in Supplement 1). The MTG contained a highly enriched set of genes involved in neuropsychiatric disorders, specifically schizophrenia (OR, 2.44; 95% CI, 1.87-3.16; *z* score, 7.10; *P* = 5.7 × 10^−7^), psychotic disorder (OR, 2.43; 95% CI, 1.86-3.15; *z* score, 7.06; *P* = 6.5 × 10^−7^), autism spectrum disorder (OR, 3.70; 95% CI, 2.42-5.50; *z* score, 7.01; *P* = 1.1 × 10^−5^), cognitive disorder (OR, 2.37; 95% CI, 1.86-2.99; *z* score, 7.56; *P* = 2.8 × 10^−8^), and epilepsy (OR, 4.66; 95% CI, 3.17-6.71; *z* score, 9.27; *P* = 3.2 × 10^−10^). Enriched biological processes in the AHBA results relate to nervous system development and synaptic transmission, while phenotypes from orthologous mouse models (Figure 5C) exhibit abnormal brain, cognition, anxiety, and affective traits.

## Discussion

Using mendelian randomization in a large population data set, we explored potential causal associations between inflammation and brain structure. In keeping with our hypothesis, this study found that genetically predicted IL-6, but not other inflammatory markers, was significantly associated with GMV and CT. We found the strongest associations with GMV in the MTG and fusiform gyrus and with CT in the superior frontal gyrus. Using the AHBA, we also demonstrated that the MTG significantly overexpressed a highly interconnected number of genes in an interaction network with IL-6 together with a set of overexpressed genes for epilepsy, cognitive disorder, schizophrenia, psychotic disorder, and autism spectrum disorder. These results suggest that function within the innate immune system, and particularly IL-6–related pathways, are essential for normal brain development and that elevation of IL-6 may affect development of brain structure in areas highly implicated in mental health disorders, particularly those with a neurodevelopmental pathway.

It should be noted that IL-6 genetic variants used as instruments in MR are associated with increased circulating IL-6 levels but decreased IL-6 classic signaling owing to reduced expression of membrane-bound IL-6R.^40^ Thus, caution is needed when considering the directionality of association between IL-6 levels and change in brain volumes. However, dysregulation of the inflammatory response can trigger a cascade that affects neuronal development and subsequent downstream behavioral phenotypes.^41^ Our results are suggestive of elevation of IL-6 levels and reduction in GMV, with the largest associations within the MTG, a key area of language, semantic memory processing, and sensory integration implicated in a number of neuropsychiatric disorders.^42,43^ These findings extend recent evidence of association between inflammation and brain structure in schizophrenia and depression^22,44,45,46^ and address uncertainties of smaller samples and confounding.

As a whole, genes differentially expressed in the MTG share unexpectedly frequent occurrences in schizophrenia (Figure 5A) as well as autism spectrum disorder, cognitive dysfunction, and epilepsy, suggesting a role for these genes across comorbid and highly heritable illnesses. Suggested mechanisms from our results include neuropeptide and chemical synaptic transmission disruption (Figure 5B) and neurogenesis/developmental processes. The homologs of differentially expressed genes preferentially affect abnormal synaptic transmission and predicate anxiety, emotion, learning, conditioning, and memory behavior—all hallmarks of phenotypically related neuropsychiatric disorders (Figure 5C).

Recently, evidence has emerged for a reduction in GMV, particularly in the temporal lobe in those at risk of poor outcome in psychosis.^42^ Our results are in keeping with findings from large cohort analysis; for example, Boedhoe et al^47^ identified increased CT in frontal regions in autism spectrum disorder and Jalbrzikowski et al^48^ found reduced GMV in the fusiform, temporal, and paracentral regions in patients who converted from clinical high-risk status to psychosis.

Genome-wide association studies in schizophrenia and autism spectrum disorder have implicated the major histocompatibility complex on chromosome 6, with key loci that code for specific cell-surface proteins essential within the immune system.^49^ Additional variants on genes coding for inflammatory cytokines have also been implicated in schizophrenia risk.^23^ Results presented in our analysis support the potential role of IL-6 with brain structure and potentially related neuropsychiatric disorders.

However, we found no significant associations with CRP, BDNF, IL-1, or IL-2 that survived testing for multiple comparison. Higher levels of CRP have been shown to be associated with risk of psychosis and depression,^50^ although depression has a varied relationship with individual inflammatory markers, while more unified evidence exists in schizophrenia.^6^ It is possible that IL-6 has a specific pathway to increased effect on brain structure and poor functional outcome in subgroups transdiagnostically.^51,52^ A plausible effect of IL-6 in inducing changes in brain structure may be maternal immune activation during development, although it is possible that the exposure continues and may indeed affect brain structure throughout adulthood.^53,54^

In the adult human brain, we found that IL-6 may form an interacting community with other proteins differentially overexpressed in the MTG, including CCK and NPY, which are also implicated in schizophrenia and other mental illnesses (Figure 4C). CCK is one of the most abundantly expressed neurotransmitters in the brain.^51^ In humans, GWAS has found an association of CCK in pathways with increased neurofibrillary tangles and TREM2 protein levels, both previously associated with Alzheimer disease and related tauopathies.^55,56^ Despite mixed success as a direct therapeutic aid to ameliorate anxiety and symptoms of schizophrenia via CCK receptor antagonists,^57^ CCK knockout mice have increased prepulse inhibition,^58^ a key biomarker of the sensory overload characteristic of psychosis,^59^ and NPY mouse models of schizophrenia show increased anxiety traits^60^ and abnormal susceptibility to induced seizures.^61,62^

MET and HSPB3 are also differentially overexpressed in the MTG. Mouse models reveal developmental neurological roles for MET, including impaired learning and cued conditioning behavior,^63,64^ potentially relevant for models of perceptual prior beliefs in psychosis.^65^ These proteins potentially interact with IL-6 directly and join IL-6 to a highly connected functional hub of coexpressed genes in the MTG implicated together in neuropsychiatric disorders. MLIP, which interacts with MET is implicated in 13 schizophrenia trios in a 5.59-kilobase deletion.^66^ In mice, there have been no reported studies investigating MLIP and mental health inflammation-mediated mechanisms^58^; thus, our findings may support future MET/MLIP knockout behavioral assays.

### Strengths and Limitations

Strengths include a novel multistage investigation, well-characterized single-nucleotide variants, large data set, stringent corrections for multiple comparisons, and detailed unrestricted gene expression analysis from postmortem human data with homologues from mice, allowing potential for back translation. This study also has limitations that need to be acknowledged. First, our sample was of neurotypical participants in both UK Biobank and AHBA. Other potential data, eg, the Stanley Medical Research Institute database,^67^ from individuals with mental illness did not have transcriptomics data from the brain subregions significant in our MR analysis. The UK Biobank includes patients with a schizophrenia diagnosis; however, only 15 had relevant brain imaging data. Excluding individuals with psychiatric conditions did not alter our results (eTables 5 and 6 in Supplement 1). Second, our MR analysis used hemisphere-specific (left or right) MRI-derived phenotypes, whereas the AHBA is largely of the left hemisphere only. This creates the assumption that the gene expression in each hemisphere is correlated and ignores functional and structural asymmetry. The degree to which hemispheric differences and the quality of the AHBA data vary between individuals may mitigate this limitation. Third, it is possible that the genetic variants in our MR analysis could be associated with other pathways that influence brain structure directly (ie, environmentally via infection-induced effect on brain structure or otherwise not via the associated biomarker); however, given the known genetic architecture of the inflammatory biomarkers, this may be unlikely. This study used a conservative approach to inferring causal gene-mediated phenotype-phenotype relationships, ensuring robust findings for further investigation. In the MR analysis, we used characterized single-nucleotide variants with known cis-regulatory effects on levels of each inflammatory cytokine studied. However, as with any MR analysis, we have made several assumptions: in these analyses, we have tested the assumption that our instrumental variables are associated with inflammation directly, with the example of circulating CRP, which is available in the UK Biobank. We assume that instrumental variables are independent of potential confounders and that horizontal pleiotropy is not present. Fourth, the AHBA used only 6 participants, a small sample size in transcriptomics. Fifth, the available data in UK Biobank and AHBA are limited to people of European ancestry, and significant replication when diverse samples become available is essential.

## Conclusions

This mendelian randomization study found that IL-6 was associated with changes in brain structure, with associations strongest in the MTG where several genes are differentially overexpressed compared with the whole brain. These genes form a highly connected network at the protein level and functionally contribute to diseases and phenotypes related to schizophrenia, autism spectrum disorder, and epilepsy. This suggests a genetically mediated, tissue-specific neuroinflammatory cascade relevant to brain structure in neuropsychiatric disorders. These findings can be modeled computationally and should be tested further for mechanistic insights in further preclinical models.

## Notes

Supplement 1.
eMethods.

eDiscussion.
**eTable 1.** Demographic variables from the UK Biobank sample with brain imaging measures included in mendelian randomization analysis. **eTable 2.** Genetic variants used in mendelian randomization analyses for inflammatory biomarkers. **eTable 3.** Brain imaging measures associated with genetically predicted levels of interleukin 6 at a suggestive or multiple-corrected level of statistical significance. **eTable 4.** Brain imaging measures associated with genetically predicted levels of other inflammatory biomarkers at a suggestive level of statistical significance. **eTable 5.** Brain imaging measures from eTable 3 associated with genetically predicted levels of interleukin 6, including adjustments for brain size and excluding psychiatric illness. **eTable 6.** Brain imaging measures from eTable 4 associated with genetically predicted levels of other inflammatory biomarkers, including adjustments for brain size and excluding psychiatric illness. **eFigure 1.** Heat map of associations between genetically predicted inflammatory biomarkers and brain imaging measures in subcategory 1101 (regional grey matter). **eFigure 2.** Heat map of associations between genetically predicted inflammatory biomarkers and brain imaging measures in subcategory 190 (Freesurfer ASEG). **eFigure 3.** Heat map of associations between genetically predicted inflammatory biomarkers and brain imaging measures in subcategory 191 (Freesurfer subsegmentation). **eFigure 4.** Heat map of associations between genetically predicted inflammatory biomarkers and brain imaging measures in subcategory 192 (Freesurfer desikan white). **eFigure 5. **Heat map of associations between genetically predicted inflammatory biomarkers and brain imaging measures in subcategory 193 (Freesurfer desikan pial). **eFigure 6.** Heat map of associations between genetically predicted inflammatory biomarkers and brain imaging measures in subcategory 194 (Freesurfer desikan grey-white). **eFigure 7.** Heat map of associations between genetically predicted inflammatory biomarkers and brain imaging measures in subcategory 195 (Freesurfer BA exvivo). **eFigure 8.** Heat map of associations between genetically predicted inflammatory biomarkers and brain imaging measures in subcategory 196 (Freesurfer DKT). **eFigure 9.** Heat map of associations between genetically predicted inflammatory biomarkers and brain imaging measures in subcategory 197 (Freesurfer a2009s). **eFigure 10.** Differential gene expression in fro vs whole brain. **eFigure 11.** Differential gene expression in FuG vs whole brain. **eFigure 12.** Differential gene expression in ITG vs whole brain. **eFigure 13.** Differential gene expression in PCu vs whole brain. **eFigure 14.** Differential gene expression in Pu vs whole brain. **eFigure 15.** Differential gene expression in SFG vs whole brain. **eFigure 16.** Differential gene expression in Vel_IV vs whole brain. **eTable 7.** Differential expression results in the fro. **eTable 8.** Differential expression results in the Fug. **eTable 9.** Differential expression results in the ITG. **eTable 10.** Differential expression results in the MTG. **eTable 11.** Differential expression results in the PCu. **eTable 12.** Differential expression results in the Pu. **eTable 13.** Differential expression results in the SFG. **eTable 14.** Differential expression results in the VeI_IV regions. **eTable 15.** Biomedical ontology gene enrichment results. **eTable 16.** Brain imaging measure z scores.

Supplement 2. PIMS Collaboration.

## References

[1] Kappelmann N, Arloth J, Georgakis MK, . Dissecting the association between inflammation, metabolic dysregulation, and specific depressive symptoms: a genetic correlation and 2-sample mendelian randomization study. JAMA Psychiatry. 2021;78(2):161-170. doi:10.1001/jamapsychiatry.2020.3436 33079133 PMC7577200

[2] Corsi-Zuelli F, Deakin B. Impaired regulatory T cell control of astroglial overdrive and microglial pruning in schizophrenia. Neurosci Biobehav Rev. 2021;125:637-653. doi:10.1016/j.neubiorev.2021.03.004 33713699

[3] Khandaker GM, Zimbron J, Dalman C, Lewis G, Jones PB. Childhood infection and adult schizophrenia: a meta-analysis of population-based studies. Schizophr Res. 2012;139(1-3):161-168. doi:10.1016/j.schres.2012.05.023 22704639 PMC3485564

[4] Khandaker GM, Zimbron J, Lewis G, Jones PB. Prenatal maternal infection, neurodevelopment and adult schizophrenia: a systematic review of population-based studies. Psychol Med. 2013;43(2):239-257. doi:10.1017/S0033291712000736 22717193 PMC3479084

[5] Brown AS, Derkits EJ. Prenatal infection and schizophrenia: a review of epidemiologic and translational studies. Am J Psychiatry. 2010;167(3):261-280. doi:10.1176/appi.ajp.2009.09030361 20123911 PMC3652286

[6] Perry BI, Upthegrove R, Kappelmann N, Jones PB, Burgess S, Khandaker GM. Associations of immunological proteins/traits with schizophrenia, major depression and bipolar disorder: a bi-directional two-sample mendelian randomization study. Brain Behav Immun. 2021;97:176-185. doi:10.1016/j.bbi.2021.07.009 34280516 PMC7612947

[7] Perry BI, Upthegrove R, Thompson A, . Dysglycaemia, inflammation and psychosis: findings from the UK ALSPAC birth cohort. Schizophr Bull. 2019;45(2):330-338. doi:10.1093/schbul/sby040 29635418 PMC6403055

[8] Upthegrove R, Manzanares-Teson N, Barnes NM. Cytokine function in medication-naive first episode psychosis: a systematic review and meta-analysis. Schizophr Res. 2014;155(1-3):101-108. doi:10.1016/j.schres.2014.03.005 24704219

[9] Smith SEP, Li J, Garbett K, Mirnics K, Patterson PH. Maternal immune activation alters fetal brain development through interleukin-6. J Neurosci. 2007;27(40):10695-10702. doi:10.1523/JNEUROSCI.2178-07.2007 17913903 PMC2387067

[10] Meyer U, Feldon J. Epidemiology-driven neurodevelopmental animal models of schizophrenia. Prog Neurobiol. 2010;90(3):285-326. doi:10.1016/j.pneurobio.2009.10.018 19857543

[11] Upthegrove R, Khandaker GM. Cytokines, oxidative stress and cellular markers of inflammation in schizophrenia. Curr Top Behav Neurosci. 2020;44:49-66. doi:10.1007/7854_2018_88 31115797

[12] Nikkheslat N, McLaughlin AP, Hastings C, ; NIMA Consortium. Childhood trauma, HPA axis activity and antidepressant response in patients with depression. Brain Behav Immun. 2020;87:229-237. doi:10.1016/j.bbi.2019.11.024 31794798 PMC7327513

[13] Goldsmith DR, Haroon E, Miller AH, . Association of baseline inflammatory markers and the development of negative symptoms in individuals at clinical high risk for psychosis. Brain Behav Immun. 2019;76:268-274. doi:10.1016/j.bbi.2018.11.315 30496778 PMC6348114

[14] Khandaker G, Meyer U, Jones PB, eds. Neuroinflammation and Schizophrenia. Springer International Publishing; 2020. doi:10.1007/978-3-030-39141-6

[15] Jones HJ, Heron J, Hammerton G, ; 23 and Me Research Team. Investigating the genetic architecture of general and specific psychopathology in adolescence. Transl Psychiatry. 2018;8(1):145. doi:10.1038/s41398-018-0204-9 30089819 PMC6082910

[16] Opel N, Goltermann J, Hermesdorf M, Berger K, Baune BT, Dannlowski U. Cross-disorder analysis of brain structural abnormalities in six major psychiatric disorders: a secondary analysis of mega- and meta-analytical findings from the ENIGMA consortium. Biol Psychiatry. 2020;88(9):678-686. doi:10.1016/j.biopsych.2020.04.027 32646651

[17] Anderson KM, Collins MA, Kong R, . Convergent molecular, cellular, and cortical neuroimaging signatures of major depressive disorder. Proc Natl Acad Sci U S A. 2020;117(40):25138-25149. doi:10.1073/pnas.2008004117 32958675 PMC7547155

[18] Campbell M, Jahanshad N, Mufford M, . Overlap in genetic risk for cross-disorder vulnerability to mental disorders and genetic risk for altered subcortical brain volumes. J Affect Disord. 2021;282:740-756. doi:10.1016/j.jad.2020.12.062 33601715

[19] Bai YM, Chen MH, Hsu JW, . A comparison study of metabolic profiles, immunity, and brain gray matter volumes between patients with bipolar disorder and depressive disorder. J Neuroinflammation. 2020;17(1):42. doi:10.1186/s12974-020-1724-9 32000805 PMC6990475

[20] Birnbaum R, Jaffe AE, Chen Q, ; BrainSeq Consortium. Investigating the neuroimmunogenic architecture of schizophrenia. Mol Psychiatry. 2018;23(5):1251-1260. doi:10.1038/mp.2017.89 28485405

[21] Tu PC, Li CT, Lin WC, Chen MH, Su TP, Bai YM. Structural and functional correlates of serum soluble IL-6 receptor level in patients with bipolar disorder. J Affect Disord. 2017;219:172-177. doi:10.1016/j.jad.2017.04.036 28558364

[22] Deakin B, Suckling J, Barnes TRE, ; BeneMin Study team. The benefit of minocycline on negative symptoms of schizophrenia in patients with recent-onset psychosis (BeneMin): a randomised, double-blind, placebo-controlled trial. Lancet Psychiatry. 2018;5(11):885-894. doi:10.1016/S2215-0366(18)30345-6 30322824 PMC6206257

[23] Birnbaum R, Weinberger DR. A Genetics perspective on the role of the (neuro)immune system in schizophrenia. Schizophr Res. 2020;217:105-113. doi:10.1016/j.schres.2019.02.005 30850283 PMC6728242

[24] Afridi R, Seol S, Kang HJ, Suk K. Brain-immune interactions in neuropsychiatric disorders: lessons from transcriptome studies for molecular targeting. Biochem Pharmacol. 2021;188:114532. doi:10.1016/j.bcp.2021.114532 33773976

[25] MacKenzie G, Subramaniam S, Caldwell LJ, . Research priorities for neuroimmunology: identifying the key research questions to be addressed by 2030. Wellcome Open Res. 2021;6(194):194. doi:10.12688/wellcomeopenres.16997.1 34778569 PMC8558843

[26] Sudlow C, Gallacher J, Allen N, . UK Biobank: an open access resource for identifying the causes of a wide range of complex diseases of middle and old age. PLoS Med. 2015;12(3):e1001779. doi:10.1371/journal.pmed.1001779 25826379 PMC4380465

[27] Interleukin 1 Genetics Consortium. Cardiometabolic effects of genetic upregulation of the interleukin 1 receptor antagonist: a mendelian randomisation analysis. Lancet Diabetes Endocrinol. 2015;3(4):243-253. doi:10.1016/S2213-8587(15)00034-0 25726324 PMC4648058

[28] Ahola-Olli AV, Würtz P, Havulinna AS, . Genome-wide association study identifies 27 loci influencing concentrations of circulating cytokines and growth factors. Am J Hum Genet. 2017;100(1):40-50. doi:10.1016/j.ajhg.2016.11.007 27989323 PMC5223028

[29] Swerdlow DI, Holmes MV, Kuchenbaecker KB, ; Interleukin-6 Receptor Mendelian Randomisation Analysis (IL6R MR) Consortium. The interleukin-6 receptor as a target for prevention of coronary heart disease: a mendelian randomisation analysis. Lancet. 2012;379(9822):1214-1224. doi:10.1016/S0140-6736(12)60110-X 22421340 PMC3316968

[30] Wensley F, Gao P, Burgess S, ; C Reactive Protein Coronary Heart Disease Genetics Collaboration (CCGC). Association between C reactive protein and coronary heart disease: mendelian randomisation analysis based on individual participant data. BMJ. 2011;342:d548. doi:10.1136/bmj.d548 21325005 PMC3039696

[31] Terracciano A, Piras MG, Lobina M, . Genetics of serum BDNF: meta-analysis of the Val66Met and genome-wide association study. World J Biol Psychiatry. 2013;14(8):583-589. doi:10.3109/15622975.2011.616533 22047184 PMC3288597

[32] Miller KL, Alfaro-Almagro F, Bangerter NK, . Multimodal population brain imaging in the UK Biobank prospective epidemiological study. Nat Neurosci. 2016;19(11):1523-1536. doi:10.1038/nn.4393 27643430 PMC5086094

[33] Alfaro-Almagro F, Jenkinson M, Bangerter NK, . Image processing and quality control for the first 10,000 brain imaging datasets from UK Biobank. Neuroimage. 2018;166:400-424. doi:10.1016/j.neuroimage.2017.10.034 29079522 PMC5770339

[34] Sunkin SM, Ng L, Lau C, . Allen Brain Atlas: an integrated spatio-temporal portal for exploring the central nervous system. Nucleic Acids Res. 2013;41(database issue):D996-D1008. doi:10.1093/nar/gks1042 23193282 PMC3531093

[35] Gene Ontology Consortium. Gene Ontology Consortium: going forward. Nucleic Acids Res. 2015;43(database issue):D1049-D1056. doi:10.1093/nar/gku1179 25428369 PMC4383973

[36] Schriml LM, Mitraka E, Munro J, . Human Disease Ontology 2018 update: classification, content and workflow expansion. Nucleic Acids Res. 2019;47(D1):D955-D962. doi:10.1093/nar/gky1032 30407550 PMC6323977

[37] Smith CL, Goldsmith CAW, Eppig JT. The Mammalian Phenotype Ontology as a tool for annotating, analyzing and comparing phenotypic information. Genome Biol. 2005;6(1):R7. doi:10.1186/gb-2004-6-1-r7 15642099 PMC549068

[38] Szklarczyk D, Gable AL, Lyon D, . STRING v11: protein-protein association networks with increased coverage, supporting functional discovery in genome-wide experimental datasets. Nucleic Acids Res. 2019;47(D1):D607-D613. doi:10.1093/nar/gky1131 30476243 PMC6323986

[39] Brett M, Anton JL, Valabregue R, Poline JB. Region of interest analysis using an SPM toolbox. Paper presented at: 8th International Conference on Functional Mapping of the Human Brain; June 2-6, 2002; Sendai, Japan.

[40] Ferreira RC, Freitag DF, Cutler AJ, . Functional IL6R 358Ala allele impairs classical IL-6 receptor signaling and influences risk of diverse inflammatory diseases. PLoS Genet. 2013;9(4):e1003444. doi:10.1371/journal.pgen.1003444 23593036 PMC3617094

[41] Comer AL, Carrier M, Tremblay MÈ, Cruz-Martín A. The inflamed brain in schizophrenia: the convergence of genetic and environmental risk factors that lead to uncontrolled neuroinflammation. Front Cell Neurosci. 2020;14:274. doi:10.3389/fncel.2020.00274 33061891 PMC7518314

[42] Merritt K, Luque Laguna P, Irfan A, David AS. Longitudinal structural MRI findings in individuals at genetic and clinical high risk for psychosis: a systematic review. Front Psychiatry. 2021;12:620401. doi:10.3389/fpsyt.2021.620401 33603688 PMC7884337

[43] Vucurovic K, Caillies S, Kaladjian A. Neural correlates of mentalizing in individuals with clinical high risk for schizophrenia: ALE meta-analysis. Front Psychiatry. 2021;12:634015. doi:10.3389/fpsyt.2021.634015 33959048 PMC8095711

[44] Wang AK, Miller BJ. Meta-analysis of cerebrospinal fluid cytokine and tryptophan catabolite alterations in psychiatric patients: comparisons between schizophrenia, bipolar disorder, and depression. Schizophr Bull. 2018;44(1):75-83. doi:10.1093/schbul/sbx035 28338954 PMC5768046

[45] Laskaris L, Mancuso S, Shannon Weickert C, . Brain morphology is differentially impacted by peripheral cytokines in schizophrenia-spectrum disorder. Brain Behav Immun. 2021;95:299-309. doi:10.1016/j.bbi.2021.04.002 33838248

[46] Chen MH, Kao ZK, Chang WC, . Increased proinflammatory cytokines, executive dysfunction, and reduced gray matter volumes in first-episode bipolar disorder and major depressive disorder. J Affect Disord. 2020;274:825-831. doi:10.1016/j.jad.2020.05.158 32664021

[47] Boedhoe PSW, van Rooij D, Hoogman M, ; ENIGMA ADHD Working Group; ENIGMA ASD Working Group; ENIGMA OCD Working Group. Subcortical brain volume, regional cortical thickness, and cortical surface area across disorders: findings from the ENIGMA ADHD, ASD, and OCD working groups. Am J Psychiatry. 2020;177(9):834-843. doi:10.1176/appi.ajp.2020.19030331 32539527 PMC8296070

[48] Jalbrzikowski M, Hayes RA, Wood SJ, ; ENIGMA Clinical High Risk for Psychosis Working Group. Association of structural magnetic resonance imaging measures with psychosis onset in individuals at clinical high risk for developing psychosis: an ENIGMA working group mega-analysis. JAMA Psychiatry. 2021;78(7):753-766. doi:10.1001/jamapsychiatry.2021.0638 33950164 PMC8100913

[49] Purcell SM, Wray NR, Stone JL, ; International Schizophrenia Consortium. Common polygenic variation contributes to risk of schizophrenia and bipolar disorder. Nature. 2009;460(7256):748-752. doi:10.1038/nature08185 19571811 PMC3912837

[50] Khandaker GM, Pearson RM, Zammit S, Lewis G, Jones PB. Association of serum interleukin 6 and C-reactive protein in childhood with depression and psychosis in young adult life: a population-based longitudinal study. JAMA Psychiatry. 2014;71(10):1121-1128. doi:10.1001/jamapsychiatry.2014.1332 25133871 PMC4561502

[51] Lonsdale J, Thomas J, Salvatore M, . The Genotype-Tissue Expression (GTEx) project. Nat Genet. 2013;45(6):580-585. doi:10.1038/ng.2653 23715323 PMC4010069

[52] Miller JA, Ding SL, Sunkin SM, . Transcriptional landscape of the prenatal human brain. Nature. 2014;508(7495):199-206. doi:10.1038/nature13185 24695229 PMC4105188

[53] Wu WL, Hsiao EY, Yan Z, Mazmanian SK, Patterson PH. The placental interleukin-6 signaling controls fetal brain development and behavior. Brain Behav Immun. 2017;62:11-23. doi:10.1016/j.bbi.2016.11.007 27838335 PMC5373986

[54] Purves-Tyson TD, Weber-Stadlbauer U, Richetto J, . Increased levels of midbrain immune-related transcripts in schizophrenia and in murine offspring after maternal immune activation. Mol Psychiatry. 2021;26(3):849-863. doi:10.1038/s41380-019-0434-0 31168068 PMC7910216

[55] Liu C, Yu J. Genome-wide association studies for cerebrospinal fluid soluble TREM2 in Alzheimer’s disease. Front Aging Neurosci. 2019;11:297. doi:10.3389/fnagi.2019.00297 31708768 PMC6823606

[56] Beecham GW, Hamilton K, Naj AC, ; Alzheimer’s Disease Genetics Consortium (ADGC). Genome-wide association meta-analysis of neuropathologic features of Alzheimer’s disease and related dementias. PLoS Genet. 2014;10(9):e1004606. doi:10.1371/journal.pgen.1004606 25188341 PMC4154667

[57] Ballaz SJ, Bourin M. Cholecystokinin-mediated neuromodulation of anxiety and schizophrenia: a “dimmer-switch” hypothesis. Curr Neuropharmacol. 2021;19(7):925-938. doi:10.2174/1570159X18666201113145143 33185164 PMC8686311

[58] Bult CJ, Blake JA, Smith CL, Kadin JA, Richardson JE; Mouse Genome Database Group. Mouse Genome Database (MGD) 2019. Nucleic Acids Res. 2019;47(D1):D801-D806. doi:10.1093/nar/gky1056 30407599 PMC6323923

[59] Mena A, Ruiz-Salas JC, Puentes A, Dorado I, Ruiz-Veguilla M, De la Casa LG. Reduced prepulse inhibition as a biomarker of schizophrenia. Front Behav Neurosci. 2016;10:202. doi:10.3389/fnbeh.2016.00202 27803654 PMC5067522

[60] Karl T, Duffy L, Herzog H. Behavioural profile of a new mouse model for NPY deficiency. Eur J Neurosci. 2008;28(1):173-180. doi:10.1111/j.1460-9568.2008.06306.x 18616565

[61] Ste Marie L, Luquet S, Cole TB, Palmiter RD. Modulation of neuropeptide Y expression in adult mice does not affect feeding. Proc Natl Acad Sci U S A. 2005;102(51):18632-18637. doi:10.1073/pnas.0509240102 16339312 PMC1309050

[62] Erickson JC, Clegg KE, Palmiter RD. Sensitivity to leptin and susceptibility to seizures of mice lacking neuropeptide Y. Nature. 1996;381(6581):415-421. doi:10.1038/381415a0 8632796

[63] Martins GJ, Shahrokh M, Powell EM. Genetic disruption of Met signaling impairs GABAergic striatal development and cognition. Neuroscience. 2011;176:199-209. doi:10.1016/j.neuroscience.2010.12.058 21195751 PMC3040282

[64] Ieraci A, Forni PE, Ponzetto C. Viable hypomorphic signaling mutant of the Met receptor reveals a role for hepatocyte growth factor in postnatal cerebellar development. Proc Natl Acad Sci U S A. 2002;99(23):15200-15205. doi:10.1073/pnas.222362099 12397180 PMC137567

[65] Powers AR, Mathys C, Corlett PR. Pavlovian conditioning-induced hallucinations result from overweighting of perceptual priors. Science. 2017;357(6351):596-600. doi:10.1126/science.aan3458 28798131 PMC5802347

[66] Wu X, Huai C, Shen L, . Genome-wide study of copy number variation implicates multiple novel loci for schizophrenia risk in Han Chinese family trios. iScience. 2021;24(8):102894. doi:10.1016/j.isci.2021.102894 34401673 PMC8358640

[67] Kim S, Webster MJ. The Stanley Neuropathology Consortium Integrative Database: a novel, web-based tool for exploring neuropathological markers in psychiatric disorders and the biological processes associated with abnormalities of those markers. Neuropsychopharmacology. 2010;35(2):473-482. doi:10.1038/npp.2009.151 19829293 PMC3055386

