# Supplementary material for: Inflammation and Brain Structure in Schizophrenia and Other Neuropsychiatric Disorders: A Mendelian Randomization Study
Source: JAMA Psychiatry. 2022 Mar 30;79(5):498–507. doi: 10.1001/jamapsychiatry.2022.0407 (PMC8968718; doi:10.1001/jamapsychiatry.2022.0407)
Supplement: Supplement 1. — eMethods. eDiscussion. eTable 1. Demographic variables from the UK Biobank sample with brain imaging measures included in mendelian randomization analysis. eTable 2. Genetic variants used in mendelian randomization analyses for inflammatory biomarkers. eTable 3. Brain imaging measures associated with genetically predicted levels of interleukin 6 at a suggestive or multiple-corrected level of statistical significance. eTable 4. Brain imaging measures associated with genetically predicted levels of other inflammatory biomarkers at a suggestive level of statistical significance. eTable 5. Brain imaging measures from eTable 3 associated with genetically predicted levels of interleukin 6, including adjustments for brain size and excluding psychiatric illness. eTable 6. Brain imaging measures from eTable 4 associated with genetically predicted levels of other inflammatory biomarkers, including adjustments for brain size and excluding psychiatric illness. eFigure 1. Heat map of associations between genetically predicted inflammatory biomarkers and brain imaging measures in subcategory 1101 (regional grey matter). eFigure 2. Heat map of associations between genetically predicted inflammatory biomarkers and brain imaging measures in subcategory 190 (Freesurfer ASEG). eFigure 3. Heat map of associations between genetically predicted inflammatory biomarkers and brain imaging measures in subcategory 191 (Freesurfer subsegmentation). eFigure 4. Heat map of associations between genetically predicted inflammatory biomarkers and brain imaging measures in subcategory 192 (Freesurfer desikan white). eFigure 5. Heat map of associations between genetically predicted inflammatory biomarkers and brain imaging measures in subcategory 193 (Freesurfer desikan pial). eFigure 6. Heat map of associations between genetically predicted inflammatory biomarkers and brain imaging measures in subcategory 194 (Freesurfer desikan grey-white). eFigure 7. Heat map of associations between genetically pre [file jamapsychiatry-e220407-s001.pdf]

Supplemental Online Content

Williams JA, Burgess S, Suckling J, et al; PIMS Collaboration. Inflammation and brain structure in schizophrenia and other neuropsychiatric disorders: a mendelian randomization study. *JAMA Psychiatry*. Published online March 30, 2022. doi:10.1001/jamapsychiatry.2022.0407

eMethods.

eDiscussion.

eTable 1. Demographic variables from the UK Biobank sample with brain imaging measures included in mendelian randomization analysis

eTable 2. Genetic variants used in mendelian randomization analyses for inflammatory biomarkers

eTable 3. Brain imaging measures associated with genetically predicted levels of interleukin 6 at a suggestive or multiple-corrected level of statistical significance

eTable 4. Brain imaging measures associated with genetically predicted levels of other inflammatory biomarkers at a suggestive level of statistical significance

eTable 5. Brain imaging measures from eTable 3 associated with genetically predicted levels of interleukin 6, including adjustments for brain size and excluding psychiatric illness

eTable 6. Brain imaging measures from eTable 4 associated with genetically predicted levels of other inflammatory biomarkers, including adjustments for brain size and excluding psychiatric illness

eFigure 1. Heat map of associations between genetically predicted inflammatory biomarkers and brain imaging measures in subcategory 1101 (regional grey matter)

eFigure 2. Heat map of associations between genetically predicted inflammatory biomarkers and brain imaging measures in subcategory 190 (Freesurfer ASEG)

eFigure 3. Heat map of associations between genetically predicted inflammatory biomarkers and brain imaging measures in subcategory 191 (Freesurfer subsegmentation)

eFigure 4. Heat map of associations between genetically predicted inflammatory biomarkers and brain imaging measures in subcategory 192 (Freesurfer desikan white)

eFigure 5. Heat map of associations between genetically predicted inflammatory biomarkers and brain imaging measures in subcategory 193 (Freesurfer desikan pial)

eFigure 6. Heat map of associations between genetically predicted inflammatory biomarkers and brain imaging measures in subcategory 194 (Freesurfer desikan grey-white)

© 2022 Williams JA et al. *JAMA Psychiatry*.

eFigure 7. Heat map of associations between genetically predicted inflammatory biomarkers and brain imaging measures in subcategory 195 (Freesurfer BA exvivo)

eFigure 8. Heat map of associations between genetically predicted inflammatory biomarkers and brain imaging measures in subcategory 196 (Freesurfer DKT)

eFigure 9. Heat map of associations between genetically predicted inflammatory biomarkers and brain imaging measures in subcategory 197 (Freesurfer a2009s)

eFigure 10. Differential gene expression in fro vs whole brain

eFigure 11. Differential gene expression in FuG vs whole brain

eFigure 12. Differential gene expression in ITG vs whole brain

eFigure 13. Differential gene expression in PCu vs whole brain

eFigure 14. Differential gene expression in Pu vs whole brain

eFigure 15. Differential gene expression in SFG vs whole brain

eFigure 16. Differential gene expression in VeI_IV vs whole brain

eTable 7. Differential expression results in the fro

eTable 8. Differential expression results in the Fug

eTable 9. Differential expression results in the ITG

eTable 10. Differential expression results in the MTG

eTable 11. Differential expression results in the PCu

eTable 12. Differential expression results in the Pu

eTable 13. Differential expression results in the SFG

eTable 14. Differential expression results in the VeI_IV regions

eTable 15. Biomedical ontology gene enrichment results

eTable 16. Brain imaging measure *z* scores

This supplementary material has been provided by the authors to give readers additional information about their work.

eMethods.

1. **Data:**

Data were taken from the UK Biobank, population-based bioresource containing data on around 502,229 UK residents who were aged between 40 and 69 years at study baseline and recruited between March 2006 and December 2010 from 23 centres in the United Kingdom. This study utilised UK Biobank collected genetic, brain imaging and demographic data. The detailed description of UK Biobank recruitment, ethical approval and data has previously been published[1].

2. **Exposure**

We investigate five exposures that have previously been shown to be associated with risk of mental health disorders in Mendelian randomization investigations: interleukin-1 (IL1), interleukin-2 (IL2), IL6, C-reactive protein (CRP), and brain-derived neurotrophic factor (BDNF) with data available in UKBiobank, in order to examine any specificity within the IL6/IL6r pathway. Exposures were selected due to their potential causality in inflammatory-state related to neuropsychiatric disorders ranging from depression to schizophrenia[2].

For each inflammatory biomarker, we selected genetic variants in a relevant coding gene region previously shown to be conditionally associated with the inflammatory biomarker and only moderately correlated (r^2<0.6).

For IL1, we selected 2 variants in the *IL1RN* gene region (rs6743376 and rs1542176)[3] .

For IL2, we selected 1 variant in the *IL2RA* gene region (rs12722497)[4] .

For IL6, we selected 3 variants in the *IL6R* gene region (rs7529229, rs4845371, and rs12740969)[5] .

For CRP, we selected 4 variants in the *CRP* gene region (rs1205, rs3093077, rs1130864, and rs1800947)[6]. To validate the measure of association with these SNPs and CRP in the outcome sample, we regressed log(CRP) on each SNP. F-statistics for these associations were rs1205 = 292.95, rs3093077 = 105.69, rs1130864 = 176.40, and rs1800947 = 143.88).

For BDNF, we selected 1 variant in the *BDNF* gene region (rs11030102)[7] .

3. **Outcomes**

Genetic associations with brain imaging measures

We considered 1436 brain imaging measures derived from magnetic resonance imaging (MRI) from the imaging subset of the UK Biobank study. Brain imaging data were available on 20,688 individuals of self-reported White ethnicity. We considered all measures reported by UK Biobank from T1-weighted imaging listed under the phenotype code category 110, including multiple measures of correlated segmentations using different levels of resolution. These included multiple atlases; eg DKT and Destrieux, thus we were able to interrogate potential associations with broad regions in DKT and a more fine grained parcellation that is available in Destrieux. Image derived phenotype generation was previously described[8,9]. For a detailed treatment of UK Biobank imaging methods, please see the white paper UK Biobank Brain Imaging Documentation version 1.8 (https://biobank.ctsu.ox.ac.uk/crystal/crystal/docs/brain_mri.pdf)

© 2022 Williams JA et al. *JAMA Psychiatry*.

Genetic associations with brain imaging measures were obtained from linear regression. We first corrected for covariates: age at survey, age-squared, sex, body mass index, 10 genetic principal components, plus technical covariates (measures of head motion and scanner position) by regressing each imaging measure in turn on these covariates and taking the residual value. We then inverse-normal rank transformed these residuals to minimize the influence of outlying values, before regression on each genetic variant in turn to obtain the summarized genetic associations. This analysis was repeated while correcting for whole brain volume, and additionally while excluding patients with psychiatric illnesses (Supplemental Tables 5 and 6, respectively).

4. **Statistical analyses:**

We performed two-sample Mendelian randomization analyses taking genetic associations with the inflammatory biomarkers from published studies (Supplementary Table 2). For inflammatory biomarkers with multiple associated genetic variants, we implemented the inverse-variance weighted method with adjustment for correlation between the variants using the Mendelian randomization package in the R statistical computing environment[10,11]. The correlation matrix was estimated using participants from the 1000 Genomes project identified therein as having European ancestry (corresponding to White in the UK Biobank). This method combines the genetic associations with the outcome into a weighted average association scaled by the genetic association with the biomarker measure, incorporating the genetic correlation into the variance-covariance matrices in a generalized linear model. For inflammatory biomarkers with a single associated genetic variant, we assess the per allele genetic association with the outcome. Estimates are reported as Z-scores, where a positive Z-score represents that genetically-predicted levels of the biomarker were positively associated with the brain imaging measure.

Strict correction for multiple testing would require correction for 1436 outcomes, resulting in a two-sided p-value threshold of 3.5×10^{-5} (corresponding to a Z-score of ±4.14). However, several of the brain measures are related, either because the same quantity is measured in two different ways, or related quantities are measured, such as volume of homotopic brain regions in the left and right hemispheres. Principal components analysis indicated that 95% of the variance in the brain imaging measures was explained by 442 principal components, resulting in a multiply-corrected p-value threshold of $0.05/442 = 1.1 \times 10^{-4}$ (corresponding to a Z-score of ±3.86). We additionally report results at $p<0.001$ (corresponding to a Z-score of ±3.29) as “suggestive evidence” of an association.

5. **Spatial Mapping**

To aid visualization and clarity of areas associated with genetically determined measures of inflammation, given the UK Biobank neuroimaging measures include derived variables from overlapping regions, cortical thickness, and brain volume measures, we mapped significant results from Mendelian randomization using the Harvard-Oxford cortical and subcortical Atlas[12] and the probabilistic atlas of the human cerebellum[13]. Results were imported into the MarsBar toolbox[14] in order to create regions of interest (ROIs) in SPM space. This allowed the visualization of brain regions that were statistically significantly associated with genetically-

© 2022 Williams JA et al. *JAMA Psychiatry*.

predicted levels of biomarkers. Once the ROIs were created with MarsBar, they were converted to cluster images and visualized with the xjview SPM toolbox (https://www.alivelearn.net/xjview).

6. Differential gene expression analyses

We analysed gene expression from the Allen Human Brain Atlas (AHBA)[15], a collection of six post-mortem microarray sets, mapped to ROI's indicated by our Mendelian randomization analysis: specifically the Middle Temporal Gyrus (MTG), Inferior Temporal Gyrus (ITG), Fusiform Gyrus (FuG), Operculum (Fro), Putamen (Pu) and Cerebellum (VeI IV), together with measures of Cortical Thickness in the Precuneus (PCu), Superior Frontal (SFG) additionally to brain regions in the AHBA ontology; see Results section and Figure 3. From the six neurotypical individuals in the AHBA (5 male, 1 female, mean age of 42), four had samples available in the left hemisphere, while two had their left and right hemispheres studied. Data from both hemispheres were averaged when available. Pre-normalized gene expression data were downloaded from https://human.brain-map.org.

Data were combined across hemispheres in each brain before filtering probes. In each sample, we removed probes without pre-annotated ENTREZ IDs and all probes containing an "absence" flag indicating < 1% expression against background intensities. Probes pre-annotated to one gene were retained, and the probe which had highest mean intensity across all regions was selected for genes annotated with many probes using the WGCNA package[16] . For each structure of interest, the Allen Human Brain Atlas ontology version 2 was interrogated. For each of the fro, FuG, ITG, MTG, PCu, OP, SFG, pos, cas, and Ve (I-IV) regions, any ontology region whose ancestor included the ROI (including the region itself) was retained as the experimental group. Data from the fro, FuG, ITG, MTG, PCu, Pu, SFG, and VeI_IV and descendants contained annotated probes in each of the six brains, resulting in eight experiments. Control probes for each experiment were selected from those probes not mapped to the experimental region of interest. Multiple probe values in each region (experiment or the remaining brain regions) were averaged, then fit to a differential expression model using an empirical Bayes framework[17]. Gene expression values were explored by creating a heatmap of expression values in each gene and hierarchically clustering both brain regions and genes with complete linkage via Euclidean distance with pheatmap[18]. Genes were considered differentially expressed within each experiment if they had a false discovery rate (FDR) of < 0.05 and an absolute log-fold change of > 2.

7. Post-differential functional analysis

Genes differentially expressed in each experiment were enriched for over-representation in the Gene Ontology's biological process domain[19], and the Disease Ontology[20]. Mouse orthologs of each over-expressed gene set were also obtained from Ensembl v100[21] and enriched with the Mammalian Phenotype ontology[22]. Gene set enrichment was performed with XGR[23] using all genes expressed in any region in the experiments as background for hypergeometric tests adjusted with Bonferroni correction using an adjusted p-value cut-off of $p < 0.05$, and enforcing the True Path Rule while propagating annotations and p-values. All expression experiments were carried out using R version 4.1.0, and plots were created with ggplot2[24]. 47 differentially over-expressed genes in the MTG region were uploaded to the STRING database v11.0[25,26]. To

© 2022 Williams JA et al. *JAMA Psychiatry*.

investigate the potential of IL6 to preferentially interact with any of these proteins co-expressed in the MTG, IL6 was added to the predicted protein-protein interaction graph produced.

© 2022 Williams JA et al. *JAMA Psychiatry*.

eDiscussion.

This study used a conservative approach to inferring causal gene-mediated phenotype/phenotype relationships, ensuring robust findings for further investigation. In the Mendelian randomization analysis, we used characterized SNPs with known *cis*-regulatory effects on levels of each inflammatory cytokine studied. As with any MR analysis, we have made several assumptions: in this analyses we have tested the assumption that our instrumental variables are associated with inflammation directly, with the example of circulating CRP, which is available in the UK Biobank. We assume that instrumental variables are independent of potential confounders, and that horizontal pleiotropy is not present. PCA-guided Bonferroni correction allowed only large effect sizes to be included for downstream analysis. We used the AHBA to investigate gene expression among specific brain regions, which included standard protocols to correct for batch and brain hemisphere. Additionally, the AHBA samples are all registered to a common atlas to ensure identical semantic segmentation of brain regions between samples. This is in contrast to an approach querying publicly available RNA-Sequencing studies, which would necessitate correcting for conditions and batch effects between labs and RNA acquisition protocols and leave open questions about sample extraction and cross-region sample contamination. The functional analysis of gene sets in the incorporates data from homologous genes in mouse whose traits phenocopy onto human mental illness profiles, suggestive of conserved function among genes studied and relevance to both basic science and between various clinical populations.

This study leverages multiomics, two cohort data, and relies on robust inference from both human and mouse studies, generating hypotheses which may be testable in mouse models of mental illness. As RNA-Sequencing and additional imaging become available in the UK Biobank, additional analyses will be possible combining eQTL analyses and Mendelian randomization to investigate the causal role of IL6-induced changes to gene expression on GMV-derived phenotypes relevant to mental illness and the functional relationship between IL6 and any GMV-related eQTLs active in the MTG and additional brain regions.

There exists ample room for the discovery of inflammation-mediated neuropsychiatric functions of genes highlighted in this study. In advanced aging and Alzheimer's patients, HSBP3 was suggested to epistatically interact with PCAT19 to increase PHF-tau (encoded by MAPT) concentrations[27]. Additionally, MLIP interacts with HSBP3, and has likely under-researched implications for schizophrenia and other mental health disorders. Although most studied in cardiac disorders, it has suggested roles mediating a pathway between childhood mistreatment to depression and post-traumatic stress disorder[28]. Many have not been studied in high-throughput mouse knockout studies, revealing ample room for mechanistic verification[29] .

Supplementary References

1. Sudlow C, Gallacher J, Allen N, et al. UK Biobank: An Open Access Resource for Identifying the Causes of a Wide Range of Complex Diseases of Middle and Old Age. *PLoS Med*. 2015;12(3). doi:10.1371/journal.pmed.1001779

2. Perry BI, Upthegrove R, Kappelmann N, Jones PB, Burgess S, Khandaker GM. Associations of immunological proteins/traits with schizophrenia, major depression and bipolar disorder: A bi-directional two-sample mendelian randomization study. *Brain, Behavior, and Immunity*. Published online July 16, 2021. doi:10.1016/j.bbi.2021.07.009

© 2022 Williams JA et al. *JAMA Psychiatry*.

3. Interleukin 1 Genetics Consortium. Cardiometabolic effects of genetic upregulation of the interleukin 1 receptor antagonist: a Mendelian randomisation analysis. *Lancet Diabetes Endocrinol*. 2015;3(4):243-253. doi:10.1016/S2213-8587(15)00034-0

4. Ahola-Olli AV, Würtz P, Havulinna AS, et al. Genome-wide Association Study Identifies 27 Loci Influencing Concentrations of Circulating Cytokines and Growth Factors. *Am J Hum Genet*. 2017;100(1):40-50. doi:10.1016/j.ajhg.2016.11.007

5. Interleukin-6 Receptor Mendelian Randomisation Analysis (IL6R MR) Consortium, Swerdlow DI, Holmes MV, et al. The interleukin-6 receptor as a target for prevention of coronary heart disease: a mendelian randomisation analysis. *Lancet*. 2012;379(9822):1214-1224. doi:10.1016/S0140-6736(12)60110-X

6. C Reactive Protein Coronary Heart Disease Genetics Collaboration (CCGC), Wensley F, Gao P, et al. Association between C reactive protein and coronary heart disease: mendelian randomisation analysis based on individual participant data. *BMJ*. 2011;342:d548. doi:10.1136/bmj.d548

7. Terracciano A, Piras MG, Lobina M, et al. Genetics of serum BDNF: meta-analysis of the Val66Met and genome-wide association study. *World J Biol Psychiatry*. 2013;14(8):583-589. doi:10.3109/15622975.2011.616533

8. Miller KL, Alfaro-Almagro F, Bangerter NK, et al. Multimodal population brain imaging in the UK Biobank prospective epidemiological study. *Nat Neurosci*. 2016;19(11):1523-1536. doi:10.1038/nn.4393

9. Alfaro-Almagro F, Jenkinson M, Bangerter NK, et al. Image processing and Quality Control for the first 10,000 brain imaging datasets from UK Biobank. *NeuroImage*. 2018;166:400-424. doi:10.1016/j.neuroimage.2017.10.034

10. Yavorska OO, Burgess S. MendelianRandomization: an R package for performing Mendelian randomization analyses using summarized data. *International Journal of Epidemiology*. 2017;46(6):1734-1739. doi:10.1093/ije/dyx034

11. Burgess S, Scott RA, Timpson NJ, Davey Smith G, Thompson SG. Using published data in Mendelian randomization: a blueprint for efficient identification of causal risk factors. *Eur J Epidemiol*. 2015;30(7):543-552. doi:10.1007/s10654-015-0011-z

12. Desikan RS, Ségonne F, Fischl B, et al. An automated labeling system for subdividing the human cerebral cortex on MRI scans into gyral based regions of interest. *Neuroimage*. 2006;31(3):968-980. doi:10.1016/j.neuroimage.2006.01.021

13. Diedrichsen J, Balsters JH, Flavell J, Cussans E, Ramnani N. A probabilistic MR atlas of the human cerebellum. *Neuroimage*. 2009;46(1):39-46. doi:10.1016/j.neuroimage.2009.01.045

14. Brett M, Anton JL, Valabregue R, Poline JB. Region of interest analysis using an SPM toolbox [abstract]. In: Vol 16. NeuroImage; 2002:2.

15. Sunkin SM, Ng L, Lau C, et al. Allen Brain Atlas: an integrated spatio-temporal portal for exploring the central nervous system. *Nucleic Acids Res*. 2013;41(Database issue):D996-D1008. doi:10.1093/nar/gks1042

© 2022 Williams JA et al. *JAMA Psychiatry*.

16. Langfelder P, Horvath S. WGCNA: an R package for weighted correlation network analysis. *BMC Bioinformatics*. 2008;9:559. doi:10.1186/1471-2105-9-559

17. Ritchie ME, Phipson B, Wu D, et al. limma powers differential expression analyses for RNA-sequencing and microarray studies. *Nucleic Acids Res*. 2015;43(7):e47-e47. doi:10.1093/nar/gkv007

18. Kolde R. *Pheatmap: Pretty Heatmaps*.; 2018. https://CRAN.R-project.org/package=pheatmap

19. Gene Ontology Consortium. Gene Ontology Consortium: going forward. *Nucleic Acids Res*. 2015;43(Database issue):D1049-1056. doi:10.1093/nar/gku1179

20. Schriml LM, Arze C, Nadendla S, et al. Disease Ontology: a backbone for disease semantic integration. *Nucleic Acids Res*. 2012;40(Database issue):D940-946. doi:10.1093/nar/gkr972

21. Rigden DJ, Fernández XM. The 2018 Nucleic Acids Research database issue and the online molecular biology database collection. *Nucleic Acids Res*. 2018;46(D1):D1-D7. doi:10.1093/nar/gkx1235

22. Smith CL, Eppig JT. The Mammalian Phenotype Ontology: enabling robust annotation and comparative analysis. *Wiley Interdiscip Rev Syst Biol Med*. 2009;1(3):390-399. doi:10.1002/wsbm.44

23. Fang H, Knezevic B, Burnham KL, Knight JC. XGR software for enhanced interpretation of genomic summary data, illustrated by application to immunological traits. *Genome Medicine*. 2016;8(1):129. doi:10.1186/s13073-016-0384-y

24. Wickham H. *Ggplot2: Elegant Graphics for Data Analysis*. Springer-Verlag New York; 2016. http://ggplot2.org

25. Szklarczyk D, Gable AL, Lyon D, et al. STRING v11: protein-protein association networks with increased coverage, supporting functional discovery in genome-wide experimental datasets. *Nucleic Acids Res*. 2019;47(D1):D607-D613. doi:10.1093/nar/gky1131

26. von Mering C, Jensen LJ, Snel B, et al. STRING: known and predicted protein-protein associations, integrated and transferred across organisms. *Nucleic Acids Res*. 2005;33(Database issue):D433-437. doi:10.1093/nar/gki005

27. Wang H, Yang J, Schneider JA, De Jager PL, Bennett DA, Zhang HY. Genome-wide interaction analysis of pathological hallmarks in Alzheimer’s disease. *Neurobiol Aging*. 2020;93:61-68. doi:10.1016/j.neurobiolaging.2020.04.025

28. Fang R, Yang H, Gao Y, Cao H, Goode EL, Cui Y. Gene-based mediation analysis in epigenetic studies. *Briefings in Bioinformatics*. 2021;22(3). doi:10.1093/bib/bbaa113

29. Dickinson ME, Flenniken AM, Ji X, et al. High-throughput discovery of novel developmental phenotypes. *Nature*. 2016;537(7621):508-514. doi:10.1038/nature19356

© 2022 Williams JA et al. *JAMA Psychiatry*.

eTable 1. Demographic variables from the UK Biobank sample with brain imaging measures included in mendelian randomization analysis

Variable	Response	N	Mean, Median, SD	Age D	CRP D
Age			54.93, 56, 7.49		
CRP			2.09, 1.09, 3.60		
Gender	Female	10,828		0.18	0.04
	Male	9,860			
Diagnosis	Autism	11		0.18	0.29
	Bipolar	53		0.11	0.07
	Depression	137		0.14	0.11
	Schizophrenia	15		0.10	0.02
Neurotypical		20,488		0.14	0.09

Diagnoses were taken from Biobank category 1712: Recurrent Depressive Disorder (ICD-10 F33), Schizophrenia (ICD-10 F20), Bipolar Disorder (ICD-10 F31) and Autism (Pervasive developmental disorder from ICD-10 F84). To avoid large numbers artificially inflating p-values from t-tests, the absolute standardized mean difference (Cohen's D) was calculated for Age and c-reactive protein (CRP) for gender, diagnoses of autism, bipolar disorder, depression, schizophrenia, and lack of diagnoses (assumed neurotypical). All differences in age and CRP are "small" or "very small," see Cohen, J. (1988). Statistical power analysis for the behavioral sciences (2nd Ed.). New York: Routledge. N = total, SD = standard deviation.

© 2022 Williams JA et al. *JAMA Psychiatry*.

eTable 2. Genetic variants used in mendelian randomization analyses for inflammatory biomarkers

* Effect alleles were updated to the forward strand, and hence in some cases differ from that reported by the original publication.

Biomarker	rsid	Effect allele *	Association with biomarker	Source
IL-1	rs6743376	C	0.25 SD increase in IL-1Ra	Freitag, 2015 [1]
IL-1	rs1542176	C	0.18 SD increase in IL-1Ra	Freitag, 2015 [1]
IL-2	rs12722497	C	0.63 SD increase in IL-2Ra	Ahola-Olli, 2017 [2]
IL-6	rs7529229	C	0.086 unit increase in log-transformed IL-6	IL6R MR Consortium, 2012 [3]
IL-6	rs4845371	C	0.062 unit increase in log-transformed IL-6	IL6R MR Consortium, 2012 [3]
IL-6	rs12740969	G	0.078 unit increase in log-transformed IL-6	IL6R MR Consortium, 2012 [3]
CRP	rs1205	C	0.169 SD increase in log-transformed CRP	Wensley, 2011 [4]
CRP	rs3093077	C	0.207 SD increase in log-transformed CRP	Wensley, 2011 [4]
CRP	rs1130864	A	0.127 SD increase in log-transformed CRP	Wensley, 2011 [4]
CRP	rs1800947	C	0.232 SD increase in log-transformed CRP	Wensley, 2011 [4]
BDNF	rs11030102	C	0.66 SD increase in BDNF	Terracciano, 2013 [5]

© 2022 Williams JA et al. *JAMA Psychiatry*.

eTable 3. Brain imaging measures associated with genetically predicted levels of interleukin 6 at a suggestive or multiple-corrected level of statistical significance

Trait code	Trait name	Trait category	Z-score	Significant?
27071	Area of perirhinal (left hemisphere)	Freesurfer BA exvivo	5.88	✓
25807	Volume of grey matter in Middle Temporal Gyrus, temporooccipital part (right)	Regional grey matter	5.76	✓
27399	Area of S-subparietal (left hemisphere)	Freesurfer a2009s	5.13	✓
27100	Volume of entorhinal (left hemisphere)	Freesurfer BA exvivo	4.92	✓
27146	Area of entorhinal (left hemisphere)	Freesurfer DKT	4.87	✓
27547	Volume of S-subparietal (left hemisphere)	Freesurfer a2009s	4.87	✓
25857	Volume of grey matter in Temporal Fusiform Cortex, posterior division (right)	Regional grey matter	4.70	✓
27351	Area of G-oc-temp-med-Parahip (left hemisphere)	Freesurfer a2009s	4.70	✓
27072	Area of entorhinal (left hemisphere)	Freesurfer BA exvivo	4.65	✓
27378	Area of S-collat-transv-ant (left hemisphere)	Freesurfer a2009s	4.64	✓
26828	Area of fusiform (right hemisphere)	Freesurfer desikan white	4.59	✓
26727	Area of fusiform (left hemisphere)	Freesurfer desikan white	4.54	✓
26962	Area of fusiform (right hemisphere)	Freesurfer desikan pial	4.51	✓
26658	Volume of fimbria (right hemisphere)	Freesurfer subsegmentation	4.45	✓
27758	Volume of S-oc-temp-lat (right hemisphere)	Freesurfer a2009s	4.42	✓
26895	Volume of fusiform (right hemisphere)	Freesurfer desikan white	4.31	✓
27610	Area of S-oc-temp-lat (right hemisphere)	Freesurfer a2009s	4.30	✓
27240	Area of fusiform (right hemisphere)	Freesurfer DKT	4.26	✓
27748	Volume of S-collat-transv-ant (right hemisphere)	Freesurfer a2009s	4.26	✓
25856	Volume of grey matter in Temporal Fusiform Cortex, posterior division (left)	Regional grey matter	4.20	✓
27250	Area of paracentral (right hemisphere)	Freesurfer DKT	4.16	✓
27302	Volume of fusiform (right hemisphere)	Freesurfer DKT	4.14	✓
26726	Area of entorhinal (left hemisphere)	Freesurfer desikan white	4.10	✓
27147	Area of fusiform (left hemisphere)	Freesurfer DKT	4.09	✓
27600	Area of S-collat-transv-ant (right hemisphere)	Freesurfer a2009s	4.03	✓
26838	Area of paracentral (right hemisphere)	Freesurfer desikan white	4.02	✓
26929	Area of fusiform (left hemisphere)	Freesurfer desikan pial	3.84	
27349	Area of G-oc-temp-lat-fusifor (left hemisphere)	Freesurfer a2009s	3.82	
27337	Area of G-cingul-Post-dorsal (left hemisphere)	Freesurfer a2009s	3.75	
25810	Volume of grey matter in Inferior Temporal Gyrus, posterior division (left)	Regional grey matter	3.73	
27526	Volume of S-collat-transv-ant (left hemisphere)	Freesurfer a2009s	3.73	
27614	Area of S-orbital-H-Shaped (right hemisphere)	Freesurfer a2009s	3.70	
26794	Volume of fusiform (left hemisphere)	Freesurfer desikan white	3.69	
26636	Volume of fimbria (left hemisphere)	Freesurfer subsegmentation	3.67	
27566	Area of G-front-sup (right hemisphere)	Freesurfer a2009s	3.63	
26972	Area of paracentral (right hemisphere)	Freesurfer desikan pial	3.63	

© 2022 Williams JA et al. *JAMA Psychiatry*.

27735	Volume of G-temporal-inf (right hemisphere)	Freesurfer a2009s	3.50	
27596	Area of S-cingul-Marginalis (right hemisphere)	Freesurfer a2009s	3.41	
27114	Area of entorhinal (right hemisphere)	Freesurfer BA exvivo	3.40	
25811	Volume of grey matter in Inferior Temporal Gyrus, posterior division (right)	Regional grey matter	3.38	
26897	Volume of inferiortemporal (right hemisphere)	Freesurfer desikan white	3.36	
27304	Volume of inferiortemporal (right hemisphere)	Freesurfer DKT	3.33	
27690	Mean thickness of S-pericallosal (right hemisphere)	Freesurfer a2009s	3.32	
27223	Volume of pericalcarine (left hemisphere)	Freesurfer DKT	-3.29	
27467	Mean thickness of S-parieto-occipital (left hemisphere)	Freesurfer a2009s	-3.34	
27082	Mean thickness of V1 (left hemisphere)	Freesurfer BA exvivo	-3.36	
26808	Volume of pericalcarine (left hemisphere)	Freesurfer desikan white	-3.44	
26779	Mean thickness of precuneus (left hemisphere)	Freesurfer desikan white	-3.48	
26670	Volume of CM (left hemisphere)	Freesurfer subsegmentation	-3.48	
27196	Mean thickness of precuneus (left hemisphere)	Freesurfer DKT	-3.52	
27432	Mean thickness of G-precuneus (left hemisphere)	Freesurfer a2009s	-3.59	
25863	Volume of grey matter in Frontal Operculum Cortex (right)	Regional grey matter	-3.59	
27207	Volume of cuneus (left hemisphere)	Freesurfer DKT	-3.60	
27444	Mean thickness of Pole-occipital (left hemisphere)	Freesurfer a2009s	-3.61	
25894	Volume of grey matter in I-IV Cerebellum (right)	Regional grey matter	-3.64	
27487	Volume of G-cuneus (left hemisphere)	Freesurfer a2009s	-3.67	
25883	Volume of grey matter in Putamen (right)	Regional grey matter	-3.78	
27518	Volume of Pole-occipital (left hemisphere)	Freesurfer a2009s	-3.79	
27096	Volume of V1 (left hemisphere)	Freesurfer BA exvivo	-3.92	✓
27590	Area of Lat-Fis-ant-Vertical (right hemisphere)	Freesurfer a2009s	-3.99	✓
27199	Mean thickness of superiorfrontal (left hemisphere)	Freesurfer DKT	-4.02	✓
26782	Mean thickness of superiorfrontal (left hemisphere)	Freesurfer desikan white	-4.26	✓
26555	Volume of Inf-Lat-Vent (left hemisphere)	Freesurfer ASEG	-4.35	✓
27738	Volume of Lat-Fis-ant-Vertical (right hemisphere)	Freesurfer a2009s	-4.37	✓
27418	Mean thickness of G-front-sup (left hemisphere)	Freesurfer a2009s	-5.11	✓

Trait code is the phenotypic code assigned to the variable in UK Biobank. Significant is ticked if the trait is associated with genetically-predicted levels of interleukin-6 at a multiply-corrected level of statistical significance ($p < 1.1\times10^{-4}$), unticked if only associated at a suggestive level of statistical significance ($p < 0.001$).

© 2022 Williams JA et al. *JAMA Psychiatry*.

eTable 4. Brain imaging measures associated with genetically predicted levels of other inflammatory biomarkers at a suggestive level of statistical significance

Biomarker	Trait code	Trait name	Trait category	Z-score
IL-1	27142	Volume of entorhinal (right hemisphere)	Freesurfer BA exvivo	-3.33
IL-2	26598	Volume of choroid-plexus (right hemisphere)	Freesurfer ASEG	3.33
CRP	27603	Area of S-front-middle (right hemisphere)	Freesurfer subsegmentation	3.41
CRP	27751	Volume of S-front-middle (right hemisphere)	Freesurfer a2009s	3.61
BDNF	26595	Volume of Accumbens-area (right hemisphere)	Freesurfer ASEG	-3.30
BDNF	26689	Volume of MGN (right hemisphere)	Freesurfer subsegmentation	-3.52

Trait code is the phenotypic code assigned to the variable in UK Biobank. All associations are at a suggestive level of statistical significance ($p < 0.001$) only.

Abbreviations: IL-1, interleukin-1; IL-2, interleukin-2; CRP, C-reactive protein; BDNF, brain-derived neurotrophic factor.

© 2022 Williams JA et al. *JAMA Psychiatry*.

eTable 5. Brain imaging measures from eTable 3 associated with genetically predicted levels of interleukin 6, including adjustments for brain size and excluding psychiatric illness

Trait name	Z-score Brain Volume Adjustment	Significant Brain Volume Adjustment	Z-score Excluding Psych	Significant Excluding Psych
Area of perirhinal (left hemisphere)	5.89	✓	5.69	✓
Volume of grey matter in Middle Temporal Gyrus, temporooccipital part (right)	6.04	✓	5.55	✓
Area of S-subparietal (left hemisphere)	5.08	✓	4.59	✓
Volume of entorhinal (left hemisphere)	4.86	✓	4.56	✓
Area of entorhinal (left hemisphere)	5.00	✓	4.62	✓
Volume of S-subparietal (left hemisphere)	5.08	✓	4.53	✓
Volume of grey matter in Temporal Fusiform Cortex, posterior division (right)	5.16	✓	4.49	✓
Area of G-oc-temp-med-Parahip (left hemisphere)	4.58	✓	4.67	✓
Area of entorhinal (left hemisphere)	4.71	✓	4.34	✓
Area of S-collat-transv-ant (left hemisphere)	4.67	✓	4.41	✓
Area of fusiform (right hemisphere)	5.45	✓	4.10	✓
Area of fusiform (left hemisphere)	5.01	✓	4.32	✓
Area of fusiform (right hemisphere)	5.20	✓	4.10	✓
Volume of fimbria (right hemisphere)	4.45	✓	3.65	
Volume of S-oc-temp-lat (right hemisphere)	3.78		4.51	✓
Volume of fusiform (right hemisphere)	5.20	✓	3.90	✓
Area of S-oc-temp-lat (right hemisphere)	4.00	✓	4.09	✓
Area of fusiform (right hemisphere)	4.91	✓	3.82	
Volume of S-collat-transv-ant (right hemisphere)	3.95	✓	3.90	✓
Volume of grey matter in Temporal Fusiform Cortex, posterior division (left)	4.64	✓	3.80	
Area of paracentral (right hemisphere)	4.18	✓	3.86	✓

© 2022 Williams JA et al. *JAMA Psychiatry*.

Volume of fusiform (right hemisphere)	4.80	✓	3.75	
Area of entorhinal (left hemisphere)	4.16	✓	4.05	✓
Area of fusiform (left hemisphere)	4.40	✓	3.87	✓
Area of S-collat-transv-ant (right hemisphere)	3.88	✓	3.64	
Area of paracentral (right hemisphere)	4.02	✓	3.70	
Area of fusiform (left hemisphere)	4.38	✓	3.72	
Area of G-oc-temp-lat-fusifor (left hemisphere)	3.99	✓	3.53	
Area of G-cingul-Post-dorsal (left hemisphere)	3.96	✓	3.14	
Volume of grey matter in Inferior Temporal Gyrus, posterior division (left)	3.90	✓	3.08	
Volume of S-collat-transv-ant (left hemisphere)	3.86	✓	3.38	
Area of S-orbital-H-Shaped (right hemisphere)	3.55		3.32	
Volume of fusiform (left hemisphere)	4.29	✓	3.48	
Volume of fimbria (left hemisphere)	3.67		3.23	
Area of G-front-sup (right hemisphere)	3.92	✓	3.89	✓
Area of paracentral (right hemisphere)	3.58		3.20	
Volume of G-temporal-inf (right hemisphere)	3.32		3.23	
Area of S-cingul-Marginalis (right hemisphere)	3.39		2.77	
Area of entorhinal (right hemisphere)	3.59		3.10	
Volume of grey matter in Inferior Temporal Gyrus, posterior division (right)	3.67		3.04	
Volume of inferiortemporal (right hemisphere)	3.33		3.09	
Volume of inferiortemporal (right hemisphere)	3.43		3.03	
Mean thickness of S-pericallosal (right hemisphere)	3.77		3.46	
Volume of pericalcarine (left hemisphere)	-2.98		-3.29	
Mean thickness of S-parieto-occipital (left hemisphere)	-2.92		-3.61	
Mean thickness of V1 (left hemisphere)	-2.51		-3.43	

© 2022 Williams JA et al. *JAMA Psychiatry*.

Volume of pericalcarine (left hemisphere)	-3.13		-3.35	
Mean thickness of precuneus (left hemisphere)	-2.88		-3.68	
Volume of CM (left hemisphere)	-3.48		-3.47	
Mean thickness of precuneus (left hemisphere)	-2.99		-3.71	
Mean thickness of G-precuneus (left hemisphere)	-3.07		-3.68	
Volume of grey matter in Frontal Operculum Cortex (right)	-3.81		-3.64	
Volume of cuneus (left hemisphere)	-3.30		-3.73	
Mean thickness of Pole-occipital (left hemisphere)	-3.04		-3.94	✓
Volume of grey matter in I-IV Cerebellum (right)	-3.79		-3.56	
Volume of G-cuneus (left hemisphere)	-3.45		-3.95	✓
Volume of grey matter in Putamen (right)	-3.92	✓	-3.84	
Volume of Pole-occipital (left hemisphere)	-3.50		-4.16	✓
Volume of V1 (left hemisphere)	-3.58		-3.98	✓
Area of Lat-Fis-ant-Vertical (right hemisphere)	-3.40		-4.13	✓
Mean thickness of superiorfrontal (left hemisphere)	-3.40		-3.93	✓
Mean thickness of superiorfrontal (left hemisphere)	-3.64		-4.15	✓
Volume of Inf-Lat-Vent (left hemisphere)	-4.16	✓	-4.52	✓
Volume of Lat-Fis-ant-Vertical (right hemisphere)	-3.89	✓	-4.46	✓
Mean thickness of G-front-sup (left hemisphere)	-4.48	✓	-5.05	✓

Significant is ticked if the trait is associated with genetically-predicted levels of interleukin-6 at a multiply-corrected level of statistical significance ($p < 1.1\times10^{-4}$), unticked if only associated at a suggestive level of statistical significance ($p < 0.001$). Ordering is identical to Supplementary Table 3 (ordered by original z-score).

© 2022 Williams JA et al. *JAMA Psychiatry*.

eTable 6. Brain imaging measures from eTable 4 associated with genetically predicted levels of other inflammatory biomarkers, including adjustments for brain size and excluding psychiatric illness

Biomarker	Trait name	Z-score Brain Volume Adjustment	Z-Score Excluding Psych
IL-1	Volume of entorhinal (right hemisphere)	-3.374842	-3.153815
IL-2	Volume of choroid-plexus (right hemisphere)	3.431921	3.143175
CRP	Area of S-front-middle (right hemisphere)	4.136389	3.557391
CRP	Volume of S-front-middle (right hemisphere)	4.267456	3.739790
BDNF	Volume of Accumbens-area (right hemisphere)	-3.582643	-3.320492
BDNF	Volume of MGN (right hemisphere)	-3.515515	-3.454658

Associations are at a suggestive level of statistical significance ($p < 0.001$) only. Ordering is identical to Supplementary Table 4 (ordered by original z-score). Abbreviations: IL-1, interleukin-1; IL-2, interleukin-2; CRP, C-reactive protein; BDNF, brain-derived neurotrophic factor.

© 2022 Williams JA et al. *JAMA Psychiatry*.

eFigure 1. Heat map of associations between genetically predicted inflammatory biomarkers and brain imaging measures in subcategory 1101 (regional grey matter)

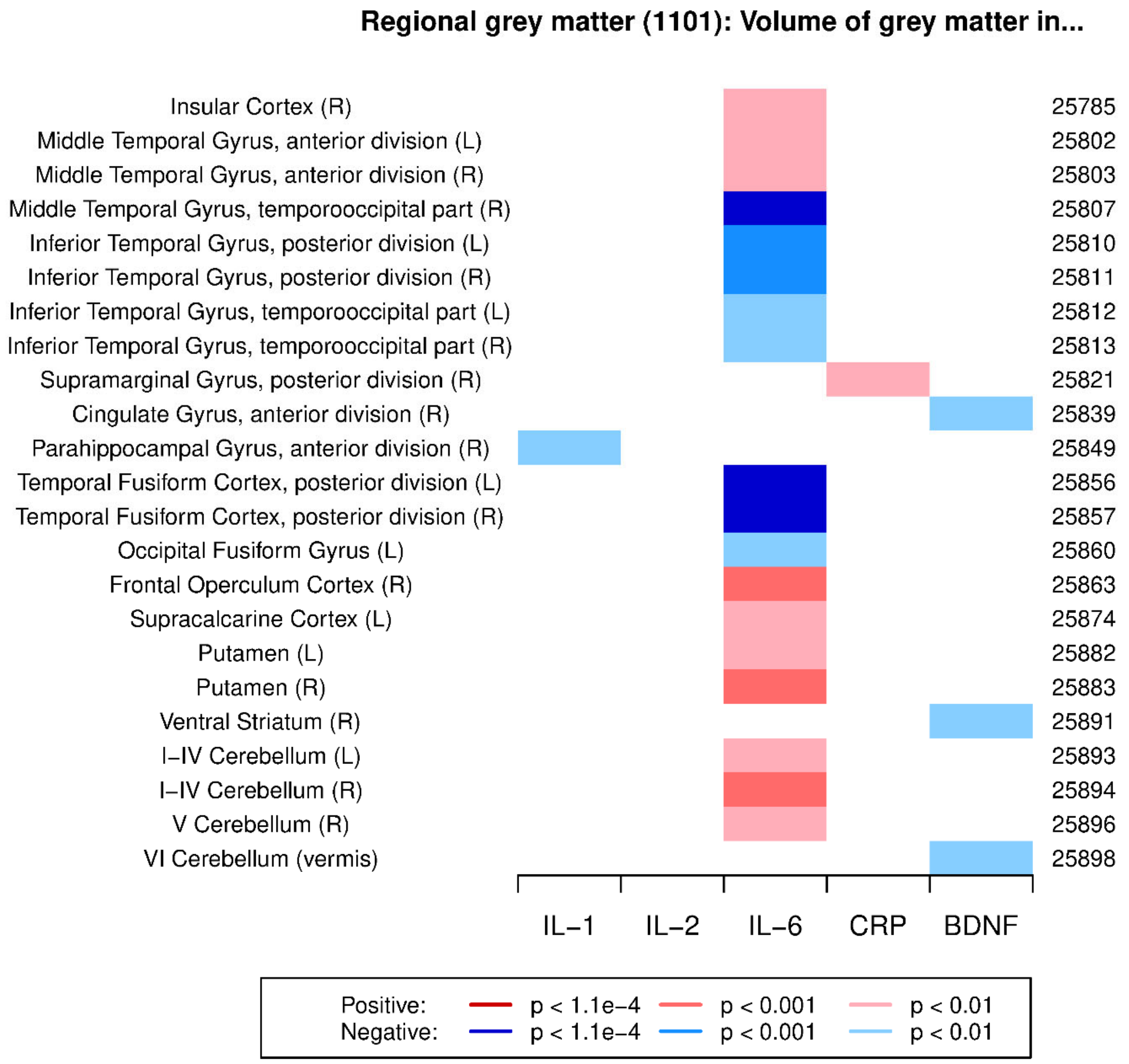

© 2022 Williams JA et al. *JAMA Psychiatry*.

eFigure 2. Heat map of associations between genetically predicted inflammatory biomarkers and brain imaging measures in subcategory 190 (Freesurfer ASEG)

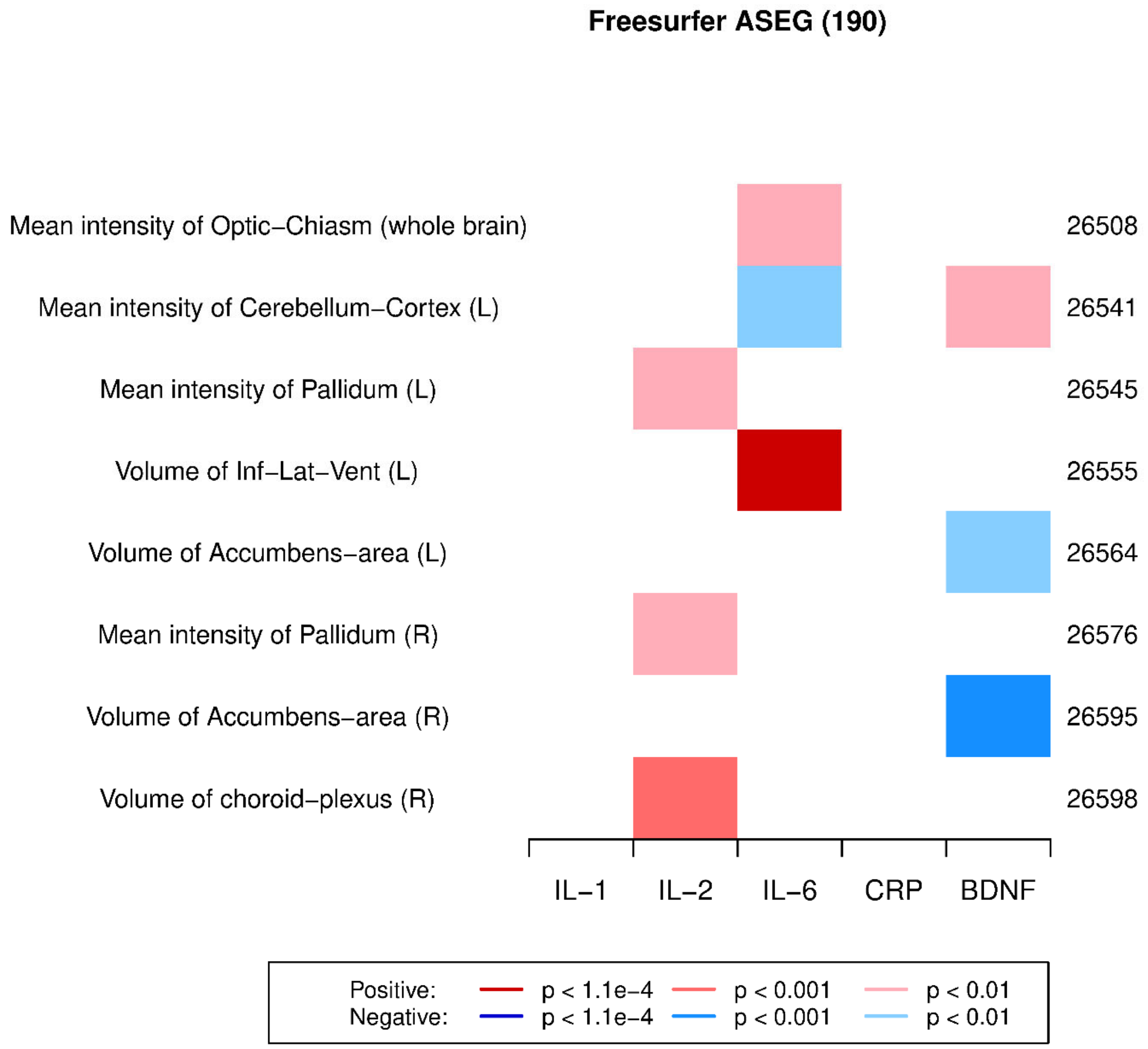

© 2022 Williams JA et al. *JAMA Psychiatry*.

eFigure 3. Heat map of associations between genetically predicted inflammatory biomarkers and brain imaging measures in subcategory 191 (Freesurfer subsegmentation)

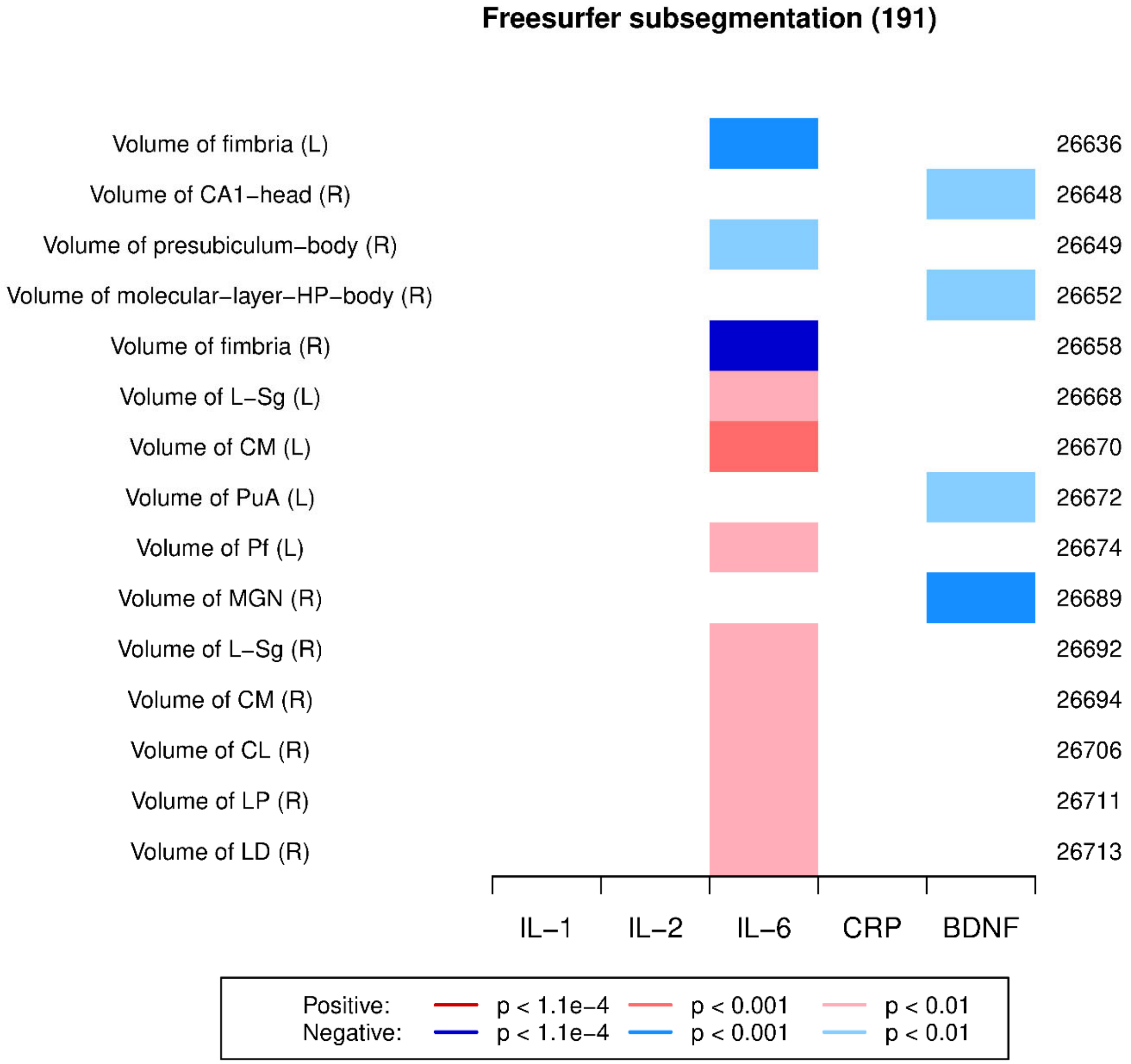

© 2022 Williams JA et al. *JAMA Psychiatry*.

eFigure 4. Heat map of associations between genetically predicted inflammatory biomarkers and brain imaging measures in subcategory 192 (Freesurfer desikan white)

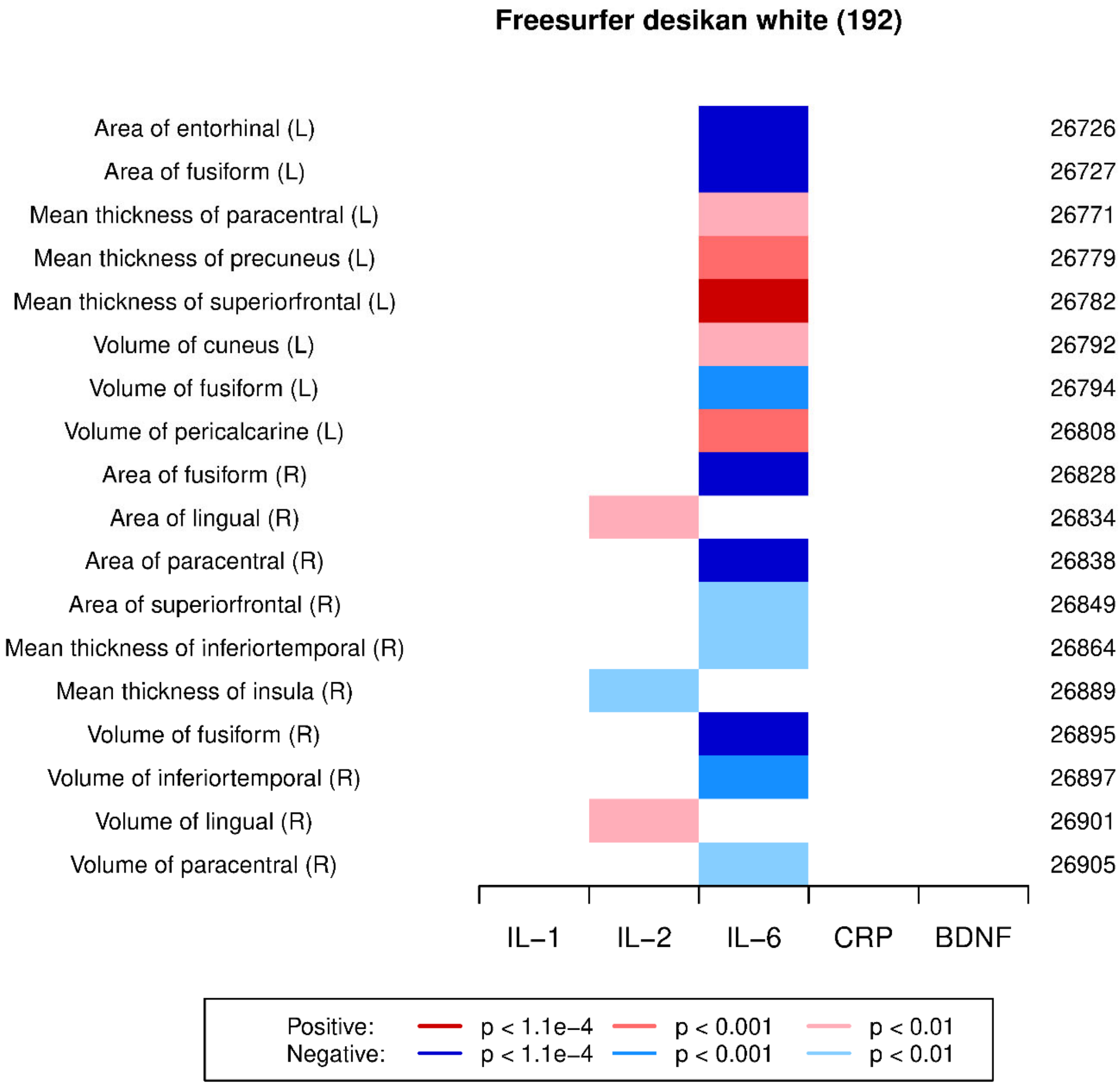

© 2022 Williams JA et al. *JAMA Psychiatry*.

eFigure 5. Heat map of associations between genetically predicted inflammatory biomarkers and brain imaging measures in subcategory 193 (Freesurfer desikan pial)

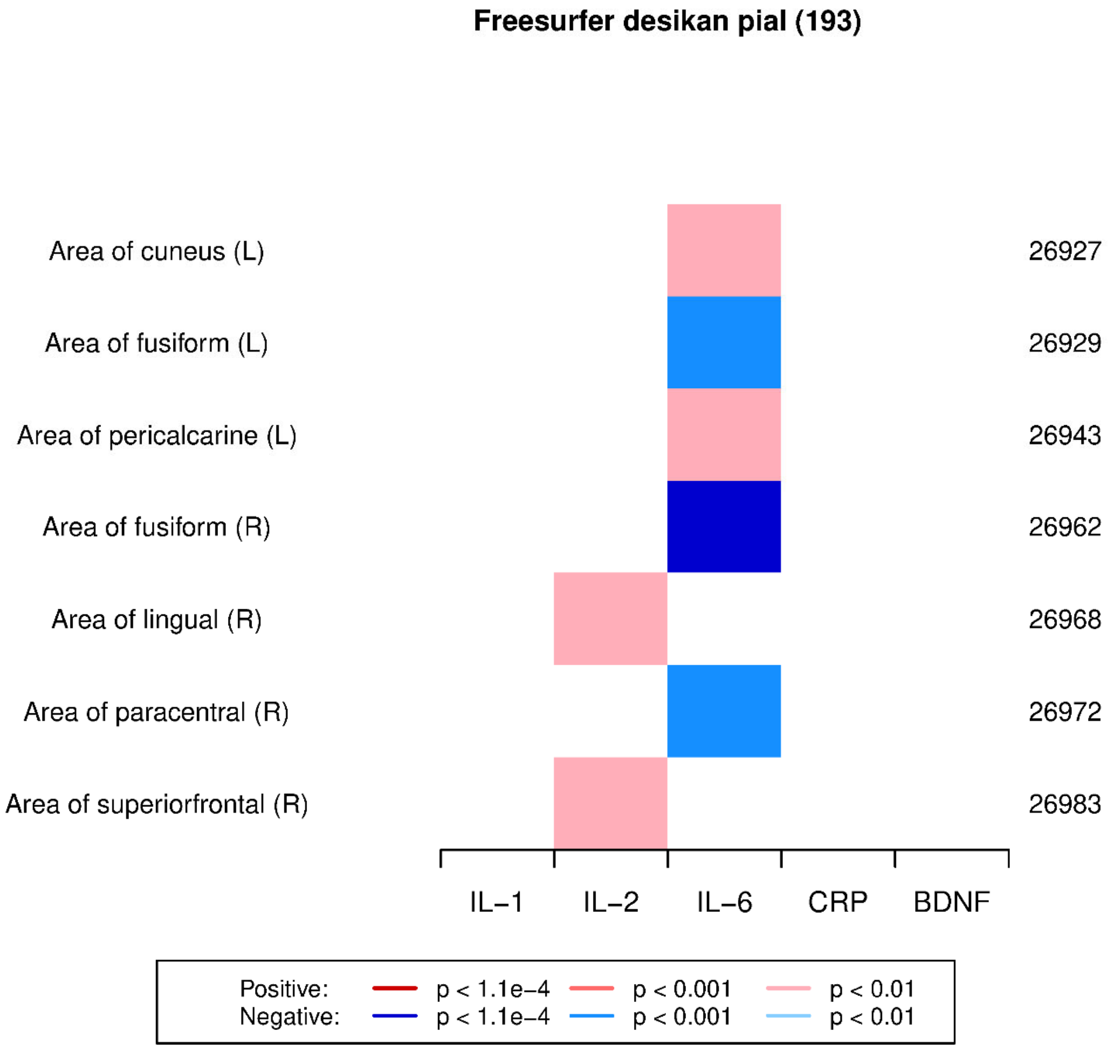

© 2022 Williams JA et al. *JAMA Psychiatry*.

eFigure 6. Heat map of associations between genetically predicted inflammatory biomarkers and brain imaging measures in subcategory 194 (Freesurfer desikan grey-white)

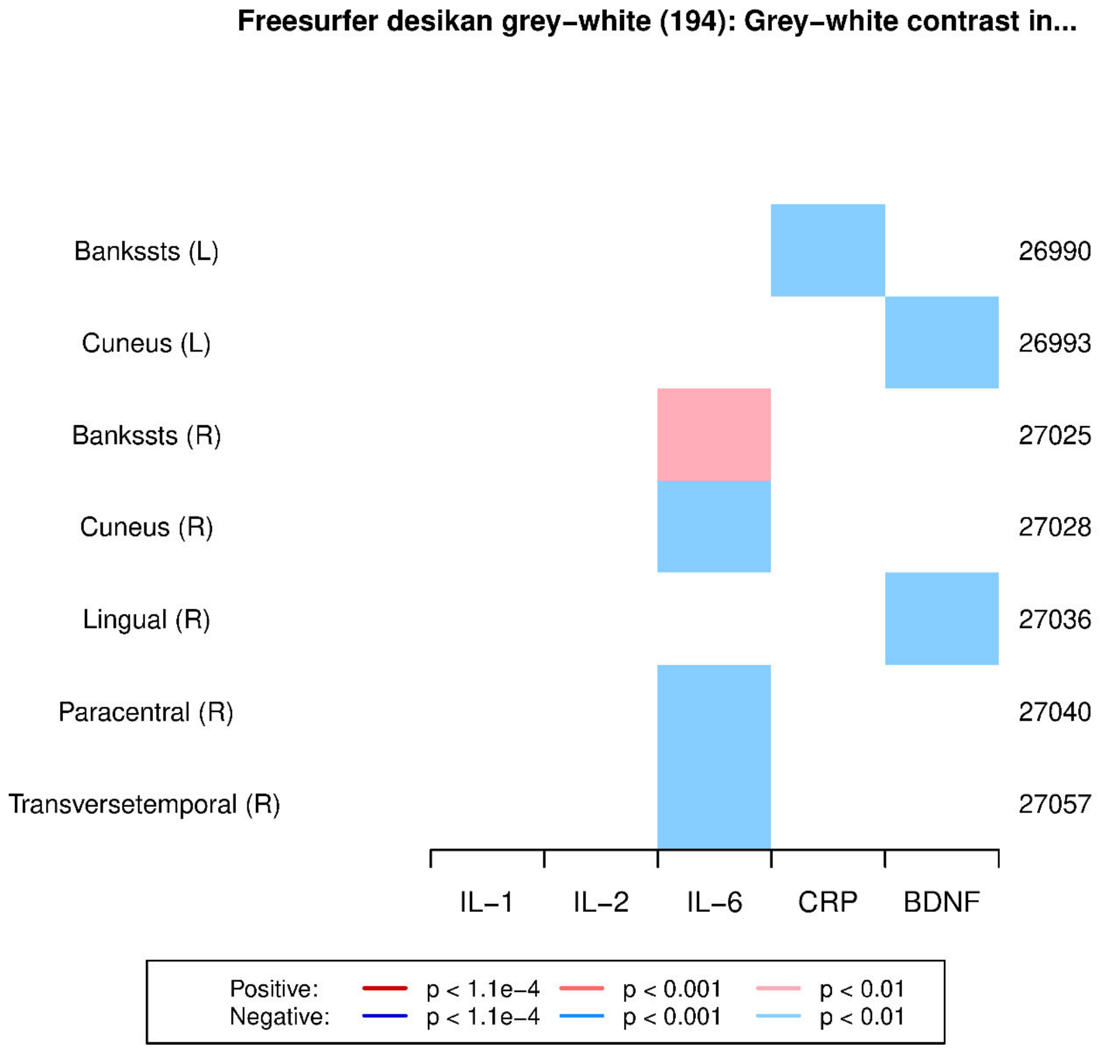

© 2022 Williams JA et al. *JAMA Psychiatry*.

eFigure 7. Heat map of associations between genetically predicted inflammatory biomarkers and brain imaging measures in subcategory 195 (Freesurfer BA exvivo)

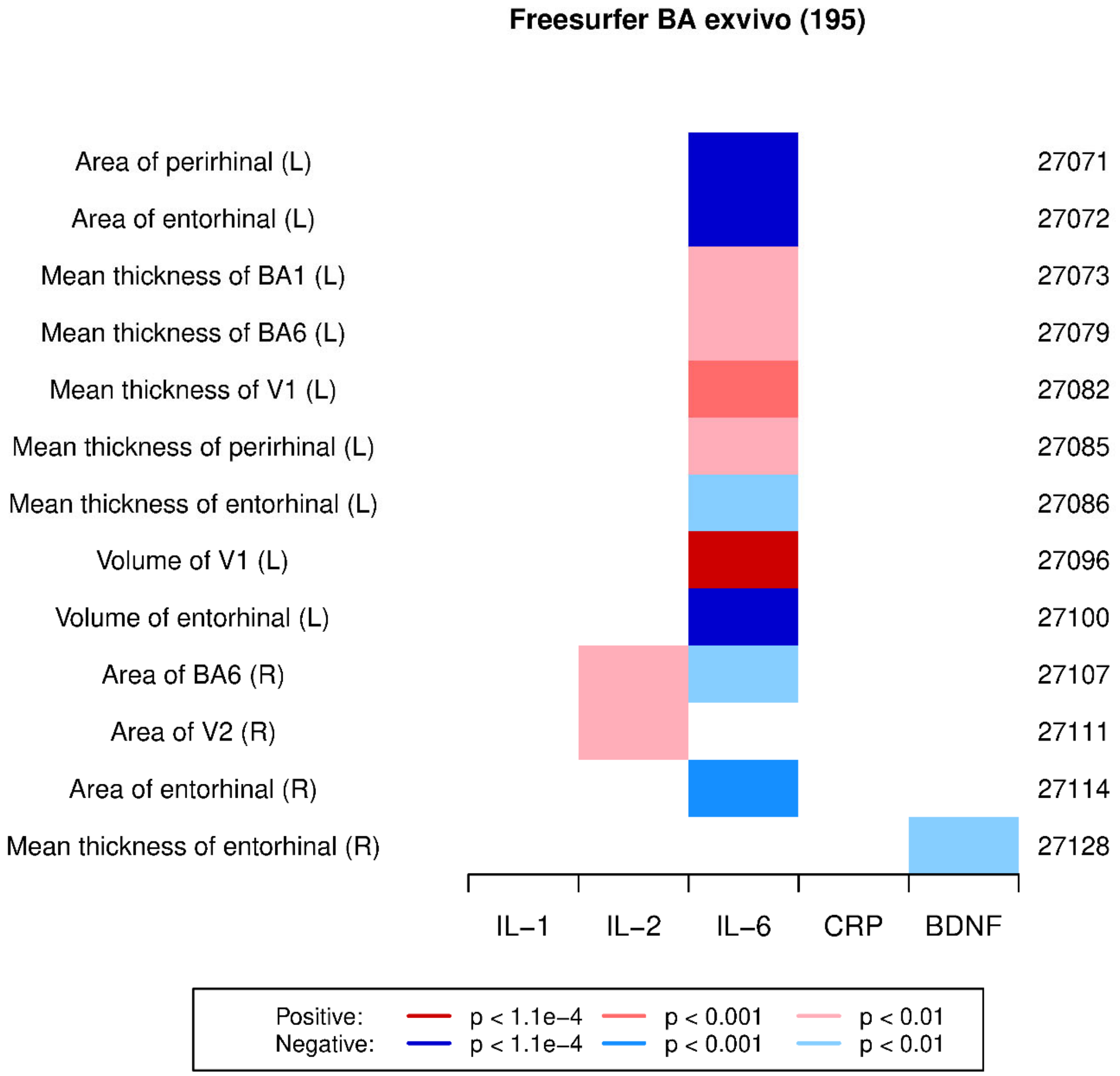

© 2022 Williams JA et al. *JAMA Psychiatry*.

eFigure 8. Heat map of associations between genetically predicted inflammatory biomarkers and brain imaging measures in subcategory 196 (Freesurfer DKT)

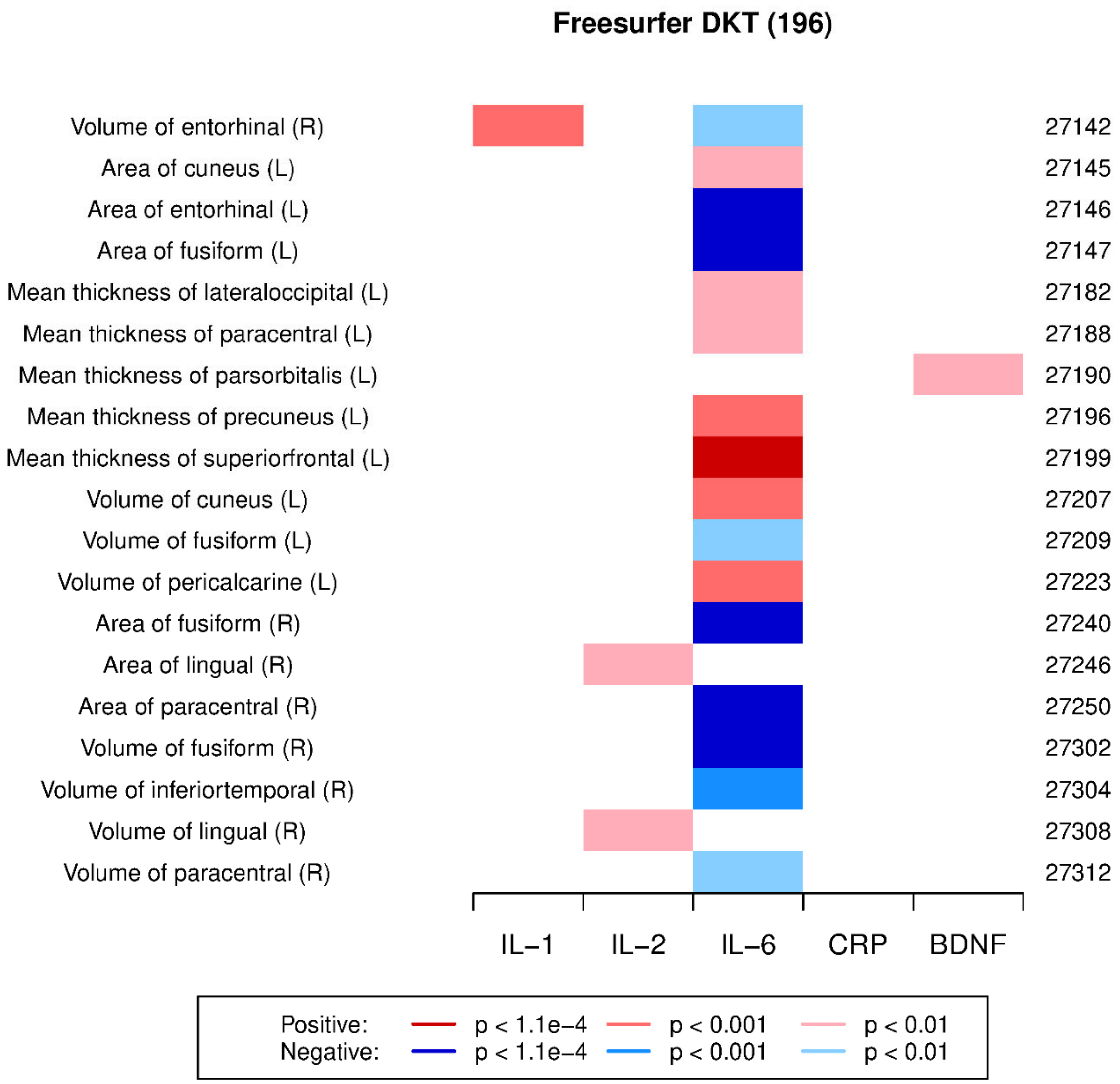

© 2022 Williams JA et al. *JAMA Psychiatry*.

eFigure 9. Heat map of associations between genetically predicted inflammatory biomarkers and brain imaging measures in subcategory 197 (Freesurfer a2009s)

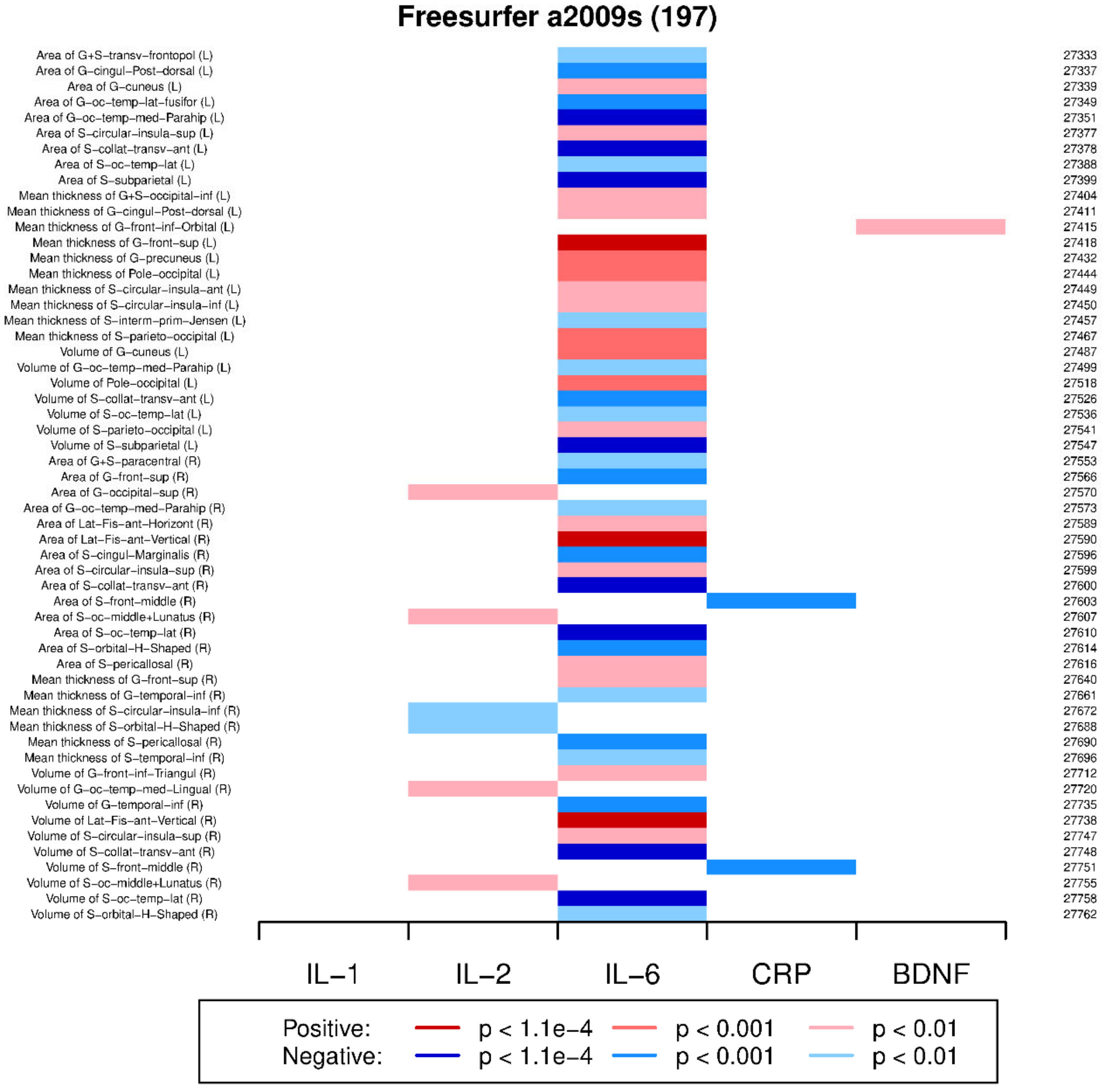

© 2022 Williams JA et al. *JAMA Psychiatry*.

eFigure 10. Differential gene expression in fro vs whole brain

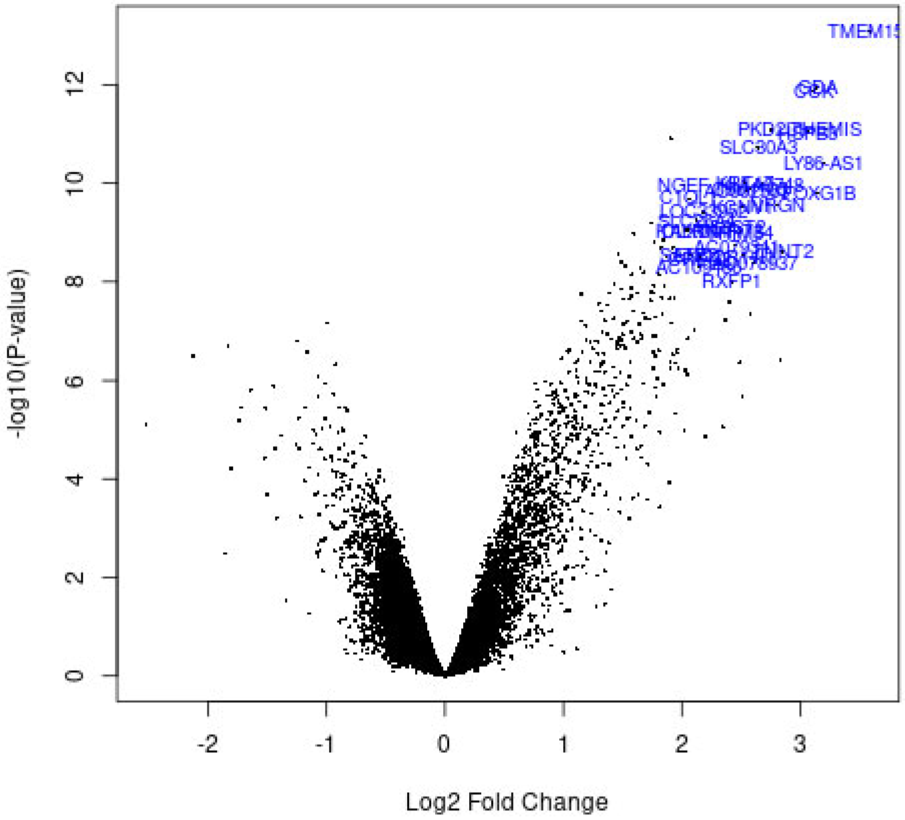

© 2022 Williams JA et al. *JAMA Psychiatry*.

eFigure 11. Differential gene expression in FuG vs whole brain

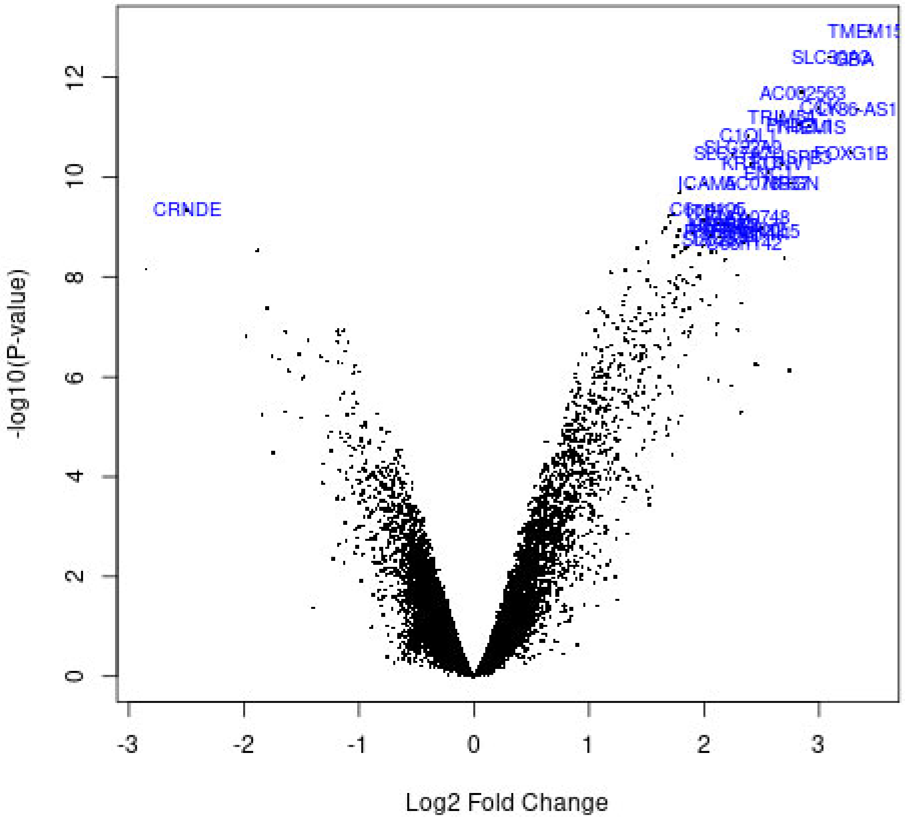

eFigure 12. Differential gene expression in ITG vs whole brain

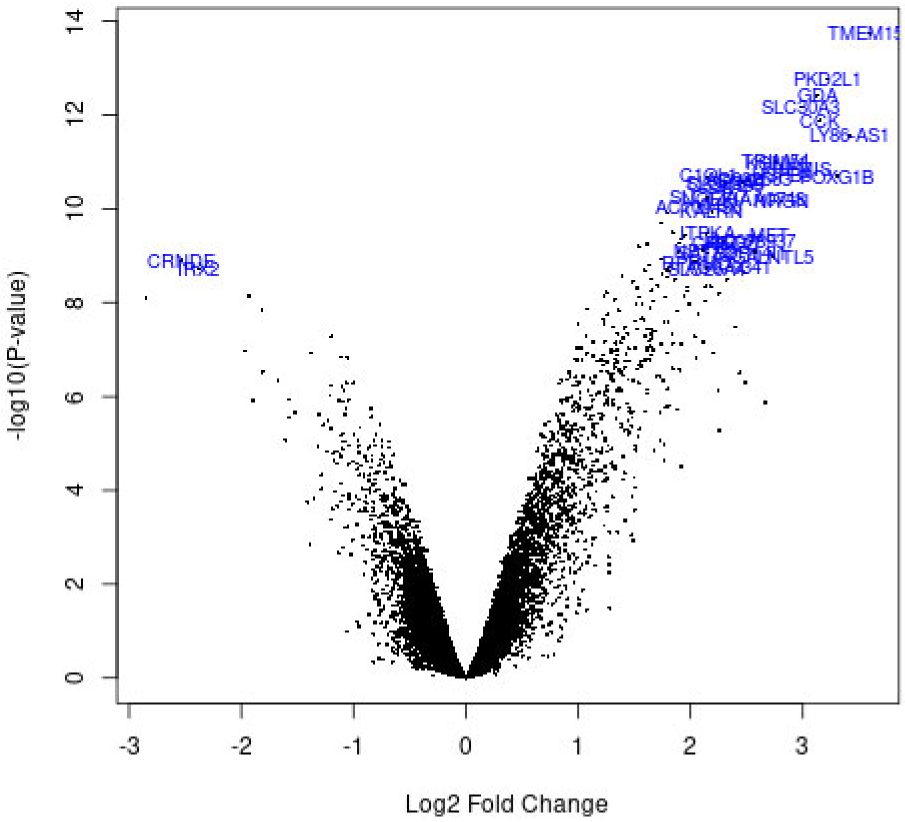

© 2022 Williams JA et al. *JAMA Psychiatry*.

eFigure 13. Differential gene expression in PCu vs whole brain

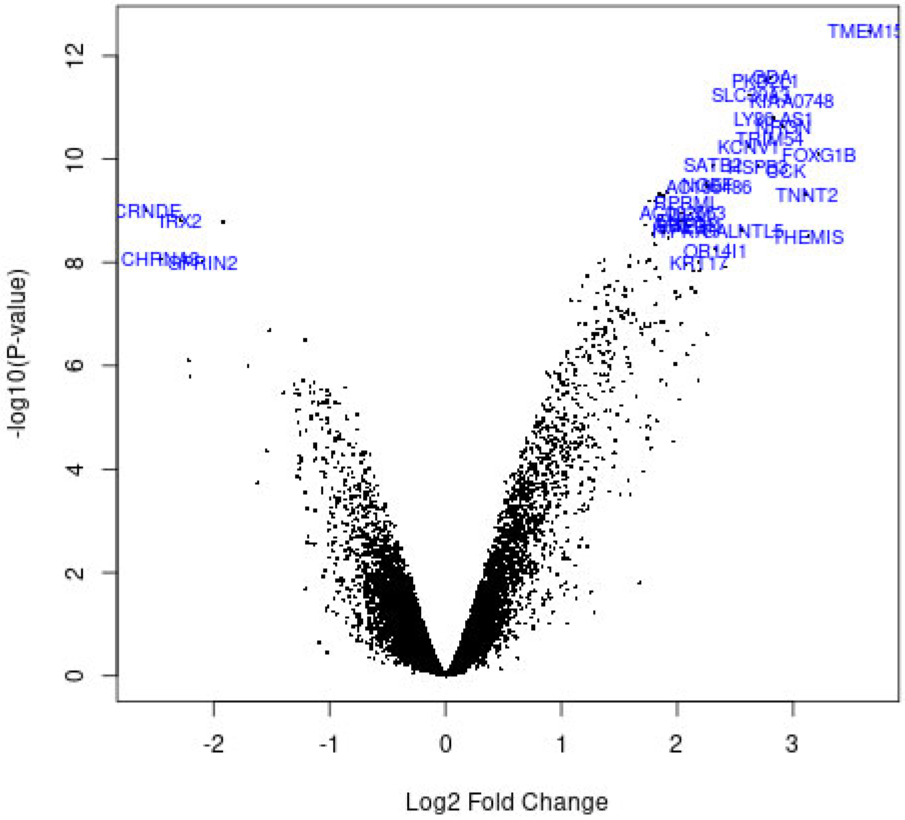

© 2022 Williams JA et al. *JAMA Psychiatry*.

eFigure 14. Differential gene expression in Pu vs whole brain

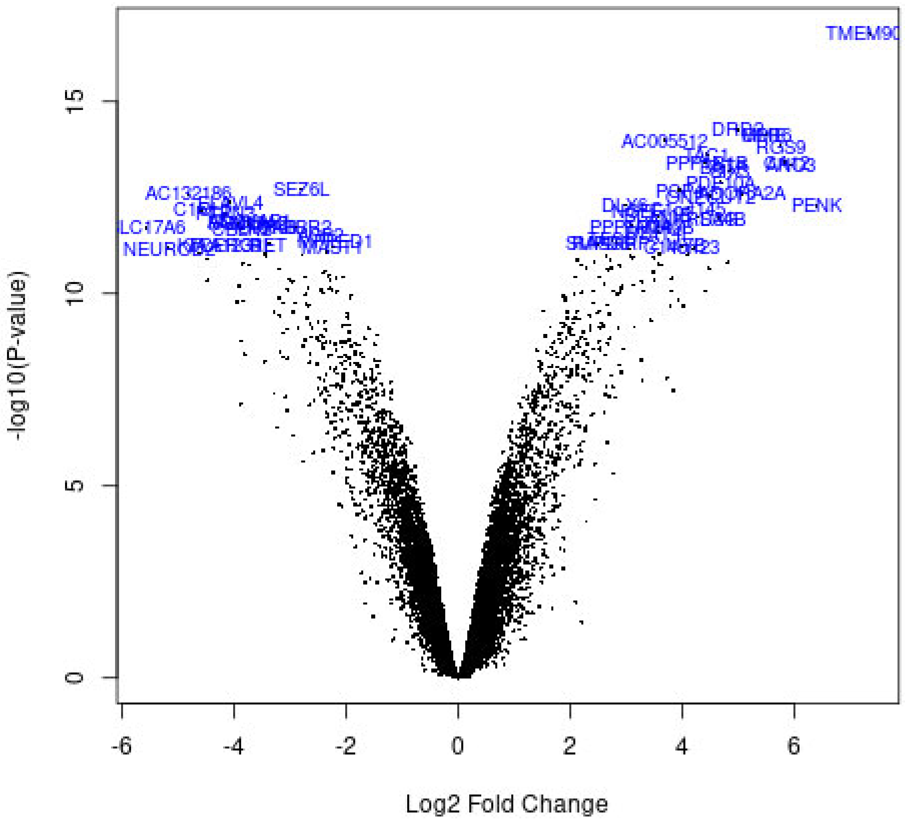

© 2022 Williams JA et al. *JAMA Psychiatry*.

eFigure 15. Differential gene expression in SFG vs whole brain

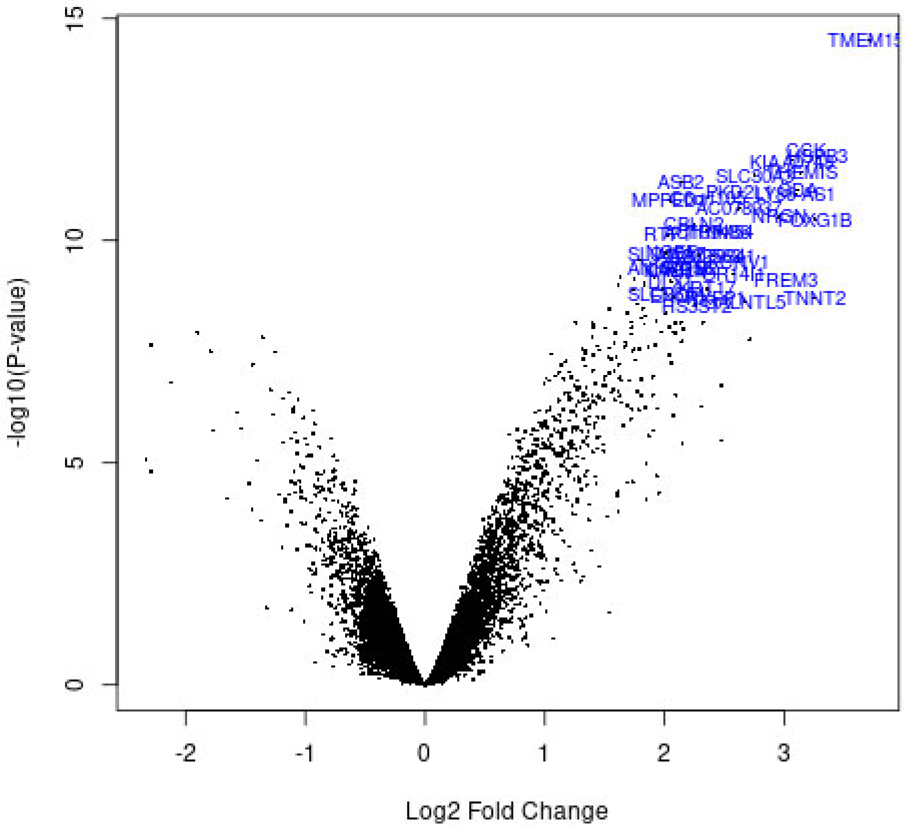

© 2022 Williams JA et al. *JAMA Psychiatry*.

eFigure 16. Differential gene expression in Vel_IV vs whole brain

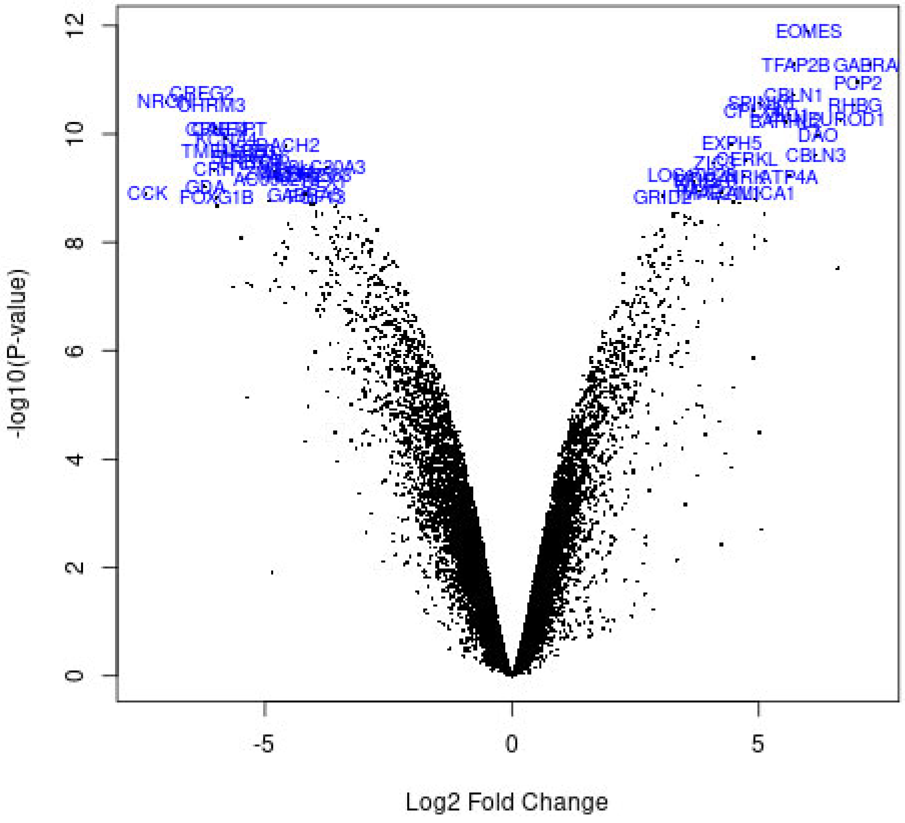

© 2022 Williams JA et al. *JAMA Psychiatry*.

eTable 7. Differential expression results in the fro

Genes with a log-fold change >2 and a false discovery rate < .05 are shown.

BrainRegion	Gene	logFC	t	P.Value	adj.P.Val	B
fro	ANXA8	2.19	6.54	1.35e-05	8.62e-04	3.34
fro	NEUROD6	2.02	6.71	1.02e-05	7.04e-04	3.62
fro	NPPA	2.35	6.81	8.78e-06	6.34e-04	3.77
fro	LOC646627	2.51	7.73	2.11e-06	2.16e-04	5.21
fro	FEZF2	2.05	8.46	7.47e-07	9.93e-05	6.25
fro	EGR3	2.03	8.59	6.20e-07	8.57e-05	6.44
fro	SLN	2.49	8.85	4.37e-07	6.61e-05	6.79
fro	FREM3	2.83	8.93	3.92e-07	6.14e-05	6.89
fro	MOXD1	2.05	9.72	1.40e-07	2.97e-05	7.92
fro	LHX2	2.10	9.99	9.95e-08	2.34e-05	8.25
fro	FRMPD2	2.37	10.41	6.01e-08	1.58e-05	8.75
fro	NPY	2.58	10.67	4.42e-08	1.25e-05	9.05
fro	GALNTL5	2.40	11.13	2.60e-08	8.03e-06	9.57
fro	RXFP1	2.42	12.02	9.82e-09	4.15e-06	10.51
fro	AC109486	2.1	12.	4.73e-	2.27e-	11.

© 2022 Williams JA et al. *JAMA Psychiatry*.

	.1	5	72	09	06	21
fro	AC078937.4	2.61	12.86	4.12e-09	2.03e-06	11.34
fro	CREG2	2.15	13.07	3.34e-09	1.73e-06	11.54
fro	SATB2	2.06	13.23	2.85e-09	1.60e-06	11.69
fro	OR14I1	2.52	13.22	2.89e-09	1.60e-06	11.68
fro	ENC1	2.14	13.20	2.93e-09	1.60e-06	11.67
fro	TNNT2	2.84	13.40	2.43e-09	1.53e-06	11.84
fro	AC079341.1	2.46	13.71	1.81e-09	1.25e-06	12.12
fro	TRIM54	2.49	14.35	9.93e-10	7.92e-07	12.68
fro	KALRN	2.05	14.47	8.91e-10	7.56e-07	12.78
fro	DLX1	2.03	14.45	9.06e-10	7.56e-07	12.76
fro	DMRT3	2.42	14.44	9.12e-10	7.56e-07	12.76
fro	HS3ST2	2.41	14.68	7.39e-10	6.97e-07	12.95
fro	SLC26A4	2.12	15.01	5.51e-10	5.71e-07	13.22
fro	LOC339524	2.18	15.43	3.83e-10	4.18e-07	13.55
fro	KCNV1	2.50	15.73	2.97e-10	3.42e-07	13.79
fro	NRGN	2.80	15.81	2.77e-10	3.38e-07	13.85
fro	C1QL1	2.0	16.	2.08e-	2.70e-	14.

© 2022 Williams JA et al. *JAMA Psychiatry*.

		6	15	10	07	11
fro	FOXG1B	3.15	16.50	1.58e-10	2.18e-07	14.36
fro	AC002563.2	2.54	16.64	1.41e-10	2.08e-07	14.46
fro	C6orf105	2.57	16.75	1.29e-10	2.05e-07	14.54
fro	KRT17	2.53	17.11	9.70e-11	1.87e-07	14.79
fro	KIAA0748	2.67	16.99	1.06e-10	1.87e-07	14.71
fro	NGEF	2.01	16.97	1.08e-10	1.87e-07	14.69
fro	LY86-AS1	3.19	18.33	3.87e-11	8.91e-08	15.59
fro	SLC30A3	2.64	19.36	1.86e-11	4.83e-08	16.22
fro	THEMIS	3.21	20.65	7.79e-12	3.16e-08	16.94
fro	PKD2L1	2.75	20.59	8.11e-12	3.16e-08	16.91
fro	HSPB3	3.06	20.41	9.15e-12	3.16e-08	16.81
fro	GDA	3.14	23.81	1.13e-12	9.00e-09	18.48
fro	CCK	3.11	23.56	1.30e-12	9.00e-09	18.37
fro	TMEM155	3.59	28.82	8.36e-14	1.73e-09	20.36

© 2022 Williams JA et al. *JAMA Psychiatry*.

eTable 8. Differential expression results in the Fug

Genes with a log-fold change >2 and a false discovery rate < .05 are shown.

BrainRegion	Gene	logFC	t	P.Value	adj.P.Val	B
FuG	ANXA8	2.32	6.86	5.14e-06	3.32e-04	4.23
FuG	NPY	2.24	7.62	1.48e-06	1.24e-04	5.48
FuG	NEUROD6	2.13	7.73	1.23e-06	1.05e-04	5.67
FuG	FREM3	2.03	7.81	1.09e-06	9.38e-05	5.79
FuG	NPPA	2.74	8.04	7.60e-07	7.40e-05	6.15
FuG	GLP2R	2.47	8.21	5.89e-07	6.05e-05	6.41
FuG	LOC646627	2.44	8.25	5.54e-07	5.81e-05	6.47
FuG	FRMPD2	2.11	9.15	1.47e-07	2.13e-05	7.80
FuG	SLN	2.29	9.31	1.18e-07	1.89e-05	8.02
FuG	CAMK2A	2.11	9.52	8.74e-08	1.49e-05	8.32
FuG	MOXD1	2.05	10.17	3.65e-08	7.73e-06	9.18
FuG	EGR3	2.33	10.23	3.40e-08	7.27e-06	9.25
FuG	FEZF2	2.11	10.48	2.45e-08	5.58e-06	9.58
FuG	CREG2	2.29	11.26	9.34e-09	2.72e-06	10.52
FuG	AC079341.1	2.18	11.86	4.61e-09	1.49e-06	11.21
FuG	MET	2.7	11.	4.24e-	1.40e-	11.

© 2022 Williams JA et al. *JAMA Psychiatry*.

		0	93	09	06	29
FuG	DLX1	2.11	12.16	3.24e-09	1.16e-06	11.55
FuG	DYDC2	2.06	12.18	3.18e-09	1.16e-06	11.57
FuG	C6orf142	2.34	12.56	2.06e-09	9.11e-07	11.99
FuG	SLC26A4	2.14	12.78	1.62e-09	7.49e-07	12.22
FuG	TAC3	2.07	12.82	1.56e-09	7.36e-07	12.26
FuG	LHX2	2.50	13.17	1.07e-09	6.29e-07	12.61
FuG	GALNTL5	2.47	13.04	1.22e-09	6.29e-07	12.49
FuG	RXFP1	2.08	13.02	1.25e-09	6.29e-07	12.47
FuG	OR14I1	2.49	13.02	1.26e-09	6.29e-07	12.46
FuG	NPTX2	2.21	13.29	9.44e-10	5.93e-07	12.74
FuG	KALRN	2.13	13.38	8.61e-10	5.71e-07	12.82
FuG	HS3ST2	2.17	13.35	8.81e-10	5.71e-07	12.80
FuG	KIAA0748	2.38	13.68	6.27e-10	4.64e-07	13.13
FuG	ITPKA	2.08	13.94	4.83e-10	4.01e-07	13.37
FuG	C6orf105	2.03	13.99	4.57e-10	3.95e-07	13.42
FuG	AC078937.4	2.54	15.31	1.28e-10	1.43e-07	14.62
FuG	NRGN	2.76	15.27	1.32e-10	1.43e-07	14.59
FuG	ICAM5	2.02	15.23	1.38e-10	1.43e-07	14.55

© 2022 Williams JA et al. *JAMA Psychiatry*.

FuG	ENC1	2.56	15.81	8.07e-11	9.84e-08	15.04
FuG	KCNV1	2.68	16.25	5.42e-11	7.05e-08	15.41
FuG	KRT17	2.41	16.25	5.44e-11	7.05e-08	15.40
FuG	HSPB3	2.84	16.63	3.90e-11	5.77e-08	15.71
FuG	FOXG1B	3.28	16.83	3.27e-11	5.40e-08	15.87
FuG	SLC17A7	2.25	16.79	3.38e-11	5.40e-08	15.83
FuG	SLC22A9	2.34	17.14	2.52e-11	4.75e-08	16.10
FuG	C1QL1	2.39	17.77	1.50e-11	3.10e-08	16.57
FuG	PKD2L1	2.83	18.44	8.75e-12	2.21e-08	17.04
FuG	THEMIS	2.91	18.32	9.61e-12	2.21e-08	16.96
FuG	TRIM54	2.66	18.91	6.07e-12	1.80e-08	17.36
FuG	CCK	2.99	19.45	4.02e-12	1.48e-08	17.72
FuG	LY86-AS1	3.33	19.37	4.27e-12	1.48e-08	17.67
FuG	AC002563.2	2.85	20.40	2.00e-12	1.03e-08	18.32
FuG	SLC30A3	3.10	22.78	3.96e-13	2.95e-09	19.66
FuG	GDA	3.30	22.66	4.27e-13	2.95e-09	19.60
FuG	TMEM155	3.44	24.66	1.23e-13	2.55e-09	20.60

© 2022 Williams JA et al. *JAMA Psychiatry*.

eTable 9. Differential expression results in the ITG

Genes with a log-fold change >2 and a false discovery rate < .05 are shown.

BrainRegion	Gene	logFC	t	P.Value	adj.P.Val	B
ITG	GLP2R	2.26	6.91	5.43e-06	3.30e-04	4.17
ITG	ANXA8	2.25	6.94	5.17e-06	3.20e-04	4.22
ITG	NPPA	2.67	7.80	1.32e-06	1.09e-04	5.61
ITG	NEUROD6	2.25	8.16	7.61e-07	7.01e-05	6.16
ITG	LOC646627	2.48	8.44	4.99e-07	5.04e-05	6.58
ITG	FREM3	2.44	8.77	3.13e-07	3.59e-05	7.05
ITG	CAMK2A	2.05	9.26	1.58e-07	2.21e-05	7.74
ITG	TNNT2	2.20	9.46	1.20e-07	1.80e-05	8.01
ITG	MOXD1	2.11	9.87	6.90e-08	1.24e-05	8.57
ITG	FEZF2	2.04	10.07	5.33e-08	1.01e-05	8.82
ITG	EGR3	2.40	10.43	3.40e-08	7.25e-06	9.27
ITG	TBR1	2.07	10.94	1.81e-08	4.33e-06	9.89
ITG	FRMPD2	2.13	11.49	9.36e-09	2.52e-06	10.54
ITG	HS3ST2	2.25	11.81	6.53e-09	1.99e-06	10.89
ITG	DLX1	2.1	12.	4.55e-	1.54e-	11.

© 2022 Williams JA et al. *JAMA Psychiatry*.

		4	13	09	06	24
ITG	RXFP1	2.18	12.11	4.63e-09	1.54e-06	11.23
ITG	C6orf142	2.32	12.43	3.27e-09	1.17e-06	11.57
ITG	LHX2	2.49	12.73	2.36e-09	9.43e-07	11.88
ITG	AC079341.1	2.34	13.03	1.73e-09	7.93e-07	12.18
ITG	SLC26A4	2.15	12.99	1.80e-09	7.93e-07	12.14
ITG	RPRML	2.04	13.23	1.40e-09	6.75e-07	12.38
ITG	GALNTL5	2.73	13.52	1.04e-09	5.37e-07	12.67
ITG	SATB2	2.14	13.68	8.81e-10	4.68e-07	12.83
ITG	OR14I1	2.58	13.73	8.38e-10	4.57e-07	12.87
ITG	NPTX2	2.11	13.81	7.79e-10	4.49e-07	12.94
ITG	KRT17	2.37	14.15	5.56e-10	3.46e-07	13.26
ITG	CREG2	2.30	14.22	5.20e-10	3.46e-07	13.33
ITG	AC078937.4	2.54	14.26	5.00e-10	3.45e-07	13.37
ITG	MET	2.71	14.58	3.67e-10	2.77e-07	13.66
ITG	ITPKA	2.15	14.67	3.36e-10	2.68e-07	13.74
ITG	KALRN	2.19	15.83	1.17e-10	1.09e-07	14.72
ITG	AC109486	2.0	16.	8.63e-	8.52e-	15.

© 2022 Williams JA et al. *JAMA Psychiatry*.

	.1	8	18	11	08	01
ITG	NRGN	2.81	16.44	6.89e-11	7.15e-08	15.21
ITG	NGEF	2.14	16.52	6.42e-11	7.01e-08	15.28
ITG	SLC17A7	2.15	16.67	5.67e-11	6.78e-08	15.39
ITG	KIAA0748	2.64	16.63	5.89e-11	6.78e-08	15.36
ITG	C6orf105	2.31	17.39	3.14e-11	4.07e-08	15.93
ITG	SLC22A9	2.31	17.48	2.90e-11	4.01e-08	16.00
ITG	ENC1	2.46	17.59	2.66e-11	3.95e-08	16.08
ITG	AC002563.2	2.52	17.71	2.42e-11	3.86e-08	16.16
ITG	HSPB3	2.80	17.96	1.98e-11	3.62e-08	16.34
ITG	FOXG1B	3.30	17.92	2.04e-11	3.62e-08	16.32
ITG	C1QL1	2.15	17.89	2.10e-11	3.62e-08	16.29
ITG	THEMIS	2.93	18.38	1.43e-11	3.29e-08	16.64
ITG	TRIM54	2.76	18.93	9.45e-12	2.75e-08	17.00
ITG	KCNV1	2.76	18.77	1.06e-11	2.75e-08	16.90
ITG	LY86-AS1	3.41	20.63	2.78e-12	9.59e-09	18.06
ITG	CCK	3.15	21.72	1.32e-12	5.49e-09	18.69
ITG	SLC30A3	2.9	22.	6.69e-	3.47e-	19.

© 2022 Williams JA et al. *JAMA Psychiatry*.

		9	78	13	09	26
ITG	GDA	3.12	23.64	3.92e-13	2.71e-09	19.69
ITG	PKD2L1	3.22	25.02	1.73e-13	1.80e-09	20.34
ITG	TMEM155	3.60	29.20	1.86e-14	3.85e-10	22.02

© 2022 Williams JA et al. *JAMA Psychiatry*.

eTable 10. Differential expression results in the MTG

Genes with a log-fold change >2 and a false discovery rate < .05 are shown.

BrainRegion	Gene	logFC	t	P.Value	adj.P.Val	B
MTG	ANXA8	2.10	6.27	1.45e-05	7.66e-04	3.17
MTG	NPPA	2.53	7.39	2.13e-06	1.59e-04	5.11
MTG	NPY	2.04	7.87	9.90e-07	9.04e-05	5.88
MTG	NEUROD6	2.18	7.90	9.51e-07	8.76e-05	5.92
MTG	LOC646627	2.43	8.71	2.77e-07	3.52e-05	7.16
MTG	FREM3	2.52	8.82	2.35e-07	3.06e-05	7.33
MTG	EGR3	2.21	9.12	1.54e-07	2.28e-05	7.75
MTG	CAMK2A	2.06	9.36	1.09e-07	1.80e-05	8.09
MTG	MOXD1	2.08	9.46	9.50e-08	1.64e-05	8.23
MTG	RXFP1	2.10	11.32	8.62e-09	2.55e-06	10.60
MTG	FRMPD2	2.19	11.54	6.62e-09	2.05e-06	10.86
MTG	C6orf142	2.25	11.60	6.21e-09	2.01e-06	10.92
MTG	TNNT2	2.33	12.05	3.65e-09	1.28e-06	11.44
MTG	LHX2	2.38	12.52	2.16e-09	8.61e-07	11.95
MTG	OR14I1	2.4	12.	1.40e-	6.07e-	12.

© 2022 Williams JA et al. *JAMA Psychiatry*.

		9	92	09	07	36
MTG	SLC26A4	2.06	12.91	1.41e-09	6.07e-07	12.36
MTG	AC079341.1	2.29	13.25	9.83e-10	4.74e-07	12.70
MTG	KRT17	2.27	13.33	8.99e-10	4.55e-07	12.79
MTG	CREG2	2.30	13.43	8.15e-10	4.33e-07	12.88
MTG	RPRML	2.08	13.53	7.30e-10	4.09e-07	12.98
MTG	GALNTL5	2.69	13.68	6.26e-10	3.71e-07	13.13
MTG	AC078937.4	2.48	13.71	6.08e-10	3.71e-07	13.16
MTG	DLX1	2.15	13.80	5.52e-10	3.47e-07	13.25
MTG	MET	2.68	13.89	5.06e-10	3.28e-07	13.33
MTG	SATB2	2.22	14.01	4.49e-10	3.10e-07	13.45
MTG	HS3ST2	2.26	13.97	4.63e-10	3.10e-07	13.42
MTG	ITPKA	2.09	14.43	2.96e-10	2.36e-07	13.84
MTG	ENC1	2.36	15.39	1.18e-10	1.11e-07	14.70
MTG	C6orf105	2.29	15.59	9.76e-11	1.05e-07	14.87
MTG	KALRN	2.20	15.74	8.54e-11	9.84e-08	15.00
MTG	SLC22A9	2.15	15.92	7.22e-11	8.81e-08	15.15
MTG	SLC17A7	2.0	16.	6.57e-	8.52e-	15.

© 2022 Williams JA et al. *JAMA Psychiatry*.

		5	03	11	08	24
MTG	NGEF	2.21	16.98	2.86e-11	3.96e-08	15.99
MTG	KIAA0748	2.71	17.09	2.62e-11	3.88e-08	16.07
MTG	KCNV1	2.68	17.25	2.29e-11	3.66e-08	16.19
MTG	NRGN	2.88	17.58	1.74e-11	3.15e-08	16.44
MTG	FOXG1B	3.27	17.52	1.82e-11	3.15e-08	16.40
MTG	HSPB3	2.78	18.35	9.34e-12	1.94e-08	17.00
MTG	AC002563.2	2.35	18.53	8.09e-12	1.86e-08	17.12
MTG	TRIM54	2.73	18.72	6.98e-12	1.81e-08	17.25
MTG	LY86-AS1	3.27	20.32	2.11e-12	6.26e-09	18.29
MTG	THEMIS	2.93	20.61	1.72e-12	5.94e-09	18.46
MTG	PKD2L1	3.19	23.69	2.21e-13	2.02e-09	20.15
MTG	GDA	3.07	23.03	3.35e-13	2.02e-09	19.82
MTG	CCK	3.17	22.70	4.13e-13	2.02e-09	19.65
MTG	SLC30A3	2.94	22.45	4.87e-13	2.02e-09	19.51
MTG	TMEM155	3.59	30.73	4.64e-15	9.62e-11	23.03

© 2022 Williams JA et al. *JAMA Psychiatry*.

eTable 11. Differential expression results in the PCu

Genes with a log-fold change >2 and a false discovery rate < .05 are shown.

BrainRegion	Gene	logFC	t	P.Value	adj.P.Val	B
PCu	HS3ST2	2.02	6.94	4.43e-06	3.89e-04	4.43
PCu	NEUROD6	2.18	7.44	1.94e-06	2.09e-04	5.25
PCu	C13orf16	2.08	8.19	5.98e-07	8.32e-05	6.43
PCu	EGR3	2.15	8.45	3.98e-07	6.10e-05	6.83
PCu	LHX2	2.25	8.77	2.51e-07	4.24e-05	7.28
PCu	PCSK1	2.03	9.11	1.52e-07	2.94e-05	7.78
PCu	FEZF2	2.03	10.02	4.35e-08	1.17e-05	9.01
PCu	RXFP1	2.15	10.13	3.79e-08	1.08e-05	9.14
PCu	MET	2.12	10.26	3.20e-08	9.90e-06	9.31
PCu	AC078937.4	2.14	10.87	1.47e-08	5.26e-06	10.06
PCu	TBR1	2.20	10.88	1.44e-08	5.25e-06	10.08
PCu	AC079341.1	2.41	11.01	1.23e-08	4.73e-06	10.23
PCu	ZBBX	2.08	11.09	1.12e-08	4.46e-06	10.33
PCu	KRT17	2.18	11.17	1.02e-08	4.18e-06	10.42
PCu	OR14I1	2.3	11.	5.57e-	2.75e-	10.

© 2022 Williams JA et al. *JAMA Psychiatry*.

		3	67	09	06	99
PCu	THEMIS	3.1 3	12. 16	3.14e-09	1.67e-06	11. 54
PCu	GALNTL5	2.5 6	12. 41	2.37e-09	1.41e-06	11. 81
PCu	ITPKA	2.0 1	12. 41	2.37e-09	1.41e-06	11. 80
PCu	KALRN	2.1 0	12. 57	2.00e-09	1.25e-06	11. 97
PCu	CREG2	2.0 8	12. 64	1.84e-09	1.22e-06	12. 05
PCu	ENC1	2.0 3	12. 79	1.56e-09	1.12e-06	12. 20
PCu	DLX1	2.1 1	13. 05	1.18e-09	9.45e-07	12. 46
PCu	AC002563 .2	2.0 4	13. 11	1.10e-09	9.16e-07	12. 52
PCu	RPRML	2.0 8	13. 54	7.07e-10	6.40e-07	12. 94
PCu	TNNT2	3.1 1	13. 91	4.80e-10	5.53e-07	13. 30
PCu	AC109486 .1	2.2 7	14. 25	3.43e-10	4.74e-07	13. 61
PCu	NGEF	2.2 5	14. 34	3.14e-10	4.64e-07	13. 69
PCu	CCK	2.9 4	14. 96	1.71e-10	2.72e-07	14. 24
PCu	SATB2	2.3 1	15. 23	1.32e-10	2.37e-07	14. 47
PCu	HSPB3	2.7 0	15. 19	1.37e-10	2.37e-07	14. 44
PCu	FOXG1B	3.2 2	15. 75	8.21e-11	1.70e-07	14. 90
PCu	KCNV1	2.6	16.	5.65e-	1.30e-	15.

© 2022 Williams JA et al. *JAMA Psychiatry*.

		2	16	11	07	23
PCu	TRIM54	2.80	16.58	3.91e-11	1.01e-07	15.56
PCu	NRGN	2.91	17.18	2.34e-11	6.92e-08	16.01
PCu	LY86-AS1	2.83	17.62	1.62e-11	5.59e-08	16.33
PCu	SLC30A3	2.63	18.93	5.72e-12	2.88e-08	17.21
PCu	KIAA0748	2.98	18.68	6.95e-12	2.88e-08	17.05
PCu	GDA	2.81	19.94	2.66e-12	2.10e-08	17.85
PCu	PKD2L1	2.77	19.76	3.04e-12	2.10e-08	17.74
PCu	TMEM155	3.66	22.93	3.42e-13	7.09e-09	19.48

© 2022 Williams JA et al. *JAMA Psychiatry*.

eTable 12. Differential expression results in the Pu

Genes with a log-fold change >2 and a false discovery rate < .05 are shown.

BrainRegion	Gene	logFC	t	P.Value	adj.P.Val	B
Pu	NPAS4	2.09	2.93	1.05e-02	4.80e-02	-3.84
Pu	RXRG	2.28	5.38	8.41e-05	1.03e-03	1.03
Pu	PTPN5	2.44	6.12	2.21e-05	3.55e-04	2.41
Pu	WIF1	2.77	7.02	4.74e-06	1.01e-04	4.00
Pu	KRTDAP	2.07	7.25	3.32e-06	7.62e-05	4.37
Pu	LOC727941	2.07	7.27	3.19e-06	7.37e-05	4.41
Pu	NOS1	2.16	7.35	2.80e-06	6.60e-05	4.54
Pu	KPRP	2.44	7.38	2.69e-06	6.36e-05	4.59
Pu	CACNA2D4	2.18	7.52	2.13e-06	5.27e-05	4.83
Pu	TMEM120B	2.11	7.81	1.36e-06	3.63e-05	5.29
Pu	TBC1D26	2.18	7.88	1.23e-06	3.38e-05	5.39
Pu	PASK	2.17	8.12	8.61e-07	2.53e-05	5.77
Pu	LINGO3	2.66	8.23	7.26e-07	2.19e-05	5.94
Pu	KCNIP2	2.24	8.26	6.98e-07	2.13e-05	5.98

© 2022 Williams JA et al. *JAMA Psychiatry*.

Pu	DSCR6	2.28	8.70	3.68e-07	1.26e-05	6.64
Pu	NTRK1	2.27	8.81	3.16e-07	1.12e-05	6.80
Pu	DGAT2	2.16	8.89	2.81e-07	1.02e-05	6.92
Pu	DNAH2	2.28	8.90	2.77e-07	1.01e-05	6.94
Pu	ISLR2	2.08	8.92	2.70e-07	9.91e-06	6.97
Pu	KL	2.13	9.09	2.12e-07	8.10e-06	7.21
Pu	DMKN	2.02	9.16	1.93e-07	7.48e-06	7.31
Pu	ECEL1	2.38	9.39	1.42e-07	5.73e-06	7.63
Pu	ENTPD3	2.42	9.44	1.32e-07	5.41e-06	7.70
Pu	UPB1	2.12	9.46	1.28e-07	5.29e-06	7.74
Pu	PRKCH	2.07	9.47	1.27e-07	5.25e-06	7.75
Pu	SYTL5	2.60	9.50	1.21e-07	5.05e-06	7.79
Pu	NPPB	2.00	9.71	9.19e-08	4.01e-06	8.08
Pu	KCNA4	2.13	9.79	8.29e-08	3.73e-06	8.19
Pu	WFDC2	2.00	9.86	7.62e-08	3.47e-06	8.27
Pu	OAF	2.13	10.11	5.51e-08	2.65e-06	8.61
Pu	FHDC1	2.81	10.17	5.09e-08	2.47e-06	8.69

© 2022 Williams JA et al. *JAMA Psychiatry*.

Pu	SCIN	2.69	10.26	4.56e-08	2.29e-06	8.80
Pu	CCDC37	2.10	10.29	4.40e-08	2.23e-06	8.84
Pu	HTR1D	2.62	10.32	4.22e-08	2.17e-06	8.88
Pu	RSPO4	2.24	10.45	3.57e-08	1.90e-06	9.05
Pu	C6ORF141	3.85	10.53	3.27e-08	1.79e-06	9.15
Pu	SCN4B	2.64	10.70	2.64e-08	1.50e-06	9.37
Pu	EGR4	2.76	10.70	2.63e-08	1.50e-06	9.37
Pu	EGR3	2.38	10.95	1.94e-08	1.16e-06	9.68
Pu	SH3RF2	3.29	11.03	1.77e-08	1.07e-06	9.78
Pu	KIAA0748	2.28	11.04	1.75e-08	1.06e-06	9.79
Pu	TDRD12	3.72	11.14	1.56e-08	9.62e-07	9.91
Pu	LOC729683	2.23	11.24	1.38e-08	8.67e-07	10.03
Pu	RGS8	2.73	11.26	1.35e-08	8.50e-07	10.06
Pu	ADAMTS19	2.12	11.30	1.28e-08	8.21e-07	10.11
Pu	C22orf31	2.03	11.32	1.26e-08	8.11e-07	10.13
Pu	NANOGP8	2.34	11.33	1.24e-08	8.04e-07	10.14
Pu	LRRC7	2.02	11.42	1.13e-08	7.46e-07	10.24

© 2022 Williams JA et al. *JAMA Psychiatry*.

Pu	TMEM45A	2.90	11.43	1.11e-08	7.42e-07	10.25
Pu	RGS20	2.51	11.54	9.77e-09	6.58e-07	10.39
Pu	LZTS1	2.11	11.55	9.62e-09	6.52e-07	10.40
Pu	NR1I3	2.03	11.59	9.19e-09	6.31e-07	10.45
Pu	TEX15	2.22	11.59	9.19e-09	6.31e-07	10.45
Pu	GNRH1	2.14	11.66	8.50e-09	5.93e-07	10.53
Pu	SYT6	2.11	11.76	7.61e-09	5.46e-07	10.64
Pu	CCDC39	2.87	11.82	7.12e-09	5.14e-07	10.71
Pu	KREMEN1	2.30	11.92	6.36e-09	4.72e-07	10.83
Pu	IL17RA	2.43	11.94	6.22e-09	4.64e-07	10.85
Pu	LRPAP1	2.03	12.07	5.38e-09	4.12e-07	11.00
Pu	PDYN	3.26	12.07	5.35e-09	4.11e-07	11.00
Pu	ADAMTS3	2.31	12.19	4.68e-09	3.65e-07	11.14
Pu	OBSCN	2.61	12.33	4.02e-09	3.24e-07	11.30
Pu	TBC1D10C	2.24	12.37	3.87e-09	3.17e-07	11.34
Pu	GPR52	2.13	12.43	3.61e-09	3.01e-07	11.41
Pu	AKAP5	2.00	12.49	3.40e-09	2.89e-07	11.47

© 2022 Williams JA et al. *JAMA Psychiatry*.

Pu	LOC100133686	2.27	12.56	3.14e-09	2.70e-07	11.55
Pu	SLC5A7	2.95	12.66	2.83e-09	2.49e-07	11.66
Pu	PDE7B	2.41	12.65	2.85e-09	2.49e-07	11.65
Pu	LINC00239	2.52	12.65	2.86e-09	2.49e-07	11.64
Pu	FOXO1	2.05	12.70	2.71e-09	2.40e-07	11.70
Pu	RARB	2.54	12.71	2.68e-09	2.38e-07	11.71
Pu	TK1	2.17	12.78	2.49e-09	2.25e-07	11.79
Pu	ACVR1C	2.04	12.79	2.47e-09	2.24e-07	11.80
Pu	AC130454.1	2.13	12.92	2.15e-09	1.98e-07	11.94
Pu	CALB1	2.30	12.97	2.04e-09	1.92e-07	11.99
Pu	PCP4L1	2.67	12.99	2.00e-09	1.90e-07	12.01
Pu	POU3F4	2.51	13.11	1.77e-09	1.75e-07	12.14
Pu	RYR3	2.12	13.14	1.72e-09	1.71e-07	12.17
Pu	PTPN7	2.27	13.23	1.56e-09	1.59e-07	12.26
Pu	DGKB	2.70	13.28	1.48e-09	1.53e-07	12.32
Pu	C19orf69	2.18	13.35	1.38e-09	1.45e-07	12.39
Pu	CRNDE	2.05	13.46	1.23e-09	1.32e-07	12.51

© 2022 Williams JA et al. *JAMA Psychiatry*.

Pu	DLX5	2.23	13.69	9.80e-10	1.10e-07	12.74
Pu	BCR	2.01	13.80	8.80e-10	1.00e-07	12.85
Pu	ITPKA	2.36	13.83	8.51e-10	9.86e-08	12.88
Pu	HPCA	2.86	13.88	8.13e-10	9.47e-08	12.93
Pu	FAM60A	2.53	13.95	7.62e-10	9.13e-08	12.99
Pu	GPR149	3.14	13.96	7.52e-10	9.07e-08	13.00
Pu	EPB41	2.36	14.03	7.05e-10	8.65e-08	13.07
Pu	SV2C	2.60	14.06	6.81e-10	8.45e-08	13.10
Pu	KLHL13	2.58	14.05	6.85e-10	8.45e-08	13.10
Pu	LOC283701	2.54	14.20	5.95e-10	7.66e-08	13.24
Pu	FBXL16	2.06	14.21	5.91e-10	7.66e-08	13.25
Pu	HTR4	3.43	14.37	5.05e-10	6.72e-08	13.41
Pu	SRGAP1	2.16	14.46	4.63e-10	6.21e-08	13.50
Pu	YPEL1	2.06	14.46	4.64e-10	6.21e-08	13.49
Pu	OTOF	2.08	14.54	4.31e-10	5.96e-08	13.57
Pu	INPP5A	2.35	14.67	3.79e-10	5.35e-08	13.70
Pu	SFTA3	2.69	14.78	3.43e-10	4.87e-08	13.80

© 2022 Williams JA et al. *JAMA Psychiatry*.

Pu	KLF5	2.48	14.82	3.31e-10	4.73e-08	13.84
Pu	KIAA1462	2.12	14.95	2.94e-10	4.35e-08	13.95
Pu	CFTR	2.51	14.98	2.85e-10	4.26e-08	13.98
Pu	EYA1	4.02	15.29	2.15e-10	3.25e-08	14.27
Pu	NPY	3.79	15.31	2.11e-10	3.21e-08	14.29
Pu	MEIS2	2.66	15.35	2.03e-10	3.14e-08	14.33
Pu	KCNK2	2.42	15.51	1.76e-10	2.78e-08	14.47
Pu	LPL	3.93	15.69	1.51e-10	2.49e-08	14.62
Pu	ADCY5	2.84	16.09	1.06e-10	1.84e-08	14.98
Pu	MOBKL2B	2.07	16.16	1.00e-10	1.78e-08	15.03
Pu	CORT	2.89	16.22	9.52e-11	1.72e-08	15.09
Pu	NRN1L	2.21	16.26	9.21e-11	1.68e-08	15.12
Pu	GNAL	2.76	16.32	8.75e-11	1.62e-08	15.17
Pu	APOC1	3.30	16.35	8.49e-11	1.62e-08	15.20
Pu	RASD2	3.81	16.34	8.58e-11	1.62e-08	15.19
Pu	ONECUT3	4.41	16.33	8.64e-11	1.62e-08	15.18
Pu	SLC32A1	2.40	16.33	8.66e-11	1.62e-08	15.18

© 2022 Williams JA et al. *JAMA Psychiatry*.

Pu	LOC150166	2.90	16.47	7.70e-11	1.54e-08	15.30
Pu	ARPP-21	2.92	16.65	6.62e-11	1.36e-08	15.45
Pu	NRGN	3.13	16.76	6.04e-11	1.26e-08	15.54
Pu	C20orf103	2.60	16.82	5.74e-11	1.21e-08	15.59
Pu	FOXG1B	3.28	17.01	4.89e-11	1.05e-08	15.75
Pu	PRKAG3	3.50	17.08	4.64e-11	1.01e-08	15.80
Pu	SLITRK6	3.81	17.26	4.01e-11	9.35e-09	15.95
Pu	ACTN2	2.09	17.36	3.68e-11	8.92e-09	16.03
Pu	PRKCB	2.53	17.36	3.70e-11	8.92e-09	16.03
Pu	BSPRY	3.48	17.71	2.78e-11	7.03e-09	16.31
Pu	DCLK3	2.67	17.91	2.37e-11	6.15e-09	16.47
Pu	RGS14	4.47	17.98	2.25e-11	6.04e-09	16.52
Pu	ANKRD43	2.74	17.96	2.28e-11	6.04e-09	16.51
Pu	HS3ST5	2.29	18.02	2.18e-11	5.94e-09	16.55
Pu	HTR2C	2.74	18.08	2.09e-11	5.77e-09	16.59
Pu	MN1	2.89	18.19	1.91e-11	5.35e-09	16.68
Pu	PDE1B	3.62	18.28	1.79e-11	5.16e-09	16.75

© 2022 Williams JA et al. *JAMA Psychiatry*.

Pu	KCTD17	2.19	18.26	1.82e-11	5.16e-09	16.73
Pu	TPBG	3.57	18.40	1.63e-11	4.91e-09	16.84
Pu	COCH	4.82	18.45	1.57e-11	4.84e-09	16.88
Pu	CPNE5	2.43	18.43	1.59e-11	4.84e-09	16.86
Pu	ST8SIA3	2.13	18.68	1.32e-11	4.14e-09	17.05
Pu	DACH1	2.72	18.70	1.30e-11	4.13e-09	17.06
Pu	KCNAB1	3.12	18.86	1.15e-11	3.80e-09	17.18
Pu	SLC35D3	3.49	18.86	1.16e-11	3.80e-09	17.18
Pu	COL25A1	3.07	18.96	1.07e-11	3.64e-09	17.25
Pu	TPD52L1	2.64	19.05	1.00e-11	3.52e-09	17.32
Pu	CCDC88C	4.10	19.11	9.58e-12	3.49e-09	17.36
Pu	ADRA2C	3.59	19.11	9.59e-12	3.49e-09	17.36
Pu	RPRML	3.04	19.55	6.97e-12	2.78e-09	17.67
Pu	KCNJ1	4.20	19.59	6.75e-12	2.75e-09	17.70
Pu	C14orf23	3.98	19.80	5.80e-12	2.51e-09	17.85
Pu	SMPD3	2.48	19.99	5.08e-12	2.40e-09	17.98
Pu	MYB	4.05	19.89	5.44e-12	2.40e-09	17.91

© 2022 Williams JA et al. *JAMA Psychiatry*.

Pu	RASGRP2	2.82	20.09	4.72e-12	2.33e-09	18.05
Pu	TESC	2.76	20.32	4.03e-12	2.09e-09	18.20
Pu	DDIT4L	3.59	20.96	2.59e-12	1.41e-09	18.63
Pu	FAM40B	3.57	21.57	1.73e-12	1.10e-09	19.02
Pu	PPP1R1A	3.08	21.55	1.75e-12	1.10e-09	19.01
Pu	SAG	4.66	22.27	1.10e-12	8.12e-10	19.45
Pu	ANKRD34B	4.27	22.36	1.03e-12	7.93e-10	19.51
Pu	BCL11B	3.56	22.53	9.29e-13	7.41e-10	19.61
Pu	NGEF	3.21	22.98	7.02e-13	6.07e-10	19.88
Pu	C1orf145	4.10	23.24	6.00e-13	5.65e-10	20.03
Pu	PENK	6.39	23.47	5.18e-13	5.12e-10	20.16
Pu	DLX6	2.98	23.56	4.92e-13	5.10e-10	20.22
Pu	ONECUT2	4.48	24.41	2.97e-13	3.42e-10	20.69
Pu	ADORA2A	5.05	24.66	2.56e-13	3.12e-10	20.83
Pu	PCP4	3.94	25.03	2.07e-13	2.86e-10	21.02
Pu	PDE10A	4.69	25.88	1.28e-13	2.04e-10	21.47
Pu	SIX3	4.85	27.25	6.13e-14	1.06e-10	22.14

© 2022 Williams JA et al. *JAMA Psychiatry*.

Pu	BATF	4.72	27.64	5.01e-14	9.44e-11	22.32
Pu	ANO3	5.94	27.82	4.55e-14	9.43e-11	22.41
Pu	CA12	5.85	28.16	3.82e-14	8.99e-11	22.57
Pu	PPP1R1B	4.42	28.12	3.90e-14	8.99e-11	22.55
Pu	TAC1	4.43	29.09	2.40e-14	7.10e-11	22.98
Pu	RGS9	5.75	30.04	1.51e-14	5.23e-11	23.39
Pu	AC005512.1	3.68	30.81	1.05e-14	4.36e-11	23.71
Pu	DRD2	4.98	32.27	5.38e-15	3.81e-11	24.29
Pu	GPR6	5.50	31.67	7.06e-15	3.81e-11	24.05
Pu	MME	5.46	31.58	7.34e-15	3.81e-11	24.02
Pu	TMEM90A	7.35	47.98	1.72e-17	3.57e-13	28.77

© 2022 Williams JA et al. *JAMA Psychiatry*.

eTable 13. Differential expression results in the SFG

Genes with a log-fold change >2 and a false discovery rate < .05 are shown.

BrainRegion	Gene	logFC	t	P.Value	adj.P.Val	B
SFG	NPY	2.15	7.15	3.66e-06	2.71e-04	4.59
SFG	LOC646627	2.48	7.25	3.12e-06	2.40e-04	4.75
SFG	C13orf16	2.08	8.07	8.65e-07	8.79e-05	6.04
SFG	NEUROD6	2.30	8.39	5.36e-07	6.08e-05	6.52
SFG	EGR3	2.09	8.80	2.95e-07	4.03e-05	7.12
SFG	FRMPD2	2.48	9.14	1.83e-07	2.79e-05	7.60
SFG	COL5A2	2.06	10.00	5.82e-08	1.15e-05	8.74
SFG	LHX6	2.04	10.31	3.87e-08	8.18e-06	9.14
SFG	MOXD1	2.04	10.36	3.66e-08	7.94e-06	9.20
SFG	FEZF2	2.14	10.89	1.89e-08	5.08e-06	9.85
SFG	SLN	2.71	10.97	1.73e-08	4.71e-06	9.94
SFG	CTXN3	2.09	11.25	1.23e-08	3.55e-06	10.27
SFG	GPR26	2.07	11.31	1.15e-08	3.47e-06	10.33
SFG	CREG2	2.16	11.43	9.94e-09	3.08e-06	10.48
SFG	PPEF1	2.0	11.	8.74e-	2.83e-	10.

© 2022 Williams JA et al. *JAMA Psychiatry*.

		4	54	09	06	60
SFG	OVOL2	2.14	11.73	7.06e-09	2.47e-06	10.81
SFG	LHX2	2.32	11.72	7.10e-09	2.47e-06	10.81
SFG	LOC339524	2.09	11.72	7.15e-09	2.47e-06	10.80
SFG	MCHR2	2.01	12.14	4.46e-09	1.78e-06	11.26
SFG	HS3ST2	2.26	12.53	2.89e-09	1.25e-06	11.68
SFG	GALNTL5	2.67	12.73	2.33e-09	1.05e-06	11.88
SFG	TNNT2	3.24	12.89	1.97e-09	9.24e-07	12.04
SFG	RXFP1	2.42	12.87	2.01e-09	9.24e-07	12.03
SFG	ENC1	2.07	12.93	1.88e-09	9.05e-07	12.09
SFG	SLC26A4	2.01	13.09	1.59e-09	7.85e-07	12.25
SFG	KRT17	2.35	13.35	1.22e-09	6.33e-07	12.50
SFG	DLX1	2.05	13.71	8.47e-10	5.02e-07	12.85
SFG	FREM3	3.01	13.78	7.85e-10	4.79e-07	12.92
SFG	OR14I1	2.57	14.11	5.67e-10	3.92e-07	13.23
SFG	C1QL1	2.09	14.23	5.05e-10	3.61e-07	13.34
SFG	KALRN	2.09	14.31	4.71e-10	3.49e-07	13.40
SFG	ANKRD56	2.0	14.	4.34e-	3.36e-	13.

© 2022 Williams JA et al. *JAMA Psychiatry*.

		4	39	10	07	48
SFG	SATB2	2.20	14.38	4.38e-10	3.36e-07	13.47
SFG	KCNV1	2.61	14.80	2.94e-10	2.44e-07	13.85
SFG	AC002563.2	2.29	15.14	2.14e-10	1.99e-07	14.14
SFG	AC079341.1	2.40	15.11	2.21e-10	1.99e-07	14.11
SFG	SLC17A7	2.01	15.25	1.93e-10	1.91e-07	14.24
SFG	NGEF	2.05	15.37	1.74e-10	1.80e-07	14.33
SFG	RTP1	2.03	16.32	7.51e-11	8.19e-08	15.11
SFG	AC109486.1	2.35	16.51	6.36e-11	7.37e-08	15.26
SFG	TRIM54	2.46	16.51	6.40e-11	7.37e-08	15.25
SFG	CBLN2	2.25	16.94	4.45e-11	5.77e-08	15.58
SFG	FOXG1B	3.25	17.26	3.41e-11	4.72e-08	15.82
SFG	NRGN	2.96	17.47	2.88e-11	4.26e-08	15.97
SFG	AC078937.4	2.62	18.00	1.89e-11	3.02e-08	16.35
SFG	MPPED1	2.04	18.52	1.26e-11	2.17e-08	16.71
SFG	C6orf105	2.36	18.71	1.09e-11	2.06e-08	16.83
SFG	LY86-AS1	3.09	18.94	9.15e-12	1.90e-08	16.98
SFG	GDA	3.1	19.	7.45e-	1.90e-	17.

© 2022 Williams JA et al. *JAMA Psychiatry*.

		1	22	12	08	16
SFG	PKD2L1	2.6<br>3	19.<br>08	8.23e-<br>12	1.90e-<br>08	17.<br>08
SFG	ASB2	2.1<br>4	19.<br>80	4.86e-<br>12	1.44e-<br>08	17.<br>53
SFG	THEMIS	3.1<br>4	20.<br>50	2.97e-<br>12	1.21e-<br>08	17.<br>95
SFG	SLC30A3	2.7<br>5	20.<br>27	3.49e-<br>12	1.21e-<br>08	17.<br>82
SFG	CCK	3.1<br>8	22.<br>25	9.15e-<br>13	7.99e-<br>09	18.<br>93
SFG	HSPB3	3.2<br>8	21.<br>71	1.31e-<br>12	7.99e-<br>09	18.<br>64
SFG	KIAA0748	3.0<br>6	21.<br>46	1.54e-<br>12	7.99e-<br>09	18.<br>50
SFG	TMEM155	3.7<br>2	32.<br>90	3.18e-<br>15	6.60e-<br>11	23.<br>10

© 2022 Williams JA et al. *JAMA Psychiatry*.

eTable 14. Differential expression results in the VeI_IV regions

Genes with a log-fold change >2 and a false discovery rate < .05 are shown.

BrainRegion	Gene	logFC	t	P.Value	adj.P.Val	B
VeI_IV	EPPK1	2.67	3.21	8.49e-03	3.54e-02	-3.38
VeI_IV	ESPNL	3.34	3.30	7.28e-03	3.15e-02	-3.22
VeI_IV	CLCNKB	4.24	3.67	3.81e-03	1.97e-02	-2.56
VeI_IV	RXFP4	2.01	3.68	3.74e-03	1.94e-02	-2.55
VeI_IV	TNF	2.71	3.69	3.66e-03	1.91e-02	-2.52
VeI_IV	SEPT12	2.07	3.79	3.10e-03	1.68e-02	-2.35
VeI_IV	PISD	2.08	3.82	2.95e-03	1.62e-02	-2.30
VeI_IV	GOLGA6C	2.04	3.85	2.79e-03	1.55e-02	-2.24
VeI_IV	C6ORF141	2.71	3.86	2.74e-03	1.53e-02	-2.23
VeI_IV	RP11-446H18.3	2.02	3.87	2.68e-03	1.51e-02	-2.20
VeI_IV	UNCX	2.45	4.06	1.96e-03	1.19e-02	-1.8

© 2022 Williams JA et al. *JAMA Psychiatry*.

						8
VeI_IV	CDH15	5.05	4.06	1.94e-03	1.18e-02	-1.87
VeI_IV	HPCAL1	2.10	4.45	1.03e-03	7.30e-03	-1.21
VeI_IV	COLEC11	2.24	4.60	8.04e-04	6.05e-03	-0.96
VeI_IV	RSPO1	2.00	4.61	7.88e-04	5.95e-03	-0.94
VeI_IV	FLJ41350	2.01	4.66	7.24e-04	5.56e-03	-0.85
VeI_IV	AC093849.1	3.52	4.70	6.80e-04	5.32e-03	-0.78
VeI_IV	AQP3	2.29	4.72	6.61e-04	5.20e-03	-0.75
VeI_IV	CHRNB3	2.36	4.91	4.93e-04	4.19e-03	-0.45
VeI_IV	PIP5KL1	2.78	5.07	3.80e-04	3.49e-03	-0.18
VeI_IV	LMNB1	2.03	5.10	3.65e-04	3.38e-03	-0.14
VeI_IV	ARVCF	2.35	5.11	3.60e-04	3.34e-03	-0.12
VeI_IV	C1orf150	3.77	5.50	1.98e-04	2.14e-03	0.51
VeI_IV	XKR7	2.2	5.5	1.78e-	1.98e-	0.6

© 2022 Williams JA et al. *JAMA Psychiatry*.

		9	7	04	03	1
VeI_IV	BHLHE22	2.46	5.59	1.75e-04	1.95e-03	0.63
VeI_IV	NXPH3	2.45	5.65	1.60e-04	1.82e-03	0.73
VeI_IV	DEFB1	2.84	5.69	1.51e-04	1.75e-03	0.79
VeI_IV	TTR	4.46	5.73	1.42e-04	1.67e-03	0.85
VeI_IV	RP1-74M1.1	3.01	5.80	1.28e-04	1.55e-03	0.96
VeI_IV	GUCA2B	2.40	5.86	1.18e-04	1.46e-03	1.05
VeI_IV	COL13A1	4.32	6.16	7.73e-05	1.05e-03	1.49
VeI_IV	CHRNA6	2.49	6.16	7.68e-05	1.04e-03	1.50
VeI_IV	MSX2	2.09	6.17	7.59e-05	1.04e-03	1.51
VeI_IV	C15orf27	3.44	6.30	6.31e-05	9.03e-04	1.70
VeI_IV	CEP76	2.21	6.34	5.99e-05	8.70e-04	1.76
VeI_IV	ARHGAP29	2.06	6.40	5.53e-05	8.15e-04	1.84
VeI_IV	SLC1A6	3.12	6.42	5.39e-05	8.00e-04	1.87
VeI_IV	GABRD	2.74	6.46	5.07e-05	7.63e-04	1.93
VeI_IV	LGALS12	2.11	6.54	4.59e-05	7.10e-04	2.04
VeI_IV	CNTD2	2.04	6.59	4.25e-05	6.71e-04	2.12
VeI_IV	UBASH3B	2.2	6.6	4.12e-	6.55e-	2.1

© 2022 Williams JA et al. *JAMA Psychiatry*.

		4	2	05	04	5
VeI_IV	SHMT1	2.07	6.67	3.86e-05	6.21e-04	2.22
VeI_IV	TEX14	2.43	6.68	3.79e-05	6.13e-04	2.24
VeI_IV	MEGF11	2.51	6.70	3.69e-05	5.99e-04	2.27
VeI_IV	RASGEF1C	2.01	6.73	3.57e-05	5.86e-04	2.30
VeI_IV	FAT2	3.91	6.75	3.47e-05	5.75e-04	2.33
VeI_IV	ADAMTS18	5.01	6.80	3.22e-05	5.46e-04	2.41
VeI_IV	HSPB7	2.56	6.82	3.13e-05	5.37e-04	2.44
VeI_IV	LOC728056	2.02	6.90	2.84e-05	4.97e-04	2.54
VeI_IV	SPINT1	2.95	6.94	2.70e-05	4.79e-04	2.59
VeI_IV	CA4	2.18	6.97	2.60e-05	4.65e-04	2.63
VeI_IV	CMTM7	2.32	6.99	2.53e-05	4.57e-04	2.66
VeI_IV	HIST1H3I	2.04	7.07	2.28e-05	4.22e-04	2.77
VeI_IV	LRRC38	3.30	7.12	2.15e-05	4.02e-04	2.83
VeI_IV	ZIC2	4.20	7.16	2.02e-05	3.84e-04	2.90
VeI_IV	CHGB	2.08	7.23	1.86e-05	3.62e-04	2.98
VeI_IV	GLI1	2.08	7.25	1.81e-05	3.53e-04	3.01
VeI_IV	AQP7	2.1	7.3	1.69e-	3.35e-	3.0

© 2022 Williams JA et al. *JAMA Psychiatry*.

		3	1	05	04	8
VeI_IV	ALOX5	3.45	7.31	1.68e-05	3.34e-04	3.09
VeI_IV	LAMC2	2.04	7.32	1.66e-05	3.32e-04	3.10
VeI_IV	MREG	2.43	7.33	1.65e-05	3.31e-04	3.11
VeI_IV	ZIC5	3.89	7.35	1.60e-05	3.25e-04	3.14
VeI_IV	TGM1	2.25	7.45	1.42e-05	2.96e-04	3.27
VeI_IV	NGFR	2.76	7.48	1.36e-05	2.86e-04	3.31
VeI_IV	NTF3	2.22	7.48	1.36e-05	2.85e-04	3.31
VeI_IV	STK10	2.40	7.52	1.29e-05	2.75e-04	3.36
VeI_IV	PCBP3	2.30	7.54	1.26e-05	2.71e-04	3.39
VeI_IV	TSPAN9	3.56	7.60	1.18e-05	2.59e-04	3.46
VeI_IV	AC021534.2	2.27	7.61	1.16e-05	2.56e-04	3.47
VeI_IV	ST18	2.17	7.63	1.14e-05	2.53e-04	3.49
VeI_IV	KRT33A	3.84	7.70	1.04e-05	2.38e-04	3.59
VeI_IV	CCNJL	3.75	7.74	9.98e-06	2.30e-04	3.64
VeI_IV	CST6	2.75	7.74	9.94e-06	2.30e-04	3.64
VeI_IV	TDRD6	2.24	7.76	9.75e-06	2.27e-04	3.66
VeI_IV	TRIM7	2.4	7.7	9.57e-	2.24e-	3.6

© 2022 Williams JA et al. *JAMA Psychiatry*.

		3	7	06	04	8
VeI_IV	GPRIN2	2.29	7.78	9.52e-06	2.24e-04	3.69
VeI_IV	DARC	2.11	7.82	9.10e-06	2.15e-04	3.73
VeI_IV	LCN8	2.54	7.87	8.50e-06	2.04e-04	3.80
VeI_IV	SLC16A9	2.42	7.92	8.06e-06	1.96e-04	3.86
VeI_IV	STON1	2.45	7.93	7.93e-06	1.94e-04	3.88
VeI_IV	KIAA0664L3	2.05	7.96	7.67e-06	1.89e-04	3.91
VeI_IV	GRM6	2.38	7.98	7.53e-06	1.86e-04	3.93
VeI_IV	FAM46A	2.01	8.01	7.25e-06	1.81e-04	3.97
VeI_IV	PLXNB2	2.00	8.02	7.19e-06	1.80e-04	3.98
VeI_IV	CHN2	2.21	8.03	7.04e-06	1.79e-04	4.00
VeI_IV	RP11-143E21.7	2.47	8.04	7.02e-06	1.78e-04	4.00
VeI_IV	C16orf86	2.10	8.04	6.96e-06	1.77e-04	4.01
VeI_IV	SLN	3.05	8.12	6.36e-06	1.65e-04	4.11
VeI_IV	SLC6A7	2.52	8.17	6.00e-06	1.58e-04	4.17
VeI_IV	LEMD1	2.49	8.18	5.96e-06	1.57e-04	4.17
VeI_IV	FUT1	2.06	8.19	5.86e-06	1.55e-04	4.19
VeI_IV	ELL3	2.8	8.2	5.40e-	1.46e-	4.2

© 2022 Williams JA et al. *JAMA Psychiatry*.

		5	6	06	04	8
VeI_IV	IL20RA	2.07	8.28	5.31e-06	1.45e-04	4.30
VeI_IV	PLA2R1	2.16	8.32	5.04e-06	1.40e-04	4.35
VeI_IV	AC019171.4	2.10	8.34	4.93e-06	1.38e-04	4.37
VeI_IV	CIDEB	2.10	8.35	4.91e-06	1.38e-04	4.38
VeI_IV	SLC26A2	2.00	8.35	4.89e-06	1.38e-04	4.38
VeI_IV	TFAP2A	2.36	8.37	4.81e-06	1.36e-04	4.40
VeI_IV	BARHL1	4.48	8.38	4.74e-06	1.35e-04	4.41
VeI_IV	CLK4	2.03	8.39	4.66e-06	1.33e-04	4.43
VeI_IV	AMDHD1	2.29	8.42	4.52e-06	1.30e-04	4.46
VeI_IV	ODZ1	2.14	8.43	4.46e-06	1.29e-04	4.48
VeI_IV	CDH23	2.50	8.44	4.40e-06	1.28e-04	4.49
VeI_IV	ANGPTL7	3.79	8.46	4.33e-06	1.26e-04	4.51
VeI_IV	TRIM67	2.60	8.49	4.18e-06	1.23e-04	4.55
VeI_IV	BDKRB1	2.35	8.49	4.16e-06	1.23e-04	4.55
VeI_IV	EEF1DP3	2.27	8.53	3.99e-06	1.20e-04	4.59
VeI_IV	SLC26A5	2.33	8.53	3.97e-06	1.20e-04	4.60
VeI_IV	PLK5P	2.5	8.5	3.72e-	1.14e-	4.6

© 2022 Williams JA et al. *JAMA Psychiatry*.

		1	9	06	04	7
VeI_IV	USP44	2.26	8.68	3.38e-06	1.06e-04	4.77
VeI_IV	FAM60A	2.10	8.75	3.13e-06	1.01e-04	4.85
VeI_IV	USP45	2.09	8.80	2.98e-06	9.66e-05	4.90
VeI_IV	C16orf79	2.16	8.80	2.98e-06	9.66e-05	4.90
VeI_IV	ARHGEF10L	2.00	8.81	2.94e-06	9.56e-05	4.91
VeI_IV	LOC283174	2.30	8.84	2.84e-06	9.34e-05	4.95
VeI_IV	GRID2IP	2.23	8.86	2.77e-06	9.17e-05	4.97
VeI_IV	LOC100292909	2.72	8.87	2.75e-06	9.12e-05	4.98
VeI_IV	STK17B	2.21	8.92	2.62e-06	8.77e-05	5.03
VeI_IV	PTCHD1	2.08	8.94	2.54e-06	8.58e-05	5.07
VeI_IV	FGF20	2.50	9.04	2.30e-06	7.99e-05	5.17
VeI_IV	RNF152	2.62	9.04	2.29e-06	7.98e-05	5.17
VeI_IV	RP11-561O23.6	2.05	9.06	2.25e-06	7.90e-05	5.19
VeI_IV	LGALS13	2.36	9.05	2.26e-06	7.90e-05	5.19
VeI_IV	ANK1	2.12	9.08	2.19e-06	7.80e-05	5.22
VeI_IV	ZNF702P	2.70	9.13	2.08e-06	7.56e-05	5.27
VeI_IV	KRT33B	3.4	9.1	2.01e-	7.43e-	5.3

© 2022 Williams JA et al. *JAMA Psychiatry*.

		2	6	06	05	1
VeI_IV	CCNG2	2.3<br>3	9.1<br>7	2.00e-<br>06	7.42e-<br>05	5.3<br>1
VeI_IV	AP000911.1	2.2<br>0	9.2<br>1	1.91e-<br>06	7.22e-<br>05	5.3<br>6
VeI_IV	FAM13A-AS1	2.2<br>7	9.2<br>7	1.79e-<br>06	6.83e-<br>05	5.4<br>3
VeI_IV	LINC00312	2.4<br>1	9.2<br>8	1.78e-<br>06	6.81e-<br>05	5.4<br>3
VeI_IV	GLCE	2.3<br>7	9.2<br>9	1.76e-<br>06	6.74e-<br>05	5.4<br>5
VeI_IV	POLB	2.1<br>0	9.2<br>9	1.75e-<br>06	6.74e-<br>05	5.4<br>5
VeI_IV	LINC00473	3.2<br>4	9.3<br>0	1.74e-<br>06	6.68e-<br>05	5.4<br>6
VeI_IV	P2RX4	2.0<br>2	9.3<br>6	1.64e-<br>06	6.34e-<br>05	5.5<br>2
VeI_IV	BANK1	2.2<br>5	9.4<br>1	1.56e-<br>06	6.16e-<br>05	5.5<br>7
VeI_IV	LIPG	2.8<br>0	9.4<br>2	1.54e-<br>06	6.14e-<br>05	5.5<br>9
VeI_IV	FGL2	2.3<br>8	9.4<br>2	1.54e-<br>06	6.14e-<br>05	5.5<br>9
VeI_IV	CBLB	2.2<br>6	9.4<br>2	1.54e-<br>06	6.14e-<br>05	5.5<br>8
VeI_IV	COL8A2	2.9<br>5	9.4<br>1	1.55e-<br>06	6.14e-<br>05	5.5<br>8
VeI_IV	CAMK4	2.1<br>3	9.5<br>1	1.40e-<br>06	5.67e-<br>05	5.6<br>8
VeI_IV	C5orf13	2.2<br>6	9.5<br>2	1.38e-<br>06	5.60e-<br>05	5.7<br>0
VeI_IV	RP11-<br>403C10.2	2.6<br>5	9.5<br>4	1.37e-<br>06	5.58e-<br>05	5.7<br>1
VeI_IV	SCGN	4.8	9.5	1.37e-	5.58e-	5.7

© 2022 Williams JA et al. *JAMA Psychiatry*.

		8	3	06	05	1
VeI_IV	RAB37	2.60	9.56	1.33e-06	5.49e-05	5.74
VeI_IV	MNS1	2.11	9.58	1.30e-06	5.37e-05	5.76
VeI_IV	KLF11	2.15	9.59	1.30e-06	5.37e-05	5.77
VeI_IV	DAND5	2.03	9.60	1.28e-06	5.30e-05	5.78
VeI_IV	RNF133	2.96	9.60	1.28e-06	5.30e-05	5.78
VeI_IV	C11orf52	3.22	9.62	1.25e-06	5.22e-05	5.80
VeI_IV	MPP4	2.03	9.65	1.22e-06	5.14e-05	5.83
VeI_IV	KIAA1024	2.08	9.65	1.22e-06	5.14e-05	5.83
VeI_IV	CPVL	2.27	9.69	1.16e-06	4.98e-05	5.88
VeI_IV	PVALB	3.55	9.73	1.12e-06	4.84e-05	5.92
VeI_IV	SPTBN5	3.32	9.73	1.12e-06	4.84e-05	5.92
VeI_IV	ECHDC2	2.08	9.75	1.10e-06	4.78e-05	5.94
VeI_IV	TRIM43	3.34	9.79	1.06e-06	4.66e-05	5.98
VeI_IV	ARSJ	2.41	9.79	1.05e-06	4.65e-05	5.98
VeI_IV	INADL	2.97	9.80	1.04e-06	4.63e-05	5.99
VeI_IV	PLCB4	2.17	9.81	1.03e-06	4.59e-05	6.00
VeI_IV	NUAK2	2.5	9.8	1.02e-	4.54e-	6.0

© 2022 Williams JA et al. *JAMA Psychiatry*.

		1	3	06	05	2
VeI_IV	MAMDC4	2.65	9.89	9.53e-07	4.32e-05	6.09
VeI_IV	SHROOM3	2.26	9.90	9.46e-07	4.30e-05	6.09
VeI_IV	NPTX1	2.10	9.97	8.87e-07	4.11e-05	6.16
VeI_IV	C20orf117	2.35	10.04	8.28e-07	3.90e-05	6.23
VeI_IV	WSCD2	2.49	10.04	8.21e-07	3.90e-05	6.24
VeI_IV	L1TD1	2.27	10.04	8.25e-07	3.90e-05	6.24
VeI_IV	RCAN3	2.23	10.06	8.12e-07	3.86e-05	6.25
VeI_IV	LDLRAP1	2.58	10.07	8.00e-07	3.83e-05	6.27
VeI_IV	CADPS2	3.00	10.08	7.94e-07	3.81e-05	6.27
VeI_IV	HIST1H1D	2.27	10.12	7.61e-07	3.69e-05	6.32
VeI_IV	CA9	3.37	10.13	7.56e-07	3.68e-05	6.33
VeI_IV	ESPN	2.02	10.13	7.53e-07	3.67e-05	6.33
VeI_IV	SFI1	2.06	10.18	7.16e-07	3.55e-05	6.38
VeI_IV	KRT24	2.52	10.21	7.01e-07	3.51e-05	6.40
VeI_IV	HPX	2.22	10.20	7.06e-07	3.51e-05	6.40
VeI_IV	DOK7	3.56	10.21	6.96e-07	3.50e-05	6.41
VeI_IV	APOO	2.1	10.	6.94e-	3.50e-	6.4

© 2022 Williams JA et al. *JAMA Psychiatry*.

		3	22	07	05	1
VeI_IV	CRNDE	2.55	10.30	6.38e-07	3.27e-05	6.50
VeI_IV	DUSP	2.91	10.34	6.16e-07	3.20e-05	6.54
VeI_IV	PCOLCE	2.38	10.40	5.85e-07	3.06e-05	6.59
VeI_IV	IRX2	2.60	10.47	5.47e-07	2.91e-05	6.66
VeI_IV	TLE2	2.76	10.47	5.47e-07	2.91e-05	6.66
VeI_IV	ALS2	2.25	10.48	5.41e-07	2.90e-05	6.67
VeI_IV	VIT	2.78	10.53	5.13e-07	2.78e-05	6.73
VeI_IV	STAC	2.94	10.58	4.93e-07	2.69e-05	6.77
VeI_IV	GNG13	3.15	10.60	4.84e-07	2.65e-05	6.79
VeI_IV	SPON2	2.59	10.64	4.66e-07	2.57e-05	6.83
VeI_IV	GRM1	2.18	10.73	4.27e-07	2.40e-05	6.92
VeI_IV	BDKRB2	2.99	10.76	4.14e-07	2.35e-05	6.95
VeI_IV	BSPRY	2.15	10.80	4.02e-07	2.32e-05	6.98
VeI_IV	FANCC	2.94	10.79	4.03e-07	2.32e-05	6.97
VeI_IV	FLT3	2.22	10.83	3.89e-07	2.27e-05	7.01
VeI_IV	SMPDL3B	2.87	10.89	3.67e-07	2.17e-05	7.07
VeI_IV	LOC1001313	3.7	10.	3.68e-	2.17e-	7.0

© 2022 Williams JA et al. *JAMA Psychiatry*.

	42	6	89	07	05	7
VeI_IV	LOC439914	2.71	10.94	3.52e-07	2.10e-05	7.11
VeI_IV	TNAP	3.28	10.99	3.36e-07	2.03e-05	7.16
VeI_IV	TMEM159	2.47	11.01	3.31e-07	2.02e-05	7.18
VeI_IV	PDZK1P1	3.77	11.11	3.01e-07	1.87e-05	7.28
VeI_IV	GRIN2C	3.08	11.16	2.89e-07	1.81e-05	7.32
VeI_IV	C1orf172	2.85	11.25	2.66e-07	1.73e-05	7.40
VeI_IV	DACT1	2.43	11.24	2.68e-07	1.73e-05	7.40
VeI_IV	SMCP	2.39	11.39	2.34e-07	1.55e-05	7.53
VeI_IV	CHRNA3	3.07	11.42	2.30e-07	1.54e-05	7.55
VeI_IV	PAG1	2.06	11.44	2.25e-07	1.53e-05	7.58
VeI_IV	KIAA1984	2.55	11.46	2.22e-07	1.51e-05	7.59
VeI_IV	SLC35F4	2.74	11.55	2.05e-07	1.43e-05	7.67
VeI_IV	LOC100287347	2.68	11.58	2.00e-07	1.40e-05	7.70
VeI_IV	DGKD	2.09	11.61	1.94e-07	1.37e-05	7.72
VeI_IV	CDON	2.40	11.64	1.89e-07	1.35e-05	7.75
VeI_IV	RELN	3.48	11.77	1.69e-07	1.25e-05	7.87
VeI_IV	ZFPM2	2.4	11.	1.59e-	1.18e-	7.9

© 2022 Williams JA et al. *JAMA Psychiatry*.

		3	84	07	05	3
VeI_IV	SPHKAP	2.13	11.86	1.56e-07	1.18e-05	7.95
VeI_IV	MSX2P1	2.73	11.86	1.57e-07	1.18e-05	7.94
VeI_IV	LHX1	3.20	11.88	1.54e-07	1.17e-05	7.96
VeI_IV	LOC388630	2.98	11.93	1.47e-07	1.13e-05	8.01
VeI_IV	GALNT12	2.33	12.00	1.39e-07	1.08e-05	8.07
VeI_IV	PAXIP1	2.71	12.02	1.36e-07	1.07e-05	8.09
VeI_IV	MMP24	2.44	12.03	1.36e-07	1.06e-05	8.09
VeI_IV	BTG1	2.59	12.05	1.34e-07	1.06e-05	8.11
VeI_IV	VSIG8	3.90	12.08	1.30e-07	1.04e-05	8.13
VeI_IV	SRGAP2	2.26	12.10	1.27e-07	1.03e-05	8.16
VeI_IV	PGAM2	3.03	12.11	1.27e-07	1.03e-05	8.16
VeI_IV	ROR1	3.06	12.12	1.26e-07	1.02e-05	8.17
VeI_IV	KRT19	4.26	12.17	1.21e-07	9.89e-06	8.21
VeI_IV	AP000926.2	2.60	12.21	1.17e-07	9.68e-06	8.24
VeI_IV	LPL	2.48	12.25	1.13e-07	9.48e-06	8.28
VeI_IV	MPP3	3.08	12.24	1.14e-07	9.48e-06	8.27
VeI_IV	TMEM61	3.3	12.	1.03e-	8.77e-	8.3

© 2022 Williams JA et al. *JAMA Psychiatry*.

		7	36	07	06	7
VeI_IV	CCDC88B	2.65	12.38	1.02e-07	8.69e-06	8.39
VeI_IV	ZNF300P1	2.55	12.46	9.52e-08	8.29e-06	8.45
VeI_IV	FUT2	2.91	12.55	8.80e-08	7.94e-06	8.53
VeI_IV	HS3ST1	2.82	12.67	8.03e-08	7.40e-06	8.63
VeI_IV	AC092436.3	2.81	12.76	7.49e-08	6.99e-06	8.70
VeI_IV	MEIS1	2.67	12.79	7.31e-08	6.86e-06	8.72
VeI_IV	DPF3	2.84	12.79	7.31e-08	6.86e-06	8.72
VeI_IV	FGF5	3.16	12.81	7.18e-08	6.80e-06	8.74
VeI_IV	AC068353.1	2.52	12.83	7.04e-08	6.69e-06	8.76
VeI_IV	USP3	2.62	12.88	6.77e-08	6.55e-06	8.80
VeI_IV	KIAA1456	3.57	13.03	6.04e-08	6.15e-06	8.92
VeI_IV	C19orf46	3.49	13.14	5.52e-08	5.89e-06	9.01
VeI_IV	ABLIM1	2.25	13.12	5.60e-08	5.89e-06	8.99
VeI_IV	ETV1	2.79	13.26	5.04e-08	5.44e-06	9.10
VeI_IV	GRM4	4.05	13.27	4.99e-08	5.42e-06	9.11
VeI_IV	SGCA	3.22	13.29	4.91e-08	5.39e-06	9.12
VeI_IV	KIAA0802	3.4	13.	4.60e-	5.13e-	9.1

		8	38	08	06	9
VeI_IV	SLC26A10	2.95	13.46	4.32e-08	4.87e-06	9.25
VeI_IV	CHRD	2.97	13.48	4.26e-08	4.83e-06	9.27
VeI_IV	CNTN6	2.26	13.63	3.81e-08	4.41e-06	9.38
VeI_IV	HOMER3	2.88	13.73	3.54e-08	4.17e-06	9.45
VeI_IV	TNFRSF25	3.14	13.90	3.11e-08	3.77e-06	9.59
VeI_IV	CRTAM	6.60	13.92	3.07e-08	3.77e-06	9.60
VeI_IV	IL28RA	2.85	14.02	2.85e-08	3.61e-06	9.67
VeI_IV	SKOR1	2.82	14.09	2.70e-08	3.50e-06	9.73
VeI_IV	LIMA1	3.18	14.14	2.61e-08	3.45e-06	9.76
VeI_IV	ATP2A3	4.45	14.15	2.59e-08	3.45e-06	9.77
VeI_IV	FSTL5	2.64	14.17	2.55e-08	3.42e-06	9.78
VeI_IV	ZP2	4.25	14.23	2.45e-08	3.34e-06	9.82
VeI_IV	CHD7	2.85	14.22	2.46e-08	3.34e-06	9.82
VeI_IV	EPB41	3.07	14.21	2.48e-08	3.34e-06	9.81
VeI_IV	C18orf42	3.86	14.27	2.37e-08	3.30e-06	9.86
VeI_IV	MYO5C	2.76	14.30	2.32e-08	3.27e-06	9.88
VeI_IV	TSPAN18	3.4	14.	2.21e-	3.17e-	9.9

© 2022 Williams JA et al. *JAMA Psychiatry*.

		2	37	08	06	2
VeI_IV	PAX6	3.76	14.39	2.18e-08	3.15e-06	9.94
VeI_IV	SHF	3.19	14.57	1.92e-08	2.94e-06	10.07
VeI_IV	KRT31	4.46	14.56	1.93e-08	2.94e-06	10.06
VeI_IV	EBF1	2.59	14.56	1.93e-08	2.94e-06	10.06
VeI_IV	LRCH1	2.78	14.65	1.81e-08	2.86e-06	10.13
VeI_IV	C4orf49	2.75	14.72	1.72e-08	2.77e-06	10.18
VeI_IV	ADAMTS16	3.40	14.73	1.71e-08	2.77e-06	10.18
VeI_IV	NEUROD2	3.39	14.94	1.48e-08	2.55e-06	10.33
VeI_IV	PAX3	2.88	15.02	1.40e-08	2.48e-06	10.38
VeI_IV	KCNJ12	2.90	15.01	1.41e-08	2.48e-06	10.37
VeI_IV	PDZK1	4.91	15.11	1.31e-08	2.39e-06	10.45
VeI_IV	RNF148	3.04	15.26	1.19e-08	2.22e-06	10.54
VeI_IV	TIMP4	4.25	15.38	1.10e-08	2.08e-06	10.62
VeI_IV	IL16	5.12	15.63	9.23e-09	1.86e-06	10.79
VeI_IV	GPRC5C	3.08	15.88	7.87e-09	1.72e-06	10.95
VeI_IV	ZIC4	4.91	15.86	7.95e-09	1.72e-06	10.94
VeI_IV	WFIKKN2	2.6	15.	8.09e-	1.72e-	10.

© 2022 Williams JA et al. *JAMA Psychiatry*.

		7	83	09	06	92
VeI_IV	GCOM1	3.5 3	15. 82	8.15e- 09	1.72e- 06	10. 92
VeI_IV	MYT1	2.9 2	16. 04	7.10e- 09	1.65e- 06	11. 05
VeI_IV	ZNF521	3.0 3	16. 32	5.90e- 09	1.42e- 06	11. 23
VeI_IV	C9orf171	4.1 8	16. 66	4.77e- 09	1.22e- 06	11. 44
VeI_IV	ENO3	3.7 0	16. 68	4.69e- 09	1.22e- 06	11. 46
VeI_IV	TLL1	3.5 0	16. 73	4.55e- 09	1.21e- 06	11. 49
VeI_IV	ZNF556	3.8 4	16. 83	4.27e- 09	1.15e- 06	11. 55
VeI_IV	THRSP	3.6 1	17. 12	3.58e- 09	1.00e- 06	11. 72
VeI_IV	CA8	3.5 0	17. 23	3.35e- 09	9.83e- 07	11. 78
VeI_IV	RNF122	3.2 5	17. 23	3.35e- 09	9.83e- 07	11. 78
VeI_IV	PRSS22	3.2 8	17. 20	3.41e- 09	9.83e- 07	11. 76
VeI_IV	ADAMTS5	3.2 7	17. 44	2.96e- 09	9.55e- 07	11. 90
VeI_IV	C7orf16	5.1 2	17. 40	3.02e- 09	9.55e- 07	11. 88
VeI_IV	CALB1	3.3 5	17. 48	2.88e- 09	9.48e- 07	11. 93
VeI_IV	SYTL1	4.6 1	18. 23	1.85e- 09	6.87e- 07	12. 35
VeI_IV	IRF6	4.4 8	18. 30	1.79e- 09	6.76e- 07	12. 38
VeI_IV	CNPY1	4.1	18.	1.79e-	6.76e-	12.

© 2022 Williams JA et al. *JAMA Psychiatry*.

		8	29	09	07	38
VeI_IV	ZNF663	4.94	18.46	1.63e-09	6.51e-07	12.47
VeI_IV	GRID2	3.06	18.72	1.41e-09	6.07e-07	12.62
VeI_IV	MAB21L1	4.25	19.01	1.20e-09	5.63e-07	12.77
VeI_IV	AMICA1	5.03	18.96	1.23e-09	5.63e-07	12.74
VeI_IV	PAX2	3.81	19.12	1.13e-09	5.43e-07	12.82
VeI_IV	PKIB	3.68	19.87	7.51e-10	3.80e-07	13.21
VeI_IV	MDGA1	3.93	20.08	6.72e-10	3.57e-07	13.31
VeI_IV	LOC100287005	3.65	20.42	5.64e-10	3.39e-07	13.47
VeI_IV	NRK	4.71	20.29	6.03e-10	3.39e-07	13.41
VeI_IV	ATP4A	5.62	20.28	6.05e-10	3.39e-07	13.41
VeI_IV	ZIC3	4.09	21.41	3.41e-10	2.53e-07	13.93
VeI_IV	CERKL	4.73	21.68	3.00e-10	2.49e-07	14.05
VeI_IV	CBLN3	6.13	22.05	2.50e-10	2.25e-07	14.22
VeI_IV	EXPH5	4.44	23.14	1.50e-10	1.64e-07	14.68
VeI_IV	DAO	6.17	23.87	1.08e-10	1.32e-07	14.97
VeI_IV	NEUROD1	6.65	25.44	5.48e-11	8.68e-08	15.56
VeI_IV	BARHL2	5.5	25.	5.86e-	8.68e-	15.

© 2022 Williams JA et al. *JAMA Psychiatry*.

		5	28	11	08	50
VeI_IV	ZIC1	5.62	26.00	4.36e-11	7.53e-08	15.75
VeI_IV	CPLX4	4.89	26.42	3.67e-11	6.91e-08	15.90
VeI_IV	CBLN1	5.69	28.13	1.88e-11	6.10e-08	16.46
VeI_IV	SPINK6	5.02	27.17	2.73e-11	6.10e-08	16.15
VeI_IV	RHBG	6.92	26.99	2.92e-11	6.10e-08	16.09
VeI_IV	PCP2	7.00	29.56	1.11e-11	5.74e-08	16.89
VeI_IV	GABRA6	7.25	31.74	5.18e-12	3.58e-08	17.48
VeI_IV	TFAP2B	5.72	31.73	5.19e-12	3.58e-08	17.48
VeI_IV	EOMES	5.99	36.12	1.30e-12	2.69e-08	18.51

© 2022 Williams JA et al. *JAMA Psychiatry*.

eTable 15. Biomedical ontology gene enrichment results

Figure Legend: Genes differentially overexpressed (log fold change > 2, false discovery rate p-value < 0.05) in each brain region (BrianROI) were enriched for known associations in the Disease Ontology (DO) and the biological process domain of the Gene Ontology (GOBP). Mouse orthologs of genes were also enriched in the Mammalian Phenotype (MP) ontology. Adjusted p-value of < 0.05 (Bonferroni corrected) shown.

© 2022 Williams JA et al. *JAMA Psychiatry*.

BrainROI	Ontology	Term	TermName	FoldChange	Zscore	Pvalue
fro	MP	MP:0000788	abnormal cerebral cortex morphology	7.73	6.00	4.09e-02
fro	MP	MP:0000801	abnormal temporal lobe morphology	8.02	6.14	3.35e-02
fro	MP	MP:0000807	abnormal hippocampus morphology	8.16	6.20	3.02e-02
FuG	DO	DOID:150	disease of mental health	4.22	5.85	4.50e-03
FuG	MP	MP:0004166	abnormal limbic system morphology	7.16	6.16	2.04e-02
FuG	MP	MP:0003635	abnormal synaptic transmission	5.43	6.14	6.13e-03
FuG	MP	MP:0014114	abnormal cognition	6.11	6.66	1.92e-03
FuG	MP	MP:0002063	abnormal learning/memory/conditioning	6.12	6.67	1.88e-03
FuG	MP	MP:0002206	abnormal CNS synaptic transmission	6.16	6.70	1.76e-03
FuG	MP	MP:0009538	abnormal synapse morphology	12.80	8.12	1.63e-03
FuG	MP	MP:0002912	abnormal excitatory postsynaptic potential	18.70	9.18	1.06e-03

© 2022 Williams JA et al. *JAMA Psychiatry*.

FuG	MP	MP:0002207	abnormal long term potentiation	11.90	8.42	4.50e -04
ITG	DO	DOID:150	disease of mental health	3.90	5.26	2.74e -02
ITG	MP	MP:0003635	abnormal synaptic transmission	4.89	5.39	4.50e -02
ITG	MP	MP:0002882	abnormal neuron morphology	3.47	4.99	4.50e -02
ITG	MP	MP:0002062	abnormal associative learning	8.40	6.31	2.66e -02
ITG	MP	MP:0002063	abnormal learning/memory/conditioning	5.51	5.87	1.59e -02
ITG	MP	MP:0014114	abnormal cognition	5.50	5.87	1.59e -02
ITG	MP	MP:0002206	abnormal CNS synaptic transmission	5.54	5.90	1.51e -02
ITG	MP	MP:0002752	abnormal somatic nervous system morphology	4.74	5.85	8.58e -03
ITG	MP	MP:0002207	abnormal long term potentiation	10.20	7.11	7.36e -03
ITG	MP	MP:0004166	abnormal limbic system morphology	8.19	7.19	1.84e -03
ITG	MP	MP:0009538	abnormal synapse morphology	12.80	8.12	1.63e -03

© 2022 Williams JA et al. *JAMA Psychiatry*.

ITG	MP	MP:0002912	abnormal excitatory postsynaptic potential	18.70	9.18	1.06e-03
MTG	DO	DOID:9975	cocaine dependence	7.02	5.72	3.72e-02
MTG	DO	DOID:0050701	electroclinical syndrome	4.46	5.33	3.64e-02
MTG	DO	DOID:0050702	neonatal period electroclinical syndrome	5.55	5.62	2.74e-02
MTG	DO	DOID:2234	focal epilepsy	4.20	5.79	6.54e-03
MTG	DO	DOID:331	central nervous system disease	1.59	5.00	4.90e-03
MTG	DO	DOID:3328	temporal lobe epilepsy	4.68	6.07	3.64e-03
MTG	DO	DOID:936	brain disease	2.24	6.16	8.99e-05
MTG	DO	DOID:0060041	autism spectrum disorder	3.09	6.83	2.17e-05
MTG	DO	DOID:0060037	developmental disorder of mental health	2.40	6.55	1.84e-05
MTG	DO	DOID:12849	autistic disorder	3.17	7.01	1.10e-05
MTG	DO	DOID:0060040	pervasive developmental disorder	3.22	7.44	1.47e-06

© 2022 Williams JA et al. *JAMA Psychiatry*.

MTG	DO	DOID:2468	psychotic disorder	2.17	7.06	6.54e-07
MTG	DO	DOID:5419	schizophrenia	2.18	7.10	5.72e-07
MTG	DO	DOID:1561	cognitive disorder	2.11	7.56	2.82e-08
MTG	DO	DOID:1826	epilepsy	3.80	9.27	3.23e-10
MTG	DO	DOID:150	disease of mental health	1.98	8.41	5.72e-11
MTG	GOBP	GO:0007155	cell adhesion	1.91	4.58	4.90e-02
MTG	GOBP	GO:0035556	intracellular signal transduction	2.00	4.65	4.09e-02
MTG	GOBP	GO:0050919	negative chemotaxis	7.49	5.97	2.17e-02
MTG	GOBP	GO:0003407	neural retina development	8.02	6.25	1.23e-02
MTG	GOBP	GO:0017158	regulation of calcium ion-dependent exocytosis	5.44	5.87	1.19e-02
MTG	GOBP	GO:0008306	associative learning	6.24	6.10	8.99e-03
MTG	GOBP	GO:0050796	regulation of insulin secretion	4.03	5.82	4.90e-03

© 2022 Williams JA et al. *JAMA Psychiatry*.

MTG	GOBP	GO:0007215	glutamate receptor signaling pathway	8.74	7.12	1.23e-03
MTG	GOBP	GO:0007218	neuropeptide signaling pathway	3.56	6.09	1.10e-03
MTG	GOBP	GO:0048167	regulation of synaptic plasticity	6.24	6.82	8.17e-04
MTG	GOBP	GO:0007165	signal transduction	1.68	5.46	6.54e-04
MTG	GOBP	GO:0048791	calcium ion-regulated exocytosis of neurotransmitter	6.06	7.01	3.80e-04
MTG	GOBP	GO:0006813	potassium ion transport	4.13	6.55	3.60e-04
MTG	GOBP	GO:0051965	positive regulation of synapse assembly	4.76	6.87	2.00e-04
MTG	GOBP	GO:0034765	regulation of ion transmembrane transport	3.58	6.60	1.43e-04
MTG	GOBP	GO:0007613	memory	5.08	7.45	2.78e-05
MTG	GOBP	GO:0007399	nervous system development	2.77	7.40	6.13e-07
MTG	GOBP	GO:0071805	potassium ion transmembrane transport	4.23	8.57	4.50e-08
MTG	GOBP	GO:0007268	chemical synaptic transmission	4.19	11.00	1.96e-14

© 2022 Williams JA et al. *JAMA Psychiatry*.

MTG	MP	MP:0001982	decreased chemically-elicited antinociception	5.96	5.53	4.50e -02
MTG	MP	MP:0004769	abnormal synaptic vesicle morphology	3.81	5.13	4.50e -02
MTG	MP	MP:0002873	normal phenotype	1.41	4.40	4.50e -02
MTG	MP	MP:0002068	abnormal parental behavior	2.56	4.84	4.09e -02
MTG	MP	MP:0000788	abnormal cerebral cortex morphology	2.02	4.66	4.09e -02
MTG	MP	MP:0008415	abnormal neurite morphology	1.95	4.65	3.96e -02
MTG	MP	MP:0002169	no abnormal phenotype detected	1.41	4.43	3.84e -02
MTG	MP	MP:0006007	abnormal basal ganglion morphology	2.87	4.96	3.64e -02
MTG	MP	MP:0002578	impaired ability to fire action potentials	6.24	5.71	2.86e -02
MTG	MP	MP:0005660	abnormal circulating adrenaline level	6.24	5.71	2.86e -02
MTG	MP	MP:0012315	impaired learning	3.59	5.27	2.37e -02
MTG	MP	MP:0002752	abnormal somatic nervous system morphology	1.63	4.65	2.33e -02

© 2022 Williams JA et al. *JAMA Psychiatry*.

MTG	MP	MP:0000947	convulsive seizures	2.60	5.01	2.25e-02
MTG	MP	MP:0000951	sporadic seizures	5.11	5.61	2.21e-02
MTG	MP	MP:0000814	absent dentate gyrus	7.49	5.97	2.17e-02
MTG	MP	MP:0008262	abnormal hippocampus region morphology	3.86	5.41	1.84e-02
MTG	MP	MP:0004077	abnormal striatum morphology	3.37	5.31	1.68e-02
MTG	MP	MP:0010698	abnormal impulsive behavior control	9.36	6.28	1.63e-02
MTG	MP	MP:0008143	abnormal dendrite morphology	2.55	5.11	1.47e-02
MTG	MP	MP:0005402	abnormal action potential	2.97	5.28	1.27e-02
MTG	MP	MP:0003461	abnormal response to novel object	4.06	5.63	9.81e-03
MTG	MP	MP:0003997	tonic-clonic seizures	3.70	5.59	8.17e-03
MTG	MP	MP:0008414	abnormal spatial reference memory	4.03	5.82	4.90e-03
MTG	MP	MP:0000807	abnormal hippocampus morphology	2.19	5.28	4.50e-03

© 2022 Williams JA et al. *JAMA Psychiatry*.

MTG	MP	MP:0012348	decreased susceptibility to induction of seizure by inducing agent	6.02	6.31	4.01e-03
MTG	MP	MP:0002887	decreased susceptibility to pharmacologically induced seizures	6.02	6.31	4.01e-03
MTG	MP	MP:0001462	abnormal avoidance learning behavior	3.74	5.84	3.51e-03
MTG	MP	MP:0004166	abnormal limbic system morphology	2.13	5.33	3.23e-03
MTG	MP	MP:0000794	abnormal parietal lobe morphology	5.15	6.24	2.86e-03
MTG	MP	MP:0000801	abnormal temporal lobe morphology	2.21	5.39	2.78e-03
MTG	MP	MP:0002886	abnormal glutamate-mediated receptor currents	4.23	6.05	2.66e-03
MTG	MP	MP:0003412	abnormal afterhyperpolarization	8.19	6.84	2.29e-03
MTG	MP	MP:0002557	abnormal social/conspecific interaction	2.27	5.66	9.81e-04
MTG	MP	MP:0001970	abnormal pain threshold	2.59	5.86	7.36e-04
MTG	MP	MP:0001650	abnormal seizure response to electrical stimulation	6.46	6.99	5.72e-04
MTG	MP	MP:0003989	abnormal barrel cortex morphology	8.32	7.38	4.50e-04

© 2022 Williams JA et al. *JAMA Psychiatry*.

MTG	MP	MP:0001431	abnormal eating behavior	2.00	5.70	4.50e -04
MTG	MP	MP:0002069	abnormal consumption behavior	1.93	5.68	4.50e -04
MTG	MP	MP:0004994	abnormal brain wave pattern	4.41	6.68	2.82e -04
MTG	MP	MP:0000859	abnormal somatosensory cortex morphology	6.24	7.16	2.57e -04
MTG	MP	MP:0009454	impaired contextual conditioning behavior	4.68	6.78	2.57e -04
MTG	MP	MP:0002918	abnormal paired-pulse facilitation	4.24	6.69	2.33e -04
MTG	MP	MP:0002917	decreased synaptic depression	8.81	7.65	2.29e -04
MTG	MP	MP:0008540	abnormal cerebral hemisphere morphology	1.99	5.85	2.17e -04
MTG	MP	MP:0000783	abnormal forebrain morphology	1.74	5.72	2.17e -04
MTG	MP	MP:0009456	impaired cued conditioning behavior	5.41	7.03	2.00e -04
MTG	MP	MP:0001364	decreased anxiety-related response	3.25	6.44	1.72e -04
MTG	MP	MP:0001458	abnormal object recognition memory	4.27	6.91	9.81e -05

© 2022 Williams JA et al. *JAMA Psychiatry*.

MTG	MP	MP:0005449	abnormal food intake	2.24	6.16	8.99e-05
MTG	MP	MP:0002736	abnormal nociception after inflammation	5.80	7.39	7.36e-05
MTG	MP	MP:0001475	reduced long term depression	5.80	7.39	7.36e-05
MTG	MP	MP:0000860	abnormal primary somatosensory cortex morphology	8.87	8.15	4.50e-05
MTG	MP	MP:0003360	abnormal depression-related behavior	3.74	7.01	2.98e-05
MTG	MP	MP:0001413	abnormal response to new environment	2.89	6.71	2.62e-05
MTG	MP	MP:0002152	abnormal brain morphology	1.61	6.06	2.45e-05
MTG	MP	MP:0012349	increased susceptibility to induction of seizure by inducing agent	3.92	7.13	2.29e-05
MTG	MP	MP:0002906	increased susceptibility to pharmacologically induced seizures	3.96	7.19	1.88e-05
MTG	MP	MP:0000787	abnormal telencephalon morphology	1.92	6.31	1.76e-05
MTG	MP	MP:0009747	impaired behavioral response to xenobiotic	4.41	7.48	1.06e-05
MTG	MP	MP:0004811	abnormal neuron physiology	2.06	6.53	8.17e-06

© 2022 Williams JA et al. *JAMA Psychiatry*.

MTG	MP	MP:0013189	abnormal exploration in a new environment	3.24	7.16	6.13e -06
MTG	MP	MP:0001516	abnormal motor coordination/ balance	1.96	6.64	3.31e -06
MTG	MP	MP:0003107	abnormal response to novelty	2.80	7.14	2.62e -06
MTG	MP	MP:0001968	abnormal touch/ nociception	2.73	7.11	2.49e -06
MTG	MP	MP:0002882	abnormal neuron morphology	1.69	6.59	1.63e -06
MTG	MP	MP:0001417	decreased exploration in new environment	4.31	7.88	1.47e -06
MTG	MP	MP:0001405	impaired coordination	2.43	7.19	7.36e -07
MTG	MP	MP:0001898	abnormal long term depression	4.58	8.27	3.68e -07
MTG	MP	MP:0004859	abnormal synaptic plasticity	5.47	8.58	3.43e -07
MTG	MP	MP:0003008	enhanced long term potentiation	5.56	8.68	2.53e -07
MTG	MP	MP:0012317	impaired conditioning behavior	4.50	8.50	1.02e -07
MTG	MP	MP:0001363	increased anxiety-related response	3.39	8.04	9.81e -08

© 2022 Williams JA et al. *JAMA Psychiatry*.

MTG	MP	MP:0001473	reduced long term potentiation	3.82	8.29	7.36e-08
MTG	MP	MP:0003492	abnormal involuntary movement	2.00	7.40	4.90e-08
MTG	MP	MP:0002272	abnormal nervous system electrophysiology	2.81	7.92	3.92e-08
MTG	MP	MP:0002945	abnormal inhibitory postsynaptic currents	5.07	8.93	3.56e-08
MTG	MP	MP:0000950	abnormal seizure response to pharmacological agent	4.15	8.58	3.39e-08
MTG	MP	MP:0003491	abnormal voluntary movement	1.76	7.60	4.09e-09
MTG	MP	MP:0002910	abnormal excitatory postsynaptic currents	4.27	9.09	3.11e-09
MTG	MP	MP:0003313	abnormal locomotor activation	1.96	7.88	1.80e-09
MTG	MP	MP:0001961	abnormal reflex	2.23	8.17	1.02e-09
MTG	MP	MP:0001392	abnormal locomotor behavior	1.83	7.94	6.13e-10
MTG	MP	MP:0002912	abnormal excitatory postsynaptic potential	4.95	9.68	5.72e-10
MTG	MP	MP:0002915	abnormal synaptic depression	4.57	9.59	4.05e-10

© 2022 Williams JA et al. *JAMA Psychiatry*.

MTG	MP	MP:0001469	abnormal contextual conditioning behavior	4.35	9.52	3.43e -10
MTG	MP	MP:0001468	abnormal temporal memory	4.08	9.57	1.27e -10
MTG	MP	MP:0001463	abnormal spatial learning	3.57	9.32	1.19e -10
MTG	MP	MP:0001454	abnormal cued conditioning behavior	5.02	10.1 0	6.13e -11
MTG	MP	MP:0002064	seizures	2.98	9.23	2.74e -11
MTG	MP	MP:0001399	hyperactivity	2.86	9.56	1.76e -12
MTG	MP	MP:0009745	abnormal behavioral response to xenobiotic	3.49	10.1 0	5.31e -13
MTG	MP	MP:0004753	abnormal miniature excitatory postsynaptic currents	5.69	11.3 0	4.05e -13
MTG	MP	MP:0001362	abnormal anxiety-related response	3.35	10.1 0	2.98e -13
MTG	MP	MP:0009357	abnormal seizure response to inducing agent	4.33	10.8 0	1.23e -13
MTG	MP	MP:0002065	abnormal fear/anxiety-related behavior	3.28	10.4 0	4.90e -14
MTG	MP	MP:0002207	abnormal long term potentiation	4.02	11.0 0	1.27e -14

© 2022 Williams JA et al. *JAMA Psychiatry*.

MTG	MP	MP:0002067	abnormal sensory capabilities/reflexes/nociception	2.36	10.00	4.50e-15
MTG	MP	MP:0002062	abnormal associative learning	3.90	11.70	6.95e-17
MTG	MP	MP:0002572	abnormal emotion/affect behavior	2.85	11.00	5.72e-17
MTG	MP	MP:0003633	abnormal nervous system physiology	2.12	12.10	3.15e-24
MTG	MP	MP:0014114	abnormal cognition	3.21	13.60	1.51e-25
MTG	MP	MP:0002063	abnormal learning/memory/conditioning	3.21	13.60	1.31e-25
MTG	MP	MP:0003635	abnormal synaptic transmission	3.11	13.80	8.99e-27
MTG	MP	MP:0002206	abnormal CNS synaptic transmission	3.32	14.20	1.02e-27
PCu	MP	MP:0002752	abnormal somatic nervous system morphology	5.06	5.56	2.62e-02
PCu	MP	MP:0000788	abnormal cerebral cortex morphology	8.59	6.41	2.04e-02
PCu	MP	MP:0000801	abnormal temporal lobe morphology	8.91	6.56	1.59e-02
PCu	MP	MP:0000807	abnormal hippocampus morphology	9.07	6.63	1.43e-02

© 2022 Williams JA et al. *JAMA Psychiatry*.

PCu	MP	MP:0004166	abnormal limbic system morphology	10.70	8.48	1.55e-04
Pu	DO	DOID:0060040	pervasive developmental disorder	5.13	5.52	4.50e-02
Pu	DO	DOID:0060037	developmental disorder of mental health	3.83	5.29	3.43e-02
Pu	DO	DOID:1561	cognitive disorder	2.85	5.03	2.90e-02
Pu	DO	DOID:150	disease of mental health	2.77	6.06	3.72e-04
Pu	GOBP	GO:0008542	visual learning	12.30	7.23	1.27e-02
Pu	GOBP	GO:0007626	locomotory behavior	9.87	7.52	2.21e-03
Pu	GOBP	GO:0007616	long-term memory	19.40	9.40	7.36e-04
Pu	GOBP	GO:0007268	chemical synaptic transmission	6.45	7.51	2.00e-04
Pu	MP	MP:0000104	abnormal sphenoid bone morphology	7.87	6.04	4.50e-02
Pu	MP	MP:0002133	abnormal respiratory system physiology	2.69	4.86	4.50e-02
Pu	MP	MP:0001473	reduced long term potentiation	5.94	5.78	3.51e-02

© 2022 Williams JA et al. *JAMA Psychiatry*.

Pu	MP	MP:0001392	abnormal locomotor behavior	2.24	4.77	3.35e-02
Pu	MP	MP:0001463	abnormal spatial learning	4.88	5.62	2.86e-02
Pu	MP	MP:0001402	hypoactivity	3.48	5.24	2.86e-02
Pu	MP	MP:0003313	abnormal locomotor activation	2.50	4.91	2.86e-02
Pu	MP	MP:0003948	abnormal gas homeostasis	3.37	5.27	2.33e-02
Pu	MP	MP:0002314	abnormal respiratory mechanics	8.79	6.48	2.21e-02
Pu	MP	MP:0003062	abnormal coping response	11.30	6.88	2.08e-02
Pu	MP	MP:0000106	abnormal basisphenoid bone morphology	11.30	6.88	2.08e-02
Pu	MP	MP:0003491	abnormal voluntary movement	2.23	4.90	1.88e-02
Pu	MP	MP:0002069	abnormal consumption behavior	3.17	5.28	1.76e-02
Pu	MP	MP:0002915	abnormal synaptic depression	7.11	6.53	8.17e-03
Pu	MP	MP:0005105	abnormal middle ear ossicle morphology	10.40	7.19	6.95e-03

© 2022 Williams JA et al. *JAMA Psychiatry*.

Pu	MP	MP:0003008	enhanced long term potentiation	10.60	7.26	6.13e-03
Pu	MP	MP:0002062	abnormal associative learning	4.78	6.06	6.13e-03
Pu	MP	MP:0003107	abnormal response to novelty	4.94	6.21	4.09e-03
Pu	MP	MP:0009747	impaired behavioral response to xenobiotic	9.29	7.24	3.56e-03
Pu	MP	MP:0010029	abnormal basicranium morphology	9.40	7.30	3.23e-03
Pu	MP	MP:0001363	increased anxiety-related response	6.24	7.02	8.99e-04
Pu	MP	MP:0001898	abnormal long term depression	9.60	7.91	6.13e-04
Pu	MP	MP:0002206	abnormal CNS synaptic transmission	3.68	6.55	2.45e-04
Pu	MP	MP:0005272	abnormal temporal bone morphology	13.40	9.01	2.00e-04
Pu	MP	MP:0009745	abnormal behavioral response to xenobiotic	5.45	7.22	1.96e-04
Pu	MP	MP:0001899	absent long term depression	17.80	9.81	1.55e-04
Pu	MP	MP:0003063	increased coping response	26.90	11.20	8.99e-05

© 2022 Williams JA et al. *JAMA Psychiatry*.

Pu	MP	MP:0002207	abnormal long term potentiation	6.29	7.69	8.58e -05
Pu	MP	MP:0003635	abnormal synaptic transmission	3.71	7.07	2.66e -05
Pu	MP	MP:0001362	abnormal anxiety-related response	5.57	7.85	1.92e -05
Pu	MP	MP:0003633	abnormal nervous system physiology	2.53	6.60	1.39e -05
Pu	MP	MP:0002065	abnormal fear/anxiety-related behavior	5.64	8.41	2.04e -06
Pu	MP	MP:0002572	abnormal emotion/affect behavior	4.66	8.66	9.81e -08
Pu	MP	MP:0014114	abnormal cognition	4.87	9.49	2.21e -09
Pu	MP	MP:0002063	abnormal learning/memory/conditioning	4.88	9.50	2.13e -09
SFG	MP	MP:0004166	abnormal limbic system morphology	6.73	5.92	3.31e -02
SFG	MP	MP:0000801	abnormal temporal lobe morphology	7.49	6.34	1.51e -02
SFG	MP	MP:0000807	abnormal hippocampus morphology	7.62	6.41	1.35e -02
VeI_IV	GOBP	GO:0007626	locomotory behavior	6.31	6.04	1.96e -02

© 2022 Williams JA et al. *JAMA Psychiatry*.

VeI_IV	GOBP	GO:0010842	retina layer formation	14.30	7.94	3.72e -03
VeI_IV	GOBP	GO:0006810	transport	4.09	6.58	4.50e -04
VeI_IV	MP	MP:0000966	decreased sensory neuron number	3.96	5.23	4.90e -02
VeI_IV	MP	MP:0002102	abnormal ear morphology	2.80	4.91	4.50e -02
VeI_IV	MP	MP:0003492	abnormal involuntary movement	2.20	4.74	3.96e -02
VeI_IV	MP	MP:0002067	abnormal sensory capabilities/reflexes/nociception	2.21	4.77	3.60e -02
VeI_IV	MP	MP:0003051	curly tail	6.79	5.94	3.43e -02
VeI_IV	MP	MP:0005377	hearing/vestibular/ear phenotype	2.42	4.88	3.19e -02
VeI_IV	MP	MP:0001406	abnormal gait	2.94	5.16	2.17e -02
VeI_IV	MP	MP:0000913	abnormal brain development	2.39	4.97	2.13e -02
VeI_IV	MP	MP:0003633	abnormal nervous system physiology	1.82	4.74	2.04e -02
VeI_IV	MP	MP:0000808	abnormal hippocampus development	11.30	6.90	1.72e -02

© 2022 Williams JA et al. *JAMA Psychiatry*.

VeI_IV	MP	MP:0000872	abnormal cerebellum external granule cell layer morphology	6.47	6.14	1.59e-02
VeI_IV	MP	MP:0002081	perinatal lethality	2.03	4.89	1.59e-02
VeI_IV	MP	MP:0003861	abnormal nervous system development	2.12	5.07	8.99e-03
VeI_IV	MP	MP:0004415	abnormal cochlear nerve compound action potential	12.60	7.38	8.58e-03
VeI_IV	MP	MP:0002855	abnormal cochlear ganglion morphology	6.24	6.36	7.36e-03
VeI_IV	MP	MP:0002272	abnormal nervous system electrophysiology	3.41	5.63	6.95e-03
VeI_IV	MP	MP:0002058	neonatal lethality	2.30	5.24	6.13e-03
VeI_IV	MP	MP:0006108	abnormal hindbrain development	4.33	6.07	3.88e-03
VeI_IV	MP	MP:0008948	decreased neuron number	3.17	5.83	2.33e-03
VeI_IV	MP	MP:0002152	abnormal brain morphology	2.00	5.33	2.25e-03
VeI_IV	MP	MP:0000857	abnormal cerebellar foliation	6.31	6.76	2.17e-03
VeI_IV	MP	MP:0003703	abnormal vestibulocochlear ganglion morphology	16.60	8.64	1.39e-03

© 2022 Williams JA et al. *JAMA Psychiatry*.

VeI_IV	MP	MP:0002882	abnormal neuron morphology	2.08	5.52	1.14e-03
VeI_IV	MP	MP:0000959	abnormal somatic sensory system morphology	2.74	5.90	8.17e-04
VeI_IV	MP	MP:0009964	abnormal cerebellum lobule morphology	6.31	7.09	7.36e-04
VeI_IV	MP	MP:0001393	ataxia	3.82	6.39	6.13e-04
VeI_IV	MP	MP:0000854	abnormal cerebellum development	5.43	6.94	5.31e-04
VeI_IV	MP	MP:0005402	abnormal action potential	6.01	7.16	4.50e-04
VeI_IV	MP	MP:0001081	abnormal cranial ganglia morphology	5.17	6.94	3.96e-04
VeI_IV	MP	MP:0000886	abnormal cerebellar granule layer morphology	6.11	7.24	3.56e-04
VeI_IV	MP	MP:0008946	abnormal neuron number	3.12	6.27	3.51e-04
VeI_IV	MP	MP:0003312	abnormal locomotor coordination	2.87	6.20	2.90e-04
VeI_IV	MP	MP:0002752	abnormal somatic nervous system morphology	2.54	6.08	2.57e-04
VeI_IV	MP	MP:0002184	abnormal innervation	4.49	6.89	2.21e-04

© 2022 Williams JA et al. *JAMA Psychiatry*.

VeI_IV	MP	MP:0000960	abnormal sensory ganglion morphology	4.49	6.89	2.21e -04
VeI_IV	MP	MP:0001516	abnormal motor coordination/ balance	2.72	6.36	1.02e -04
VeI_IV	MP	MP:0009956	abnormal cerebellar layer morphology	4.11	6.97	8.99e -05
VeI_IV	MP	MP:0004097	abnormal cerebellar cortex morphology	4.19	7.61	6.54e -06
VeI_IV	MP	MP:0000847	abnormal metencephalon morphology	3.74	7.66	2.08e -06
VeI_IV	MP	MP:0000849	abnormal cerebellum morphology	3.89	7.77	1.72e -06
VeI_IV	MP	MP:0000841	abnormal hindbrain morphology	3.58	7.65	1.55e -06

© 2022 Williams JA et al. *JAMA Psychiatry*.

eTable 16. Brain imaging measure *z* scores

Brain Region	Z-score	P Value	Brain Region	Z-score	P Value
**Middle Temporal Gyrus, temporooccipital part (right)**	5.76	**p <1.1×10-4**	Mean thickness of G-precuneus (left)	-3.59	p < 0.001
Inferior Temporal Gyrus, posterior Divisions (right)	3.38	p <0.001	Mean thickness of Pole-occipital (left)	-3.61	p < 0.001
Inferior Temporal Gyrus, posterior Divisions (left)	3.73	p <0.001	**Mean thickness of G-front-sup (lef**t)	-5.11	**p < 1.1×10-4**
**Temporal Fusiform Cortex, posterior division (right)**	4.70	**p < 1.1×10-4**	Mean thickness of S-parieto-occipital (left)	-3.34	p < 0.001
**Temporal Fusiform Cortex, posterior division (left)**	4.20	**p < 1.1×10-4**	Mean thickness of S-pericallosal (right)	3.32	p < 0.001
Frontal Operculum Cortex (right)	-3.59	p <0.001			
Putamen (right)	-3.78	p <0.001			
I-IV Cerebellum (right)	-3.64	p <0.001			

Legend: Z scores represent the genetic association of IL-6/IL-6R with GMV or CT derived measure weighted average association scaled by the genetic association with the biomarker measure. Estimates are reported as Z-scores, where a positive Z-score represents that genetically-predicted levels of the biomarker were positively associated with the brain imaging measure. Significance are given with multiply corrected p value threshold of 1.1×10^{-4} . Results significant at multiply-corrected level indicated in bold. Red colour denotes a positive association and blue colour denotes a negative association ie increased IL6/IL6R associated with increased volume in red or decreased volume or thickness in blue. Note the gene region for IL-6/IL-6R alleles are associated with increased circulating IL-6 but also decrease in IL-6 signalling. Thus, results could be interpreted as suggestive of elevation of IL-6/IL6R and reduction in GMV in the Middle Temporal and Fusiform Gyrus and increased CT in the Frontal-Superior region. See Figure 3 for a visualization of these regions.

© 2022 Williams JA et al. *JAMA Psychiatry*.
