# Supplementary material for: Inflammation and Brain Structure in Schizophrenia and Other Neuropsychiatric Disorders: A Mendelian Randomization Study
Source: JAMA Psychiatry. 2022 Mar 30;79(5):498–507. doi: 10.1001/jamapsychiatry.2022.0407 (PMC8968718; doi:10.1001/jamapsychiatry.2022.0407)
Supplement: Supplement 2. — PIMS Collaboration. [file jamapsychiatry-e220407-s002.pdf]

Supplemental Online Content: Nonauthor Collaborators

*Indicates required information. Only first name, last name, and suffix will appear in PubMed.

*Group Name(s): PIMS Collaboration							
***First Name and Middle Initial(s)**	***Last Name**	***Suffix (eg, Jr, III)**	Academic Degrees	Institution	Location (city, state/province, country)	Role or Contribution, eg, chair, principal investigator	Group (if more than 1 Group listed in the byline) and/or Subgroup (eg, Steering Committee)
Jack C	Rogers		BA, MSc, PhD	University of Birmingham	Birmingham, West Midlands, UK	collaborator	
Valeria	Mondelli		MD, PhD	King’s College London, Department of Psychological Medicine, Institute of Psychiatry, Psychology & Neuroscience, London, UK. 2) National Institute for Health Research (NIHR) Maudsley Biomedical Research Centre, South London and Maudsley NHS Foundation Trust, King's College London, London, UK.	London, UK	collaborator	
Paola	Dazzan		MD, PhD	King’s College London, Department of Psychological Medicine, Institute of Psychiatry, Psychology & Neuroscience, London	London, UK	collaborator	
Carmine	Pariante		MD, PhD	Psychiatry, Psychology & Neuroscience, London, UK. 2) National Institute for Health Research (NIHR) Maudsley Biomedical Research Centre, South London and Maudsley NHS Foundation Trust, King's College London, London, UK.	London, UK	collaborator	
James	MacCabe		MBBS, PhD	King’s College London, Department of Psychological Medicine, Institute of Psychiatry, Psychology & Neuroscience, London	London, UK	collaborator	
Alice	Egerton		PhD	King’s College London, Department of Psychological Medicine, Institute of Psychiatry, Psychology & Neuroscience, London	London, UK	collaborator	
Peter	Jones		PhD	Department of Psychiatry, University of Cambridge School of Clinical Medicine, Cambridge, EnglandCambridgeshire and Peterborough NHS Foundation Trust, Cambridge, England;	Cambridge, UK	collaborator	
Ed	Bullmore		MB, PhD	Department of Psychiatry, University of Cambridge School of Clinical Medicine, Cambridge, EnglandCambridgeshire and Peterborough NHS Foundation Trust, Cambridge, England;	Cambridge, UK	collaborator	
Nikos	Koutsouleris		MD, PhD	Ludwig Maximilians University and King's College London	Munich, Germany	collaborator	
Eva	Meisenzahl		MD, PhD	Department of Psychiatry and Psychotherapy Heinrich-Heine-University Dusseldorf	Dusseldorf, Germany	collaborator	
David	Cotter		MBBCH, PhD	Royal College of Surgeons Ireland, Psychiatry	Dublin, Ireland	collaborator	
Neil	Harrison		MBBS PhD	Cardiff University, Brain Research Imaging Centre	Cardiff, UK	collaborator	
